# Disentangling the drivers and host-mediated global spread of H7 influenza A virus

**DOI:** 10.1038/s41467-026-72718-9

**Published:** 2026-05-06

**Authors:** Rongrong Qu, Luming Yang, Siqing Li, Can Chen, Huihui Zhang, Kexin Cao, Wenkai Zhou, Mengsha Chen, Jiani Miao, Jiaxing Qi, Qianqian Feng, Jiaxin Chen, Anqi Dai, Xiaoyue Wu, Jingtong Zhou, Shigui Yang

**Affiliations:** 1 Department of Emergency Medicine of Second Affiliated Hospital, School of Public Health, Zhejiang University School of Medicine, Hangzhou, China; 2 Department of Electrical and Computer Engineering, College of Engineering, The Ohio State University, Columbus, OH USA; 3 Department of Statistics and Data Science, College of Science, Southern University of Science and Technology, Shenzhen, China

**Keywords:** Ecological epidemiology, Viral epidemiology, Epidemiology, Microbial ecology

## Abstract

Avian influenza H7 viruses pose a significant zoonotic and pandemic threat, yet their evolutionary dynamics, spatial transmission patterns, and host-specific roles remain underexplored. This study integrates phylodynamic and phylogeographic analyses to map global H7 dissemination, quantify host-specific contributions, and identify key ecological and anthropogenic drivers. Epidemiological data show key epidemic waves in Asia during 2013-2014 and 2016-2017, and in Africa in 2023. The Eurasian and American lineages of H7 viruses exhibit transmission with a median velocity of ~661 km/year and ~354 km/year, though spread varies significantly by virus subtype. Anseriformes (~587 km/year) and wild birds (~654 km/year) spread the Eurasian lineage of H7 viruses more rapidly and over greater distances than Galliformes and domestic birds. Geographic distance is negatively associated with the spread of the H7 virus, while temperature and poultry density show positive association. In this work, we identify Asia as an important H7 virus evolutionary epicenter. Anseriformes drives transcontinental spread, whereas Galliformes facilitates local amplification. The dynamics of the H7 virus are shaped by ecological and socioeconomic factors. A One Health approach emphasizing targeted surveillance and global cooperation is essential to mitigate cross-species transmission and future pandemic threats.

## Abstract (web summary)

Authors show that spread of H7 avian influenza viruses is driven by geographic distance and temperature. Anseriformes, or waterfowl, propel long-range dissemination, while Galliformes, or land fowl, facilitate local amplification.

## Introduction

Avian influenza viruses (AIVs) pose a persistent threat to both animals and human health due to their potential to cause zoonotic infections and evolve into highly pathogenic forms^1–3^. The H7 subtype has been responsible for several outbreaks in poultry and sporadic but severe human infections, most notably the H7N9 epidemic, resulting in over 1687 cases globally^4,5^. Historically, the major high-pathogenic avian influenza (HPAI) virus lineages are known to have originated from previously low-pathogenic avian influenza (LPAI) strains^6^. Notably, a major lineage of HPAI H5, clade 2.3.4.4b viruses, is the cause of an epizootic in wild bird reservoirs with widespread infections across 84 countries in Asia, Africa, Europe, and North America^7,8^. LPAI viruses of the H5 and H7 subtypes can evolve into HPAI viruses through the acquisition of a multi-basic cleavage site at the HA1-HA2 junction, a process that most commonly occurs via insertion events^9,10^. Notably, the region of cleavage site at the HA1-HA2 junction is distinct from the receptor-binding site; with respect to receptor binding, currently circulating H5N1 clade 2.3.4.4b viruses retain avian-like receptor specificity, limiting their immediate pandemic potential despite high virulence in birds^11^. While much attention has focused on the H5 subtype, the transmission dynamic and ecological drivers of H7 viruses remain comparatively underexplored^12,13^.

As natural reservoirs of AIVs, wild birds, particularly Anseriformes (ducks, swans, and geese), play a critical role in long-distance viral dispersal through migratory pathways^14^. By introducing novel gene segments across regions, they facilitate the geographic spread and genetic diversification of avian influenza viruses^15^. This poses a risk of spillover to domestic poultry, which are often in close contact with humans^16,17^, therefore, they remain key targets for global influenza surveillance and early warning systems. The Galliformes (pheasants, turkeys, peafowl, and quail) often exhibit synanthropic behavior and evidence has shown that many species in this order are susceptible to and can shed AIVs^18^. Galliformes have the potential to act as bridge hosts, as agricultural areas may attract wild individuals searching for food resources or conspecifics. Moreover, differences in pathogenicity, transmission efficiency, and evolutionary dynamics across avian hosts highlight the importance of clarifying host-specific transmission pathways, particularly at the expanding human-animal interface, to better predict and prevent future spillover events^19^.

To better understand the roles of different host groups in the global dissemination and cross-species transmission of H7 subtypes, particularly regarding the differential transmission rates of LPAI and HPAI H7 viruses, a systematic analysis of transmission patterns across different host groups is needed. Furthermore, spatial diffusion of AIVs is shaped by a complex interplay of ecological and anthropogenic factors, including bird migration, poultry trade, climate conditions, and human activity, yet the specific mechanisms influencing the dissemination of H7 viruses are not well characterized. Despite the recognized pandemic potential of H7 viruses, there is a lack of integrative studies that simultaneously assess their evolutionary dynamics, spatial transmission patterns, and host-specific roles on a global scale^20^.

To address these gaps, this study employs an integrated phylodynamic and phylogeographic approach to reconstruct the global evolutionary and spatial transmission patterns of H7 and its major subtypes (H7N3, H7N7, H7N9); quantifies the relative contributions of key avian host groups to viral spread, specifically investigating the differences in dispersal velocity of LPAI and HPAI H7 viruses among these hosts; and identifies ecological and anthropogenic predictors of transmission using a generalized linear modeling (GLM) framework. These efforts aim to advance our understanding of the ecology of H7 viruses and provide evidence-based strategies for targeted surveillance and cross-sectoral prevention under a One Health paradigm.

## Results

### Epidemiology of avian influenza H7 virus

**Fig. 1:**
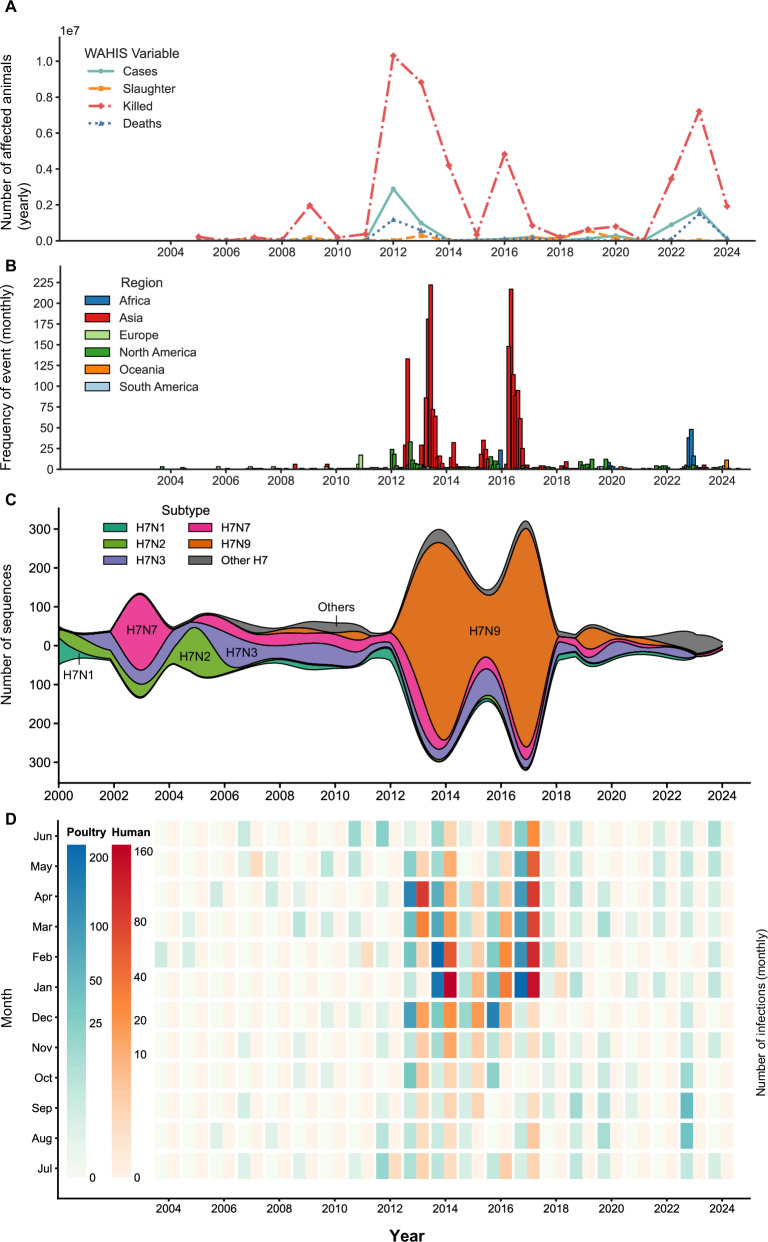
H7 outbreak event and sequences. **A** Annual number of affected animals from the World Animal Health Information System (WAHIS). Cases refer to the number of confirmed outbreaks; Slaughter indicates the total number of animals slaughtered for outbreak management purposes (e.g. to prevent further transmission); Killed refers to animals actively culled to control the epidemic; Deaths represent the number of animals that died due to the disease. **B** Monthly time series of H7 outbreaks reports across all animal types by geographic region, as reported to the Food and Agriculture Organization of the United Nations (FAO). **C** Temporal trends of H7 *HA* sequences submitted to the GISAID and NCBI Influenza Virus Resource databases from January 2000 to November 2024. Time series describing H7 outbreaks in all animal types; the lower panel focuses on poultry-specific outbreaks. **D** Monthly number of H7 outbreaks in poultry (left y-axis) and confirmed human H7 infection cases (right y-axis), as reported to the FAO. Source data are provided as a Source Data file.

Outbreaks of H7 avian influenza have been reported across all six continents, with Asia exhibiting the highest frequency. Three major outbreak peaks were observed during the periods 2013–2014, 2016–2017, and 2023 (Fig. 1A, Fig. 1B). Notably, the outbreaks from 2013 to 2017 were predominantly driven by H7N9 viruses, as evidenced by the marked increase in outbreak events (Fig. 1B), a surge in viral sequence submissions to the Global Initiative on Sharing All Influenza Data (GISAID) and the National Center for Biotechnology Information (NCBI) Influenza Virus Resource databases (Fig. 1C), and a concurrent sharp rise in reported human infection cases (Fig. 1D). Moreover, avian outbreaks and human cases exhibited clear seasonal synchronization, with most events occurring between December and the following May. In addition, from 2000 to 2024, H7N3 and H7N7 viruses were continuously reported, with H7N7 showing a distinct peak in 2003. Although overall report numbers remained relatively low, H7N6 demonstrated a noticeable rise in both sequence submissions and outbreak frequency in 2023 (Supplementary Fig. 1).

Supplementary Fig. 2 presents the geographical and temporal distribution of H7 avian influenza virus sequences. Sequences were collected from 22 countries, with China, the United States, and the Netherlands contributing the most, highlighting their key roles in global H7 surveillance. Temporally, the number of sequences peaked in January, indicating a seasonal trend possibly associated with wild bird migration or increased poultry density during winter months. Sampling was stratified by country and year, with totals summarized in Supplementary Table 1. Supplementary Fig. 3 shows a relatively even spatiotemporal distribution of sequences, which could support unbiased and comprehensive analysis of virus dynamics. The uniform coverage across different regions and years provides a robust basis for analyzing the evolutionary trends and transmission patterns of the virus on a global scale. A strong temporal phylogenetic structure was observed (Supplementary Fig. 4). Multiple local spill over events of H7N9 from poultry to humans were observed between 2013 and 2017, and the inferred genetic diversity (Ne) showed a declining trend. This decrease may be attributed to a combination of viral lineage bottlenecks, effective control measures, and sampling biases. In contrast, the rising diversity of H7N3 may reflect sustained circulation in poultry in the Americas, despite limited human impact (Supplementary Fig. 5).

### Spatial diffusion of avian influenza H7 virus

For phylogenetic and phylogeographic analysis, we complied four data sets of *HA* gene (coding for the hemagglutinin surface protein) sequences from 2000 to 2024, from A: a sample of all H7 subtypes and hosts; B: H7N3 only; C: H7N7 only; D: H7N9 only. The ‘H7’ dataset contains all the subtypes. After subsampling these ranged in size from 430 to 814, please see Methods section for details.

**Fig. 2:**
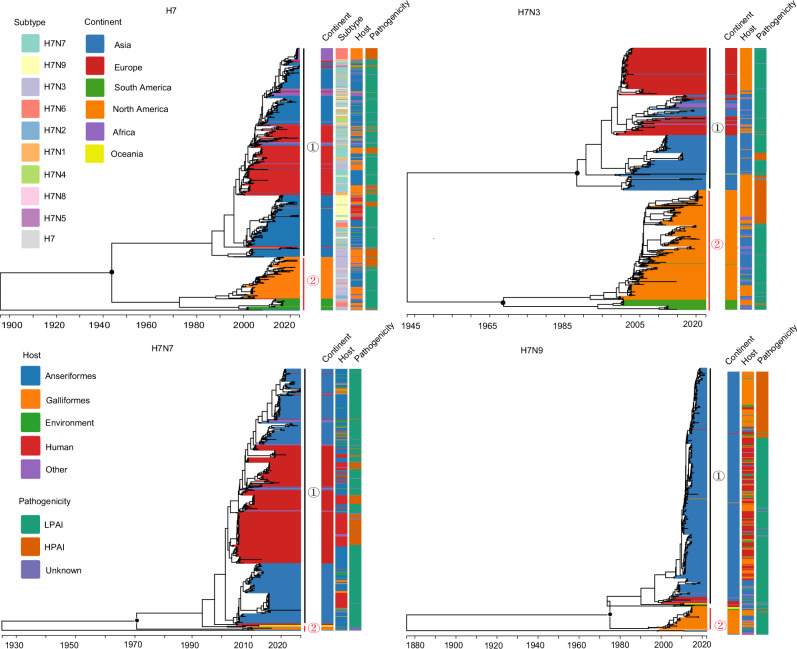
Time-scaled maximum clade credibility (MCC) tree of H7 *HA* genes sequences. The evolutionary relationships of the H7 *HA* gene segment were reconstructed using full-length sequences from viruses collected across different geographic regions. The geographic information was displayed in the end branch. The sequences in *HA* lineage $\textcircled{1}$ were mainly from Asia and Europe, so we named $\textcircled{1}$ as Eurasian lineage. The sequences in *HA* lineage $\textcircled{2}$ were from America, so we named $\textcircled{2}$ as American lineage.

The dissemination of AIV H7 displays distinct geographic patterns across broad regions. Globally, discrete trait phylogeographic analysis of the H7 *HA* gene segment showed predominantly unidirectional transmissions events. Current circulating H7 lineages appear to have spread from Asia to Europe and Africa, while North American sequences likely represent an independent gene pool (Supplementary Fig. 6). The phylogeographic reconstruction suggested that H7 viruses most likely originated in Asia (root state posterior probability = 0.84) (Supplementary Fig. 6A). The root of the H7N3 and H7N9 clades was inferred to be in Asia with posterior probability = 1.0 (Supplementary Fig. 6B, 6D), providing strong support for an Asian origin. In contrast, H7N7 most likely originated in Europe, with a root state posterior probability of 0.9927 (Supplementary Fig. 6C). As shown in Supplementary Fig. 6 and Fig. 2, the *HA* genes of H7 subtype viruses could be classified into two major lineages, Eurasian lineage and American lineage. These findings suggest that long-distance, cross-regional introductions of H7 viruses occur infrequently and usually in a one-way manner. In addition, viral sequences exhibited strong regional clustering, indicating that once introduced, H7 viruses tend to persist and evolve within specific geographic regions. Notably, the Maximum Clade Credibility (MCC) tree of the H7 *HA* gene showed that major geographical divergence began around 1943 (95% highest posterior density [HPD]: 1902−1969) (Supplementary Fig. 6A). For H7N3, two distinct lineages have circulated in South America, North America; Asia, and Europe, with divergence times estimated in 1970 (1952−1985) and 1997 (1972−1999), respectively (Supplementary Fig. 6B). In the case of H7N7, geographical separation was first observed in 1969 (1940−1988) and has predominantly persisted in Europe and Asia since 2000 (Supplementary Fig. 6C). As for H7N9, the MCC tree indicated Asia as the most probable origin and dominant region of circulation, with limited evidence of cross-regional spread. A divergence event with North America lineages is estimated to have occurred in 1976 (1966−1987) (Supplementary Fig. 6D). The information of subtype, host, continent and pathogenicity was presented in Fig. 2. These MCC trees were generated through Bayesian phylogenetic analysis, with node support indicated by posterior probability (Supplementary Fig. 7). Nodes with posterior probabilities above 0.95 are considered highly reliable, while those below 0.75 have lower support. The posterior probabilities supported the aforementioned divergence events all exceeded 0.95, indicating strong statistical support for these evolutionary patterns.

**Fig. 3:**
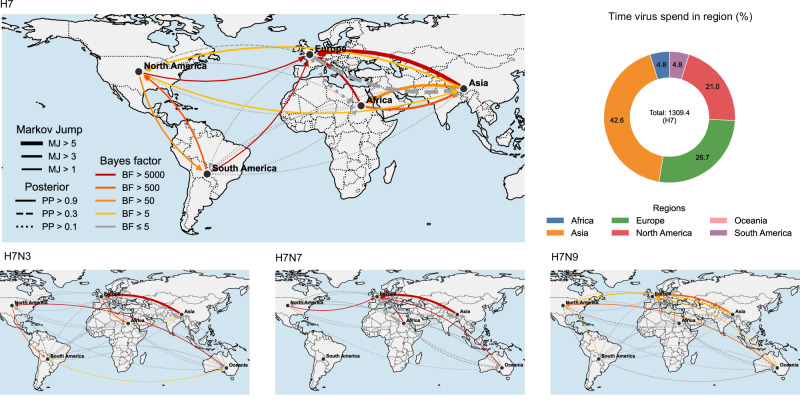
Contrasting geographic transmission patterns of H7 subtypes. Each panel illustrated spatial diffusion patterns inferred from discrete phylogeographic analysis. From left to right, the plots show regional Markov jump counts (transmission events), Markov rewards (residence times), and Bayes factors indicating the statistical support for transmission routes. Maps were generated using the R package rnaturalearth with data from Natural Earth (public domain, https://www.naturalearthdata.com).

To elucidate the principal spatial diffusion dynamics underlying the global spread of H7 viruses, the most strongly supported transition rates between discrete geographic locations were compiled using Bayesian stochastic search variable selection (BSSVS) and are summarized in Fig. 3. These rates represent inferred viral movements between all possible pairs of locations. The statistical support for each route was evaluated using Bayes factors (BF), with thresholds defined as follows: BF > 5 indicates positive support, BF > 50 strong support, BF > 500 very strong support, and BF > 5000 decisive support. Due to consistent migration patterns across datasets (Supplementary Table 2), results are presented from the epi-based dataset with balanced sampling. The Asia-Europe route showed the strongest support (all BF > 100). H7 viruses persisted longest in Asia (42.63% Markov rewards; 24% Markov jumps to Europe). Similarly, H7N3, H7N7 and H7N9 exhibited extended presence in Asia, with 28.38%, 24.88% and 19.40% Markov jumps from Asia to Europe, respectively, and these Markov jumps had high bayes factors (Fig. 3, Supplementary Tables 3, 4).

**Fig. 4:**
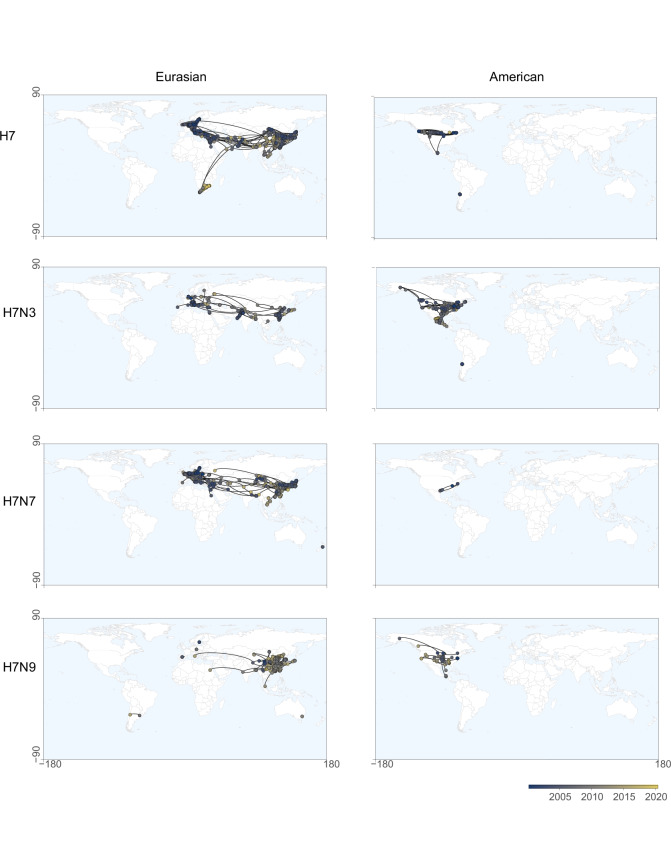
Continuous phylogeographic reconstruction of H7 and its subtypes. Spread of H7 virus and its subtypes from 2000 to 2024. Circles represent nodes in the maximum clade credibility phylogeny, colored by the inferred time of occurrence, with green indicating early time and red indicating late time. Maps were generated using the R package rnaturalearth with data from Natural Earth (public domain, https://www.naturalearthdata.com).

Continuous phylogeographic reconstruction provides a detailed depiction of the temporal and spatial transmission of H7 viruses across the transmission network (Fig. 4, Supplementary Fig. 8). The continuous phylogeography analysis suggests that Eurasian lineage of H7 viruses has two primary transmission trajectories. One transmission route showed westward spread of the virus from East Asia into Southeast and West Asia, while another indicated eastward movement from Western Europe into West Asia. By around 2010, the virus converged in West Asia before spreading to South Africa. For the American lineage, transmission occurred primarily in the northern United States along the Canadian border, with subsequent southward spread into Mexico. Overall, H7 viruses exhibited sustained and widespread transmission across multiple continents. The Eurasian lineages of H7N3 and H7N7 circulated actively within Eurasia, while the Eurasian lineage of H7N9 spread extensively in East Asia, particularly in China after 2015. The American lineage of the H7N3 virus has circulated widely across the United States, extending as far as Alaska, and over the past decade has spread southward into Mexico, where it remains actively transmitted. The American lineage of H7N9 exhibited a similar transmission pattern, whereas the H7N7 lineage showed comparatively limited circulation.

**Fig. 5:**
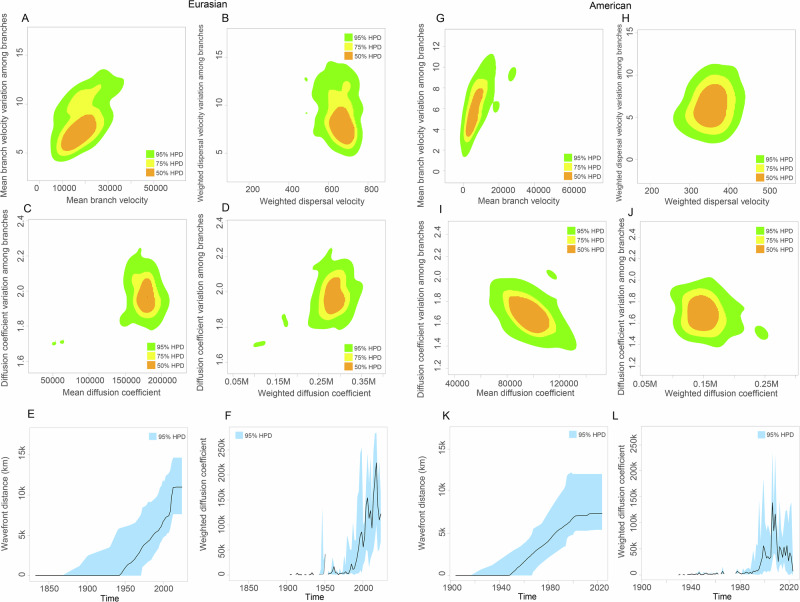
Estimated dispersal statistics for H7. The two columns on the left represent the Eurasian lineage, while the two columns on the right represent the American lineage. **A**, **G** Kernel density estimates of mean branch dispersal velocity parameters. **B**, **H** Kernel density estimates of weighted branch dispersal velocity parameters. **C**, **I** Kernel density estimates of original diffusion coefficient parameters. **D**, **J** Kernel density estimates of weighted diffusion coefficient parameters. **E**, **K** Furthest extent of epidemic wavefront (spatial distance from epidemic origin). **F**, **L** Evolution of weighted diffusion coefficient. For panels (**E**), (**F**), (**K**), and (**L**), solid lines represent the posterior median values of wavefront distance and diffusion coefficient, and shaded areas denote the 95% highest posterior density (HPD) intervals. All estimates were derived from Bayesian phylogeographic inference using 100 posterior trees obtained through Markov chain Monte Carlo (MCMC) sampling.

Furthermore, the spatial expansion of the Eurasian lineage and American lineage of H7 viruses in continuous space showed a median weighted branch dispersal velocity of 661.23 km/year (95% HPD: 400.12–733.47) and 354.18 km/year (260.13–444.50), respectively (Fig. 5B, H). Similarly, the median dispersal velocities for the Eurasian lineage of H7N3, H7N7, and H7N9 subtypes were estimated at 585.37 km/year (514.10–648.87), 645.28 km/year (512.61–747.96), and 562.48 km/year (380.76–689.73), respectively (Supplementary Figs. 9–11). Wavefront distance was defined as the mean great-circle distance from the epidemic origin to the most recently circulating lineages. The wavefront distance of H7 viruses showed a consistent upward trend over time (Fig. 5E, K), starting near zero and rising sharply after mid-20th century. In contrast, the weighted diffusion coefficient (Fig. 5F, L) exhibited a fluctuating upward pattern: it stayed negligible until ~1900, then increased with significant variability and spikes sharply by 2000. H7N3, H7N7, and H7N9 also follow a similar trend of change (Supplementary Figs. 9–11). Trends in wavefront distance and diffusion coefficients over the past two decades mirrored the overall historical trends (Supplementary Fig. 12). As of the latest estimate, the Eurasian lineage of H7 viruses exhibited a wavefront distance of 10,986.55 km (7625.84–14,640.81), compared with 7311.98 km (5380.59–11,971.32) for the American lineage (Fig. 5E, K). The diffusion coefficient was consistently higher for the Eurasian lineage than for the American lineage (Fig. 5F, L).

### Host transmission patterns

**Fig. 6:**
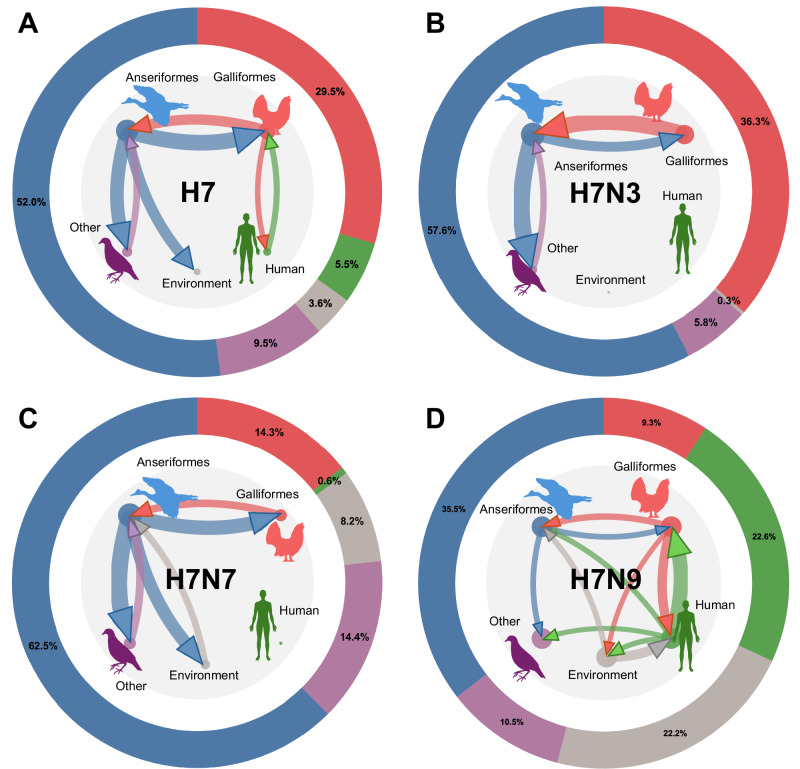
Contrasting host transmission patterns of H7 subtypes inferred from discrete-trait phylogenetic analysis. **A** H7; **B** H7N3; **C** H7N7; **D** H7N9. Lines between hosts represent Markov jumps, with line thickness proportional to the number of transitions. Numbers on the circular ring indicate Markov rewards (time spent in each host). Ring colors correspond to host categories shown at the center. Credit: all silhouettes in (**A**–**D**) were obtained from PhyloPic and PhyloPic is under a Creative Commons CC0 license. Images of Homo sapiens sapiens and Zentrygon were obtained from Katy Lawler and Natalia Ladino, respectively, under the Creative Commons Attribution 4.0 International license (https://creativecommons.org/licenses/by/4.0/). Color modifications were made to the original images.

Host-switching dynamics of H7 viruses were analyzed using a discrete diffusion model, focusing on transmission between avian orders, Anseriformes and Galliformes, as well as environmental, humans, and other hosts-derived samples. Markov jumps and Markov rewards were used to quantify transmission events and time spent within each host group (Fig. 6, Supplementary Tables 5, 6). Overall, H7 viruses and their subtypes exhibited longer persistence in Anseriformes, suggesting greater adaptation or replication efficiency in this host group. This sustained presence may facilitate viral evolution and cross-species transmission. In H7 phylogenies, transmission events from Anseriformes to Galliformes occurred about twice as frequently as the reverse (Fig. 6). A similar trend was observed in different lineage of H7N3 and H7N7 subtypes, where transmissions from Anseriformes to Galliformes were three times more frequent (Supplementary Table 5), indicating subtype-specific host transmission dynamics. Notably, H7N9 virus demonstrated a distinct pattern, with prolonged persistence in humans (22.62% Markov rewards) and bidirectional transmission between human and Galliformes, 26.43% of Markov jumps from human to Galliformes and 13.88% in the reverse direction (Fig. 6). The Eurasian lineage of the H7N9 virus exhibited a similar pattern (Supplementary Tables 5, 6). We also analyzed the transmission dynamics of hosts, classified as either wild or domestic, which included samples from Galliformes and Anseriformes. H7 viruses exhibited more frequent host transmissions between wild and domestic birds, with a slightly longer duration in wild birds than in domestic poultry (Supplementary Fig. 13; Supplementary Tables 7, 8). In H7N9, particularly the Eurasian lineage, transmission from humans to domestic birds was more common than between wild and domestic birds. Compared with other subtypes, humans also displayed higher Markov rewards, highlighting their prominent role in virus transmission.

**Fig. 7:**
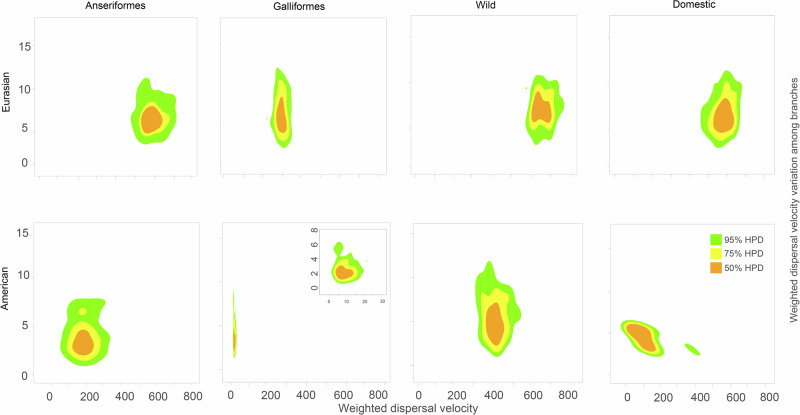
Host-specific dispersal velocity estimates for H7. The top row represents the Eurasian lineage, while the bottom row represents the American lineage. Each panel shows dispersal velocities stratified by host type. Shaded contours represent the 50%, 75%, and 95% highest posterior density (HPD) regions, estimated via kernel density methods, with darker shades indicating higher certainty.

To evaluate host contributions to spatial transmission, we conducted a combined analysis integrating discrete host states with continuous geographic data. The Eurasian lineage of H7 viruses spread more rapidly among Anseriformes (586.51 km/year, 518.90–682.14) and wild birds (654.60 km/year, 595.14–751.59) than among other hosts, whereas Galliformes exhibited the slowest dispersal velocity (294.44 km/year, 253.42–343.79) (Fig. 7). This pattern was consistent across subtypes: Anseriformes displayed significantly higher dispersal velocity than Galliformes in H7N3 (Mann–Whitney U test, *p* < 0.01), H7N7 (*p* < 0.01), and H7N9 (*p* < 0.01) (Supplementary Figs. 14–16; Supplementary Table 9). Wild birds generally transmitted viruses faster than domestic poultry in H7, H7N3, and H7N9 (*p* < 0.01), whereas in the Eurasian lineage of H7N7, domestic birds contributed more to the rapid transmission than wild birds (*p* < 0.01) (Supplementary Figs. 14–16, Supplementary Table 9). Results based on mean branch dispersal velocities were consistent with weighted estimates (Supplementary Fig. 17).

**Fig. 8:**
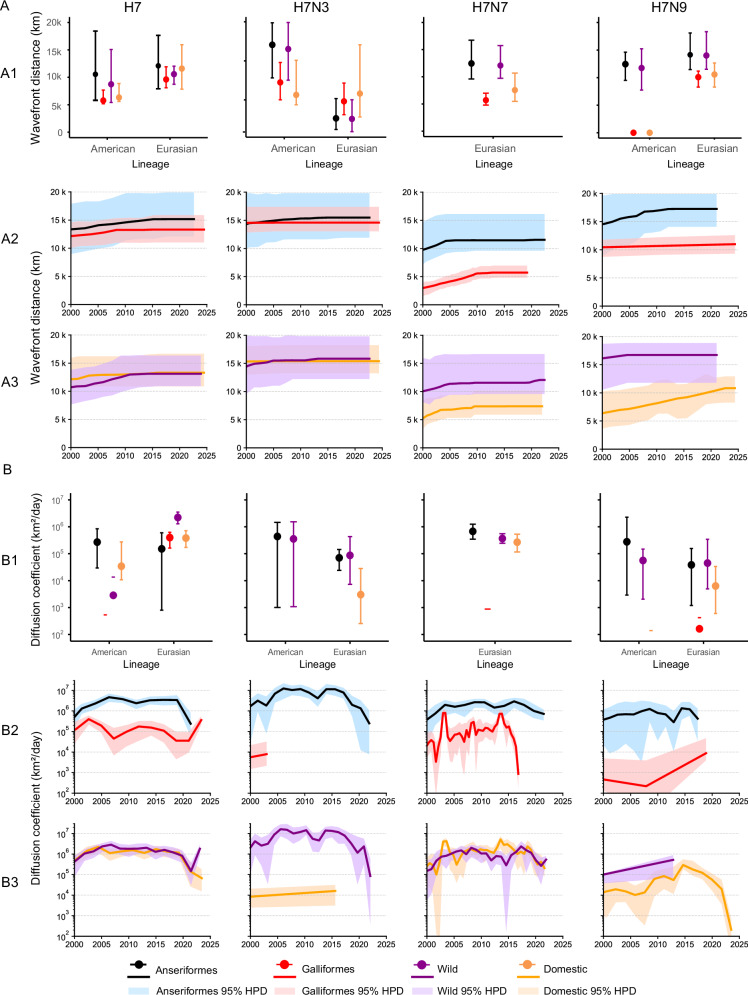
Host-specific wavefront distance and diffusion coefficient estimates for H7 and its subtypes. **A** Estimated wavefront distances (km) for H7 virus and subtypes. A1 shows the final spatial extent of spread for different viral lineages, while A2 and A3 illustrate the temporal dynamics of wavefront distances without considering lineages; **B** Weighted diffusion coefficient (km²/day). B1 presents the final diffusion coefficient, and B2 and B3 illustrate the temporal dynamics of diffusion coefficient without considering lineages. Estimates were derived from phylogenetic analyses performed separately for each subtype, using the following numbers of independent virus isolates: H7 (*n* = 814), H7N3 (*n* = 462), H7N7 (*n* = 495), and H7N9 (*n* = 430), collected from 2000 to 2025. No technical replicates were included in the analysis. Groups were defined by host order (Anseriformes/Galliformes) and lineage (American/Eurasian lineages). Points in A1 and B1 represent the final wavefront distance and final diffusion coefficient for distinct viral subtypes, with error bars indicating the 95% highest posterior density (HPD). Solid lines in A2, A3, B2, and B3 represent the posterior median values of wavefront distance (A2, A3) and diffusion coefficient (B2, B3), and shaded areas denote the 95% highest posterior density (HPD) intervals based on 100 posterior trees obtained through Markov chain Monte Carlo (MCMC) sampling. Diffusion estimates for certain years are omitted due to irregular values likely resulting from limited sampling. Source data are provided as a Source Data file.

Wavefront distance and diffusion coefficient were estimated using the continuous diffusion model (Fig. 8). Wavefront distance varied by host type: in the latest estimates, the Eurasian lineage of H7 exhibited a wavefront distance of 12,096.978 km (7901.05–12,096.978) in Anseriformes, exceeding that of Galliformes (9,609.90 km, 8097.98–11,927.54), and a similar pattern was observed for the American lineage (Fig. 8A1). Notably, the American lineage of H7 viruses exhibited longer wavefront distance in domestic birds than wild birds, while whereas the opposite was observed for the Eurasian lineage (Fig. 8A1). From the perspective of temporal trends, Anseriformes and wild birds played a more prominent role in the dissemination of the H7 virus, as is also the case for subtypes (Fig. 8A2, 8A3). Overall, the result of final diffusion coefficient also showed Anseriformes and wild birds play a more important role in H7 transmission (Fig. 8B1). The diffusion coefficient was consistently higher for viruses originating from Anseriformes than from Galliformes for H7 and subtypes (Fig. 8B2). The diffusion coefficient of H7 viruses among wild and domestic birds was similar (Fig. 8B3). The variation trend of H7N3 and H7N9 diffusion coefficient is consistent, while the diffusion coefficient of H7N7 was higher in domestic birds than in wild birds during a specific period (Fig. 8B2, 8B3). These patterns were supported by mean diffusion coefficient estimates (Supplementary Figs. 18, 19).

In addition, Anseriformes-wild populations exhibited the fastest dispersal velocity (median: 580.89 km/year), followed by Anseriformes-domestic populations (437.20 km/year). Galliformes-domestic populations showed moderate velocity (216.37 km/year), while Galliformes-wild populations displayed the slowest velocity (46.88 km/year) (Supplementary Fig. 20). This result highlights Anseriformes-wild populations represent the most efficient drivers of long-range virus transmission.

Furthermore, dispersal velocity analysis revealed a clear pattern in the transmission of high and low-pathogenic H7 viruses (Supplementary Table 10). Overall, H7 viruses exhibited higher dispersal velocities in Anseriformes than in Galliformes, and in wild birds compared with domestic birds. Among domestic hosts, HPAI viruses generally spread more slowly than LPAI viruses. This difference was particularly pronounced for the H7N3 subtype, where HPAI viruses transmitted at a median velocity of 37.30 km/year (21.77–55.83) in domestic birds, approximately one-seventh the rate of LPAI viruses (282.04 km/year, 218.76–376.17). In contrast, within Galliformes, HPAI viruses tended to disperse faster than LPAI viruses. However, due to limited data for Anseriformes and wild birds, direct comparisons between HPAI and LPAI viruses in these groups remain inconclusive.

Early H7 transmission between Asia, Europe, and North America was mainly driven by Anseriformes, while Galliformes contributed to spread toward South Africa (Supplementary Fig. 21). Anseriformes were also key in the intra-Eurasian spread of H7N3, H7N7, and H7N9, except for H7N7 spread from the Middle East, which involved Galliformes. While wild and domestic birds contributed similarly to H7 spread overall, wild birds played a greater role in spreading H7N3, H7N7, and H7N9 (Supplementary Fig. 22).

### Mechanisms of virus spatial spread

**Fig. 9:**
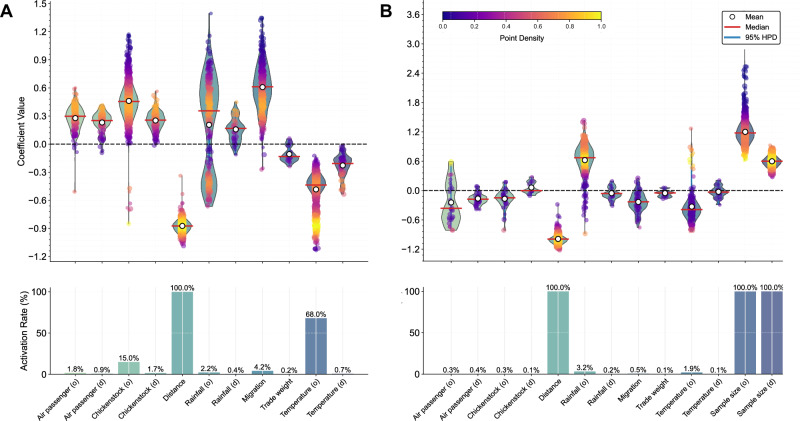
Predictors of global H7 virus diffusion among five geographic regions. Analyzed predictors include average inter-location distance, monthly airline passenger volume (2010–2018), bird migration routes, live poultry trade volume (1996–2016), chicken stock (2000–2023), annual mean temperature, annual precipitation and sample size. “o” and “d” indicate origin and destination predictors, respectively. Predictor support is expressed as inclusion probability based on indicator expectations. **A** Model excluding sample size; **B** Model including sample size as a distinctive predictor. Bars show the mean and 95% highest posterior density (HPD) of generalized linear modeling (GLM) coefficient (β) on a log scale, conditioned on the inclusion of each predictor.

A Bayesian phylogeographic GLM was used to assess potential predictors of spatial diffusion of H7 viruses (Fig. 9). Spearman rank correlation analysis moderate correlation between Gross domestic product (GDP) per capita and other variables (GDP per capita and airline passenger volume: *r* = 0.54; GDP per capita and sample size: *r* = 0.47; GDP per capita and temperature: *r* = −0.65) (Supplementary Fig. 23). The variance inflation factor did not identify multicollinearity. Following best practices for multicollinearity mitigation, GDP per capita was removed from the final model. Geographic distance was strongly negatively associated with viral spread (coefficient = −0.873, 95% HPD: −0.995 to −0.753), with decisive support for its inclusion in the model (activation rate = 1) (Fig. 9A). Chicken stock origin, defined as the number of annual chicken inventory at the source country, was consistently positively associated with virus spread (0.461, 0.111 to 0.845), while temperature origin (−0.484, −0.900 to −0.237) and temperature destination (−0.227, −0.499 to −0.047) was also considered as a negative and supportive predictor. Virus sample size, number of H7 sequences included per discrete location at both origin and destination locations, was positively associated with viral transmission. The estimated coefficient was 1.200 (0.777–1.600) for origin and 0.598 (0.402–0.809) for destination, both with full activation rate. The results incorporating GDP were similar (Supplementary Fig. 24). These findings demonstrate the utility of the GLM framework in identifying key drivers of H7 viruses spatial dissemination.

## Discussion

**Fig. 10:**
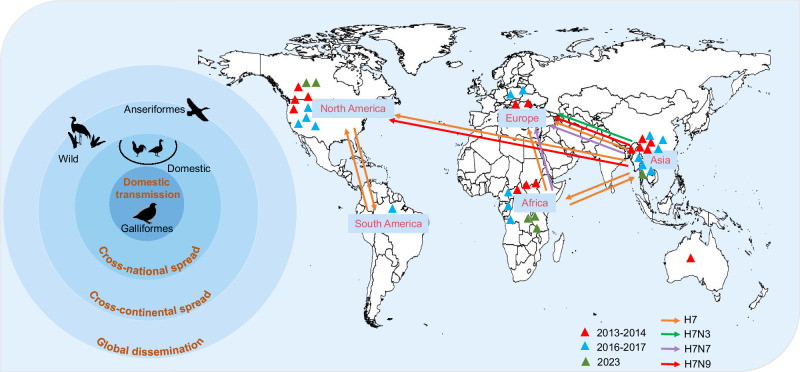
Graphical summary of the global transmission dynamics of H7 avian influenza virus. This figure illustrates key large-scale outbreaks of H7 viruses, including those in poultry, with major events marked in different colored triangles. The number of triangles on each continent corresponds to its ascending rank in outbreak frequency during the same period (e.g., one triangle represents lowest, two triangles represent intermediate, three triangles represent highest). The concentric circles on the left show the effect of different hosts on geographic spread. These colored arrows do not represent literal geographic paths but instead indicate directionality of intercontinental spread according to the result of Markov jumps. Credit: all silhouettes in Fig. 10 were obtained from PhyloPic and PhyloPic is under a Creative Commons CC0 license. Maps were generated using the R package rnaturalearth with data from Natural Earth (public domain, https://www.naturalearthdata.com).

This study employed integrative phylogenetic and phylogeographic analyses to unravel the evolutionary trajectories of H7 influenza viruses, with a focus on subtype-specific divergence in host-driven evolutionary dynamics and global dissemination patterns. The results revealed heterogeneous evolutionary dynamics and spatial diffusion rates across H7 subtypes, characterized by asymmetric intercontinental viral migration dominated by unidirectional gene flow from Asia and Europe to Africa and other continents, with sustained viral exports facilitated by Anseriformes and wild birds (Fig. 10). These data underscore the critical role of host-taxonomic partitioning in shaping H7 epidemiology, necessitating enhanced genomic surveillance targeting key avian-human interfaces under the One Health framework. Prioritizing real-time monitoring of viral mobility hotspots (e.g., East Asian flyways) and integrating spatial-explicit risk models could optimize preemptive control strategies against future transboundary spread.

Countries and regions are divided into continents based on their geographical location. This classification method not only helps to analyze the distribution and spread of viruses in different regions more systematically but also reveals the transmission dynamics and potential hotspot areas between regions. From the perspective of time distribution, the number of sequences collected in January each year has significantly increased, reflecting a certain seasonality in the occurrence of H7 avian influenza. This seasonal pattern may be related to the migration of wild birds in winter or an increase in poultry density^21^. Migratory birds may spread viruses to new areas during their winter migration, while poultry in intensive winter farming environments may promote rapid transmission and evolution of viruses^22^. This discovery emphasizes the importance of strengthening monitoring and prevention measures in the season when birds migrate. Despite widespread human outbreaks of H7N9, its declining genetic diversity reflects viral bottlenecks and effective controls, whereas H7N3’s rising diversity indicates sustained poultry circulation in the Americas despite minimal human impact. These findings highlight the need to interpret genetic diversity estimates in the context of surveillance coverage, lineage dynamics, and ecological settings.

The results of discrete trait analysis and Markov jumps consistently indicate that Asia is the main origin and sustained transmission center of H7 viruses global spread. The average duration of H7 viruses in Asia (42.63% Markov reward) is much higher than in other regions, and the transmission route from Asia to Europe is supported by the highest Bayesian factor. It may stem from the dense poultry farming system in Asia, frequent trade in live poultry, and the intersection of migratory routes for birds^23^. In addition, the long-term presence of H7N9 subtype in Asia may be attributed to its adaptive evolution. A key mechanism is the acquisition of mutations (e.g., Q226L, T160A, and G186V in *HA*) that shift receptor binding preference towards human-type α 2,6-sialic acid receptors from the avian-type α 2,3-sialic acid receptors^24,25^.

Although the cross-regional transmission of H7 viruses is mainly one-way (such as from Asia to Africa), its sustained circulation ability varies significantly across different continents. The H7N3 virus in North America has formed an independent gene pool, indicating that this subtype may have achieved long-term adaptive evolution through local migratory birds (such as American waterfowl) or poultry populations^26^. Additionally, the prevalence of H7N7 in Europe may be related to the high density and frequent virus mutation events in the local industrial poultry industry^27,28^. This regional adaptive differentiation suggests that once a virus is introduced into a new geographical area, it may form a unique evolutionary branch through gene mutation, thereby reducing the efficiency of cross-regional re-transmission.

This study reveals the temporal and spatial heterogeneity presented by H7 virus subtypes in the global transmission network through continuous phylogeography reconstructions and provides a new perspective for understanding the ecological-social coupling mechanism of H7 avian influenza dispersal. The trajectory of the H7 virus stepwise spreading from its East Asian origin to the west is significantly spatially and temporally coupled with the restructuring of the global poultry industry chain at the end of the 20th century^29^. In the past 50 years, global trade in live animals has also increased significantly, especially poultry and pigs^29^. The characteristics of poultry trade in Asia may have significantly contributed to the risk of emergence, spread, and persistence of avian influenza. A large fraction of poultry products is traded through live poultry markets in Asia, and these live poultry markets can be connected over large geographical distances through trade links, increasing the risk of avian influenza spread. Notably, the transmission of the virus between North America and Europe coincides with the migration routes of Pacific birds^30^ and the surge in international trade in frozen poultry meat in the Americas^31^, indicating prevention and control strategies for H7 viruses need to balance ecological monitoring and cross-border trade supervision to prevent the risk of virus spreading through dual pathways. Interestingly, the H7 virus is transmitted directly from Asia to South Africa, which is different from the previously discovered transmission route of H5N1, spreading from Asia to West Africa and then to South Africa^32^. West Africa has a wealth of extensive wetlands, such as the Senegal River delta, which serves as vital winter habitats for numerous migratory duck species^33^. Several reasons may explain the direct spread of the H7 virus from Asia to South Africa. Some migratory birds, such as shorebirds, fly directly from Asia to South Africa along the Indian Ocean route without passing through West Africa. The commercial poultry trade (the movements of cull chickens) between South Africa and Asia, such as breeding birds and feed, may directly introduce H7 viruses^34^. In addition, there are large numbers of free-range poultry in South Africa with weak biosafety measures, which are highly mobile and in constant contact with the outside world, making it easy for the virus to spread rapidly from one population to another^35^. Furthermore, although H7N9 has zoonotic potential^36^, the primary reason that H7N9 has not established a widespread global transmission geographically may be the implementation of live poultry market controls in China since 2013^37^.

There are significant differences between subtypes: the dispersal velocity of H7N3 and H7N7 is evidently higher than that of H7N9. This difference may be driven by several factors. First, H7N3 and H7N7 are widely spread in wild waterfowl, relying on the long-distance migration of migratory birds to achieve rapid spread^38,39^, while H7N9 relies more on poultry trade and local live poultry market networks^40^. Second, the *HA* gene of H7N9 has undergone multiple antigenic drifts such as T160A mutations after 2013^24^, which may reduce its transmission efficiency in wild birds. Third, China has implemented large-scale poultry vaccination against H7N9 since 2017^41^, which may have suppressed its spatial spread velocity.

The current results showed that the H7 virus stays significantly longer in Anseriformes (such as ducks) than in Galliformes (such as chickens), suggesting that Anseriformes may have provided a more stable ecological niche for its evolution. This is consistent with the classic view that waterfowl (most Anseriformes are waterfowl) are natural hosts of avian influenza virus and long-term infections are often asymptomatic, promoting the continuous circulation of the virus^42^. And long-term adaption within the host may enhance the potential for cross species transmission, possibly by increasing viral diversity, which favors rare advantageous mutations, and by selecting traits that pre-adapt the virus to new hosts, lowering the genetic barrier for cross-species transmission^43,44^. It is worth noting that the H7N7 and H7N3 subtypes exhibit opposite Markov jump directionality: The transmission frequency of H7N7 from Anseriformes to Galliformes is three times that of the reverse direction, while H7N3 tends to spread more from Galliformes to Anseriformes. This difference may be due to subtle variations in the proteolytic cleavage site in the HA protein between subtypes^45^. For example, low-pathogenic H7N7 avian influenza viruses naturally infect wild aquatic birds belonging to the Anseriformes order^14^, while low-pathogenic H7N3 virus transmitted in poultry and undergone adaptive mutations in the HA protein enhances binding to Galliformes α−2,3 receptor variants. This mutation may reduce its affinity for the primitive receptor of Anseriformes, resulting in a decrease in the efficiency of reverse transmission^46^.

This study also revealed the transmission characteristics of H7 subtype avian influenza in different avian hosts (Anseriformes and Galliformes, wild birds and domestic birds) by phylodynamics incorporating geography and host. The dispersal velocity of viruses in Anseriformes is significantly faster than that in Galliformes, which is directly related to the frequent migration of Anseriformes while Galliformes are typically non-migratory^47^. Further research has found that wild birds contribute more to the transmission of H7N3 and H7N9, while domestic birds contribute to H7N7 transmission. This may reflect the local outbreak of H7N7 in poultry spreading through trade networks (such as live poultry transportation)^48^. Our refined analysis of host interface combinations also offers important insights into the mechanisms of H7 viruses spread. The markedly high velocity observed in Anseriformes-wild populations may be likely attributable to the long-distance migratory behavior of wild waterfowl, which function as efficient natural vectors for viral dispersal across continents^14^. The intermediate velocity of Anseriformes-domestic populations likely reflects the role of domestic duck farming and trade, which facilitates regional spread but at a slower pace than natural migration. In contrast, the lower velocities among Galliformes-domestic and particularly Galliformes-wild populations may stem from the sedentary nature of many Galliformes species such as pheasants in confined habitats and the absence of strong anthropogenic drivers like trade networks.

The differential dispersal velocities between HPAI and LPAI provided critical implications for understanding H7 transmission. The markedly slower dispersal velocity of HPAI viruses in Galliformes, especially H7N3-HPAI, suggests that despite their severe impact, their geographic transmission may be limited within poultry populations due to high host mortality. Conversely, the rapid transmission of LPAI viruses in Anseriformes underscored their role as a silent reservoir for long-range dissemination, consistent with traditional conclusion^14^. The notable exception of H7N7-HPAI and H7N9-HPAI, which transmitted rapidly, highlighted that subtype-specific virological properties and probable local epidemiological factors can sometimes override the general trend of pathogenicity-defined transmission, warranting further investigation. One possible reason is that the emergence of an HPAI virus triggers an immediate and intensive surveillance response^7^, heightening surveillance likely leads to much quicker detection and reporting of more cases. While the virus may not actually be moving faster, the interval between infection and detection is dramatically shortened, creating a surveillance artifact that inflates the estimated velocity in our phylogeographic models. Besides, HPAI H7N7 and H7N9 outbreaks may have occurred in regions with extremely high host density and could transmit could spread rapidly between susceptible farms before control measures took full effect.

The results of wavefront distance showed that Anseriformes contributed more to the geographical expansion of H7 in recent years, which may be related to the migration of migratory birds caused. For example, AIVs are presumably co-evolved with migratory water birds, with the virus also persisting outside the host in subarctic water bodies and the warming of the Arctic has led to the northward expansion of waterfowl breeding grounds and increased contact with domestic poultry^49^. Besides, the sustained and widespread spread of H7 viruses in Anseriformes may be related to its stable maintenance in wild duck populations^50^, while the shorter wavefront distance in Galliformes suggests that its spread relies more on local outbreaks^26^. Human infections with AIVs have been uncommon^51^. However, H7N9 caused at least five waves in China during February 2013 and September 2017, with a total of 1564 human infections^4^. In the current study, the weighted diffusion coefficient was much greater than that of Galliformes, which may reflect human-mediated activities, such as trade or travel, accelerating the geographic spread of the virus rather than humans as hosts themselves^52^. While humans are dead-end hosts for most H7 subtypes, human activities such as the movement of poultry and poultry products acted as the key driver for the intercontinental dispersal of the virus.

This study represents the application of the GLM approach to the H7 AIVs, offering a perspective on its transmission dynamics. Geographic distance is identified as a consistently significant factor influencing migration rates, demonstrating that intercontinental transmission intensity decreases with increasing distance, consistent with spatial diffusion constraints. This discovery is consistent with previous research on H9N2 subtype^53^. Chicken stock origin consistently made a positive contribution to the spread of the virus, while temperature origin was considered as a supportively negative predictor. Environmental factors that may influence transmission include the stocking density, temperature, and airflow^54^. It was estimated 2–22 °C a risky window for human H7N9 infection in previous study^55^. Influenza viruses degrade more quickly in warm or dry conditions^56^, so high temperature may reduce viral persistence in the environment, which explains the negative correlation between temperature and transmission risk. Besides, many supplied birds arrive already exposed to AIVs, resulting in many broiler chickens entering the market as infected^57^, probably causing the virus to remain in the chicken. And sample size was also an important factor. GLMs that include and do not include sample size predictors were both conducted to examine whether the sample size may mask the effects of other variables. When including sample size in the model, the economically developed regions may have stronger prevention and control capabilities including health facilities and quarantine measures and significantly reduce the risk of transmission^58^.

These findings suggest that we can focus on the following aspects for the prevention and control of H7 viruses: First, enhance surveillance in regions with small sample sizes, particularly in areas with dense poultry farming and along migratory bird routes, to enable early detection and containment of virus transmission. Integrating real-time environmental variables such as temperature monitoring into early warning systems is important, particularly in regions with high poultry-human contact. Timely interventions such as market closures or poultry vaccination campaigns are crucial during the periods of climatically favorable viral transmission. Second, optimize resource allocation by prioritizing the prevention and control efforts in the virus origin areas and their surrounding regions to reduce the risk of local spread. Finally, improve monitoring capabilities in low-income regions through international cooperation to minimize the impact of sampling bias on the assessment of virus transmission.

This study is subject to certain limitations. The study relied on publicly accessible genetic sequences, which carry intrinsic biases in host and geographic representation. For instance, active monitoring of wild birds is infrequently conducted in some regions of Africa, probably compromising the reliability of metrics assessing wild bird-driven viral spread in these endemic areas. To counteract this limitation, our analytical approach employed different down-sampled protocols that standardized sample quantities across host categories and geographic clusters during subtype-specific analyses, aiming to reduce dataset imbalances. And the comparison of GLMs that include and do not include sample size predictors aimed to outline the impact of sampling bias within this dataset. Second, the bird migration networks in our analyses are binary, but the migration routes are heterogeneous between different wild bird species. Future research can better understand the transmission mechanisms of the virus with more detailed bird tracking data on movements. Finally, For the H7N9 subtype, the high number of inferred Markov jumps from humans to Galliformes should be interpreted with caution. This pattern could be artificially inflated by the substantial oversampling of human cases compared to poultry, which may mask the true direction of transmission, which is likely predominantly from poultry to humans.

In conclusion, this study provides an analysis of the spread, evolution, and host transmission dynamics of H7 avian influenza viruses. Through phylogenetic and spatial modeling, we reveal distinct lineage diversification patterns and identify key ecological and human-driven factors influencing cross-species transmission. Our findings highlight how geographic distance, temperature, GDP, and poultry density collectively shape viral dispersal across regions. By integrating genetic, spatial, and host data, this study advances understanding of the evolution and pandemic potential of H7 viruses. To mitigate the risk of cross-species transmission and global spread of H7 viruses, the study highlights the need for One Health-oriented strategies grounded in multisectoral collaboration and interdisciplinary integration, aimed at strengthening genomic surveillance and transboundary data sharing, with particular attention to Anseriformes on the migration routes and high-risk regions such as Asia and Africa that are severely affected by avian influenza.

## Methods

### Avian influenza H7 virus outbreak data

All reported and confirmed outbreaks of H7 viruses across various animal types in global were obtained from EMPRES-i+ Global Animal Disease Information System, Food and Agriculture Organization (FAO) (https://empres-i.apps.fao.org/) and World Animal Health Information System, WOAH. The detailed analytic pipeline was shown in Supplementary Fig. 25.

The analysis was based on publicly available datasets and did not include any direct interaction with or collection from human or animal subjects, and therefore ethics approval was not required for this research.

### Sequence curation and subsampling

We retrieved H7 genomic sequences (*HA* gene segment) and associated metadata (collection date, location, host species) from the NCBI Influenza Virus Resource (https://www.ncbi.nlm.nih.gov/genomes/FLU/; *n* = 1794) and GISAID (https://platform.epicov.org/; *n* = 6075) in November 2024. After removing duplicate, laboratory-derived and mixed-subtype isolates, 4351 sequences were obtained. To mitigate spatiotemporal sampling bias, we subsampled datasets by country and collection year (maximum 50 sequences/country), yielding 814 *HA* sequences for analysis. The date of each sequence was converted to a decimal format using BioAider v1.5.2.^59^. Furthermore, sequences were categorized into three subtypes (H7Nx) based on annotated lineage data.

To evaluate the robustness of the analysis and to reduce sampling bias, three distinct datasets were compiled for each subtype, and the number of sequences in each dataset is equivalent. Therefore, to facilitate sensitivity analysis, three different subsampling strategies were employed: (1) an epidemiology-based dataset, based on virus epidemiological information (sampling by location and collection date); (2) a tree-based dataset, based on phylogenetic diversity; and (3) a random dataset, randomly downsampling sequences. The tree-based dataset was employed in discrete trait phylogeographical analysis using the Phylogenetic Diversity Analyzer tool. Each dataset was listed in Supplementary Data. The results of this sensitivity analysis are detailed in Supplementary Table 2.

### Phylogenetic analysis

The sequences were aligned using the Multiple Alignment using Fast Fourier Transform (MAFFT v.7.526)^60^, checked using AliView v1.28^61^ and trimmed using trimAL v.1.4^62^ with a 50% gap threshold and further manually trimmed to obtain the open-reading frame. The best-fit nucleotide substitution model was selected using IQ-TREE v.2.0.7^63^. To ensure the presence of phylogenetic temporal structure, a scatterplot of root-to-tip genetic divergence against sampling date was created with TreeTime v.0.11.3^64^. The molecular clock outliers i.e., sequences that have disproportionately too much or too little root-to-tip divergence for their sampling time were deleted before the phylogenetic analyses. The specific filter method can be found at https://github.com/neherlab/treetime. Specifically, the method first calculates the residual for each terminal node using the formula: Residual = The distance from the root of the node to the node − (clock_rate * mean_date_constraint) – intercept, representing the difference between the observed and expected evolutionary distances. Then the algorithm computes the interquartile distance (IQD) of all residuals. Sequences are flagged as outliers if their absolute residual exceeds 3 * IQD. This robust statistical approach, based on the IQD, ensures that the threshold remains unaffected by extreme values, thereby maintaining the reliability of outlier identification in molecular clock analysis.

### Bayesian evolutionary inference

For estimating the divergence times and evolutionary rates of the H7 virus and three subtypes, an uncorrelated relaxed clock model under a Bayesian framework using Markov chain Monte Carlo (MCMC) sampling in BEAST v.1.10.4 with the Broad-platform Evolutionary Analysis General Likelihood Evaluator (BEAGLE) high-performance library was used^64,65^. Phylogenies were reconstructed for H7 using a General Time Reversible nucleotide substitution model with a gamma distribution of substitution rates, a Bayesian Skygrid coalescent model and an uncorrelated lognormal clock.

MCMC chains were performed for 50–200 million states, and this process was conducted on at least two separate occasions, sampling every 5000–20,000 and discarding 10% as burn-in. Runs were combined to ensure an effective sample size (ESS) of more than 200 in Tracer v.1.7.2^65^, and the statistical uncertainty showed in values of the 95% highest posterior density (95% HPD). MCC trees for each subtype were compiled using TreeAnnotator v1.10.4 following the removal of a suitable burn-in^66^. Subsequently, the trees were visualized using FigTree v1.4.4 and Chiplot^67,68^.

Changes in relative genetic diversity for the H7 and three *HA* subtypes were analyzed using the uncorrelated lognormal relaxed-clock with Skygrid coalescent model^69^, with the need to provide the age of the youngest tip. Skygrid improves upon the Skyride model by adjustments to the effective population size at specific and user-determined intervals in real-time. It also provides a more accurate estimation of root heights and supports analysis using data from multiple locations. The resulting visualizations depict fluctuations in the Ne of virus across different time periods, with a time interval of 5 years by default settings, and can be analyzed and visualized by Tracer version 1.7.2.

### Bayesian discrete phylogeographic and host-based diffusion analysis

To estimate spatial diffusion dynamics among a set of 5 geographic regions (Africa, Asia, Europe, North America, and South America), a Bayesian discrete phylogeographic approach was employed^70^. This method is based on the geographic locations recorded at the tips of the empirical *HA* phylogenies, modeling the transition history among these locations as a continuous time Markov chain (CTMC) process. This approach allows for the inference of undetected locations at ancestral nodes within each tree of the posterior distribution. Symmetric discrete-trait phylogeographic analyses (*i.e*., reversible CTMC model) with BSSVS were conducted in BEAST v.1.10.4 to determine a sparse set of transition rates that fully summarizes the epidemiological connectivity. In addition to these analyses, an inference of the complete Markov jump history over time was included to enable the quantification of state transitions and the duration spent in specific states across each phylogenetic branch^71^. The number of jumps between locations was presented in maps. Bayes factors, which represent the statistical significance of the transition rates, were evaluated using SpreadD3 v.0.9.7^72^.

In addition to the geographic analysis, a Bayesian discrete phylogeographic approach was also applied to investigate the transmission dynamics of H7 and its subtypes across different host groups. Hosts were divided into the following categories: bird, human, environment and others. Because of the large number of bird hosts, the bird was further divided into Anseriformes and Galliformes^73^. Furthermore, based on the sequence names and host information, the bird hosts were categorized into domestic and wild groups, according to the following criteria: (1) host species explicitly designated as “poultry” or “farm” in sequence metadata (such as “chicken”, “domestic duck”) and host species known to be primarily commercially reared (e.g., Gallus gallus domesticus) are considered domestic; (2) host species typically considered wild reservoirs (e.g., ‘mallard’, ‘teal’, ‘wild bird’) were classified as wild. Phylogenetic diffusion model serves as versatile frameworks for trait evolution and is not limited to spatial data^74^. Given the flexibility, the transmission of H7 and subtypes across different hosts throughout its evolutionary history was investigated. To reconstruct spatial diffusion among different hosts, the number of Markov jumps and Markov reward were also calculated similar with the above analysis of discrete geography.

### Continuous phylogeographic analysis

To complement the discrete spatial diffusion analysis and acquire a more detailed geographic history, the H7 and subtypes (with each major phylogenetic lineage analyzed separately to estimate lineage-specific spread velocity) diffusion dynamics in continuous space (latitudinal and longitudinal data at the country level) were estimated using Brownian motion random walk model^75^ with 0.01 jitter window size. The spatiotemporal realizations generated by the continuous diffusion process enable to obtain various statistics related to spatial epidemic dynamics, eliminating the need to consider autocorrelation, which is typically a requirement in mathematical models used in spatial ecology^76^.

In this study, three statistics of dispersal dynamics were calculated, either for H7 or for its subtypes, including the dispersal velocity (km/year), diffusion coefficient (km^2^/day), and wavefront distance (km). Diffusion coefficient (*D*) was also estimated, this measure can be estimated using Eq. (1):

1 $${D}_{{{\rm{original}}}}=\frac{1}{n}{\sum }_{i=1}^{n}\frac{{d}_{i}^{4}}{{t}_{i}^{2}}$$

where *d*_i_ and *t*_i_ are the geographic distance travelled (great-circle distance measured in kilometers) and the time elapsed (measured in years) on each phylogeny branch, respectively. However, according to previous research^77^, the weighted coefficient *D*_weighted_ using Eq. (2) defined by Trovão et al.^78^ allows better discrimination among epidemics with different diffusivity because it is associated with a smaller variance.

2 $${D}_{{{\rm{weighted}}}}=\frac{{\sum }_{i=1}^{n}{d}_{i}^{4}}{{\sum }_{i=1}^{n}{t}_{i}^{2}}$$

The mean branch dispersal velocity vbranch (*v*_branch_) was also estimated using the spatio-temporal information extracted from posterior trees using the spreadStatistics function as Eq. (3).

3 $${v}_{{{\rm{branch}}}}=\frac{1}{n}{\sum }_{i=1}^{n}\frac{{d}_{i}}{{t}_{i}}$$

As with the diffusion coefficient, we also calculate the weighted branch dispersal velocity (*v*_weighted_) as Eq. (4):

4 $${v}_{{{\rm{weighted}}}}=\frac{{\sum }_{i=1}^{n}{d}_{i}}{{\sum }_{i=1}^{n}{t}_{i}}$$

For a given tree, branches with short duration will have respectively less of an impact on *v*_weighted_ and *D*_weighted_ than on *v*_branch_ and *D*_original_, and therefore also on the resulting variance among *v*_weighted_ and *D*_weighted_ values across all trees.

In addition to these lineage dispersal statistics, spatial wavefront distance was also estimated, which captures the largest great-circle distance from each phylogenetic lineage to the estimated epidemic origin at a given time^76^. In addition, 1000 phylogenetic trees sampled from the post-burn-in posterior distribution of trees inferred for the data set and a maximum consensus tree file estimated from these 1000 sampled trees were used to plot the dispersal history of H7 viral lineages globally. The results of the continuous phylogeographic analysis were visualized in the R package ‘seraphim’ and ‘diagram’^77^.

### Phylodynamics incorporating geography and host

Furthermore, to help clarify and compare the role of different host populations (Anseriformes vs. Galliformes and wild vs. domestic) in the expansion dynamics of H7 viruses and subtypes (with each major phylogenetic lineage analyzed separately to estimate lineage-specific spread velocity), the process of continuous spatial diffusion incorporating a discrete host transmission process was performed in a single Bayesian analysis^78^. This modeling was conducted separately for each of the three subtypes. While the discrete (host) and continuous (geography) aspects of the process were treated separately, the joint inference facilitates the assessment of the host-specific diffusion patterns. Given that H7 viruses can be categorized into high-pathogenicity and low-pathogenicity strains, we further analyzed their respective dispersal velocities across different avian host species. Comparison of virus dispersal velocity among different hosts was tested by Mann–Whitney U test.

### Phylogeographic inference with epidemiological predictors

The distributions of empirical trees were also employed to carry out a phylogeographic reconstruction within the context of a GLM framework to recognize the risk factors that promote the spread of H7 in 22 countries worldwide. To identify the epidemiological, we first compiled and processed various datasets relevant to viral diffusion. The potential predictors contributing to the transition between locations were considered in the discrete phylogenetic framework, which included (1) geographical distance, (2) air transportation data, (3) bird migration, (4) live poultry trade, (5) chicken stock, (6) GDP per capita, (7) annual mean temperature, (8) annual precipitation. To test whether geographical proximity predicts viral diffusion, the distance between locations based on latitude and longitude was calculated using Haversine Formula^79^. Air transportation data (monthly volume of airline passengers) were collected from Worldpop Hub^80^. Four countries were missing these data, and we replaced it with data from countries with similar GDP. Information on bird migration^30^, GDP per capita^81^, temperature, and precipitation^82^ was obtained from previous literature. The live poultry trade data are available on the United Nations Comtrade Database^83^. Air transportation data, chicken stock, GDP per capita, temperature, and precipitation were added to the model as covariates for both origin and destination. All predictors were log-transformed and standardized except bird migration before inclusion in the matrices. Finally, to examine the effect of virus sample size (number of H7 sequences included per discrete location) on genetic data, origin and destination sample sizes were included or excluded as a predictor in our GLM models, respectively. Prior to GLM modeling, Spearman rank correlation analysis was performed among candidate predictors. Variables exhibiting |r| > 0.7 were flagged for potential collinearity. Subsequently, variance inflation factors (VIF) were calculated to identify significant multicollinearity.

This GLM approach was extended from the discrete diffusion model, which characterizes the logarithm of the transition rates within the CTMC matrix as a log-linear function dependent on multiple potential indicators of viral spread. It incorporates a variable number of potential predictors, denoted as *P*, represented by the vector X = (x_1_,…, x_P_). Each predictor x_p_ is a one-dimensional array of values associated with the transition rates from state i to state j in the rate matrix, formatted as *x*_p_ = (x_1,2,p_,…x_K−1,K,p_)′. The GLM treats each immediate transition rate Λ_ij_ for i ≠ j in *Λ* as a log-linear function based on the set of predictors *X* using Eq. (5):

5 $$\log {\Lambda }_{{{\rm{ij}}}}={\beta }_{1}{\delta }_{1}{x}_{{{\rm{i}}},{{\rm{j}}},1}+{\beta }_{2}{\delta }_{2}{x}_{{{\rm{i}}},{{\rm{j}}},2}...+{\beta }_{P}{\delta }_{P}{x}_{i,j,P}$$

where (*β*_1_, *β*_2_,…, *β*_*P*_)′ represent the effective sizes for the predictors, quantifying their contribution to matrix Λ, and (δ_1_, δ_2_,..,δ_*P*_) are variables that determine whether each of the *P* predictors is included or excluded in the model.

In interpreting the GLM results, the following criteria were applied to assess the importance and significance of predictors:

Activation rate (the frequency with which a predictor is included in the model) was used to gauge importance: an activation rate ≥50% indicates a highly important predictor, 10–50% indicates moderate importance, and <5% suggests potential unimportance.

HPD intervals (Highest Posterior Density intervals) from the posterior distribution were examined for each coefficient: if the HPD interval does not contain zero, the effect is considered significant; if it contains zero, the effect is uncertain.

Coefficient magnitude was classified as: |coefficient|> 0.5 indicates a strong effect, 0.2 <|coefficient|< 0.5 indicates a moderate effect, and |coefficient|< 0.2 indicates a weak effect.

These standards ensure a structured evaluation of the predictors’ roles in viral spread dynamics.

### Reporting summary

Further information on research design is available in the Nature Portfolio Reporting Summary linked to this article.

## Supplementary information

Supplementary Information

Peer Review file

Reporting Summary

## Source data

Source Data

## Data Availability

Outbreaks in birds worldwide were obtained from World Animal Heath ihformation system (WAHIS), World Organisation for Animal Heath (https://wahis.woah.org/), and EMPREs-i+ Global Animal isease information System, Food and Agricuture Organisation (https://empres-i.apps.fao.org). All reported and confirmed infections of the H7 virus in humans were obtained from WHO (httos://www.who.int/emergencies/disease-outbreak-news). The curated datasets generated in this study, including subsampled H7 *HA* alignments, associated metadata, BEAST XML input files, posterior tree distributions and GLM predictor matrices have been deposited in https://github.com/skylynf/H7_Dynamics. A comprehensive list of GenBank and GISAID accession identifiers is provided in the data/Supplementary Data.xlsx file of the GitHub repository (https://github.com/skylynf/H7_Dynamics), and archived at Zenodo with the 10.5281/zenodo.19239968. Source data are provided with this paper.

## Code availability

The reproducible analysis pipeline, implemented as a workflow together with the underlying Python and R scripts, including Bayesian analysis and visualization with full documentation, is available at Github Repository (https://github.com/skylynf/H7_Dynamics), and archived at Zenodo with the 10.5281/zenodo.19239968.
