# Supplementary material for: Disentangling the drivers and host-mediated global spread of H7 influenza A virus
Source: Nat Commun. 2026 May 6;17:6076. doi: 10.1038/s41467-026-72718-9 (PMC13351079; doi:10.1038/s41467-026-72718-9)
Supplement: Supplementary file 1 — Supplementary Information [file 41467_2026_72718_MOESM1_ESM.pdf]

Disentangling the Drivers and Host-Mediated Global Spread of H7 Influenza A Virus

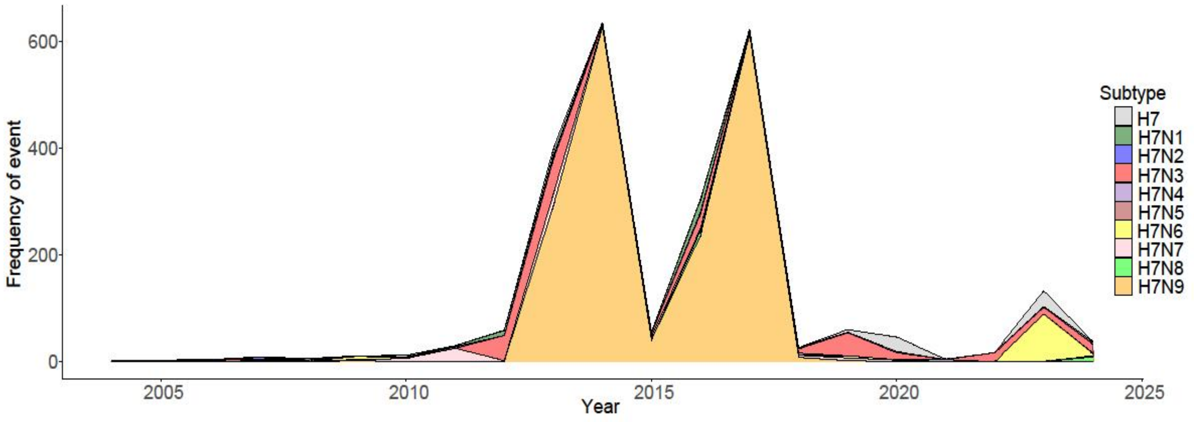

Supplementary Fig. 1. Temporal changes in H7Nx subtype prevalence estimated using observation dates of all reported cases submitted to the FAO from January 2000 to November 2024.

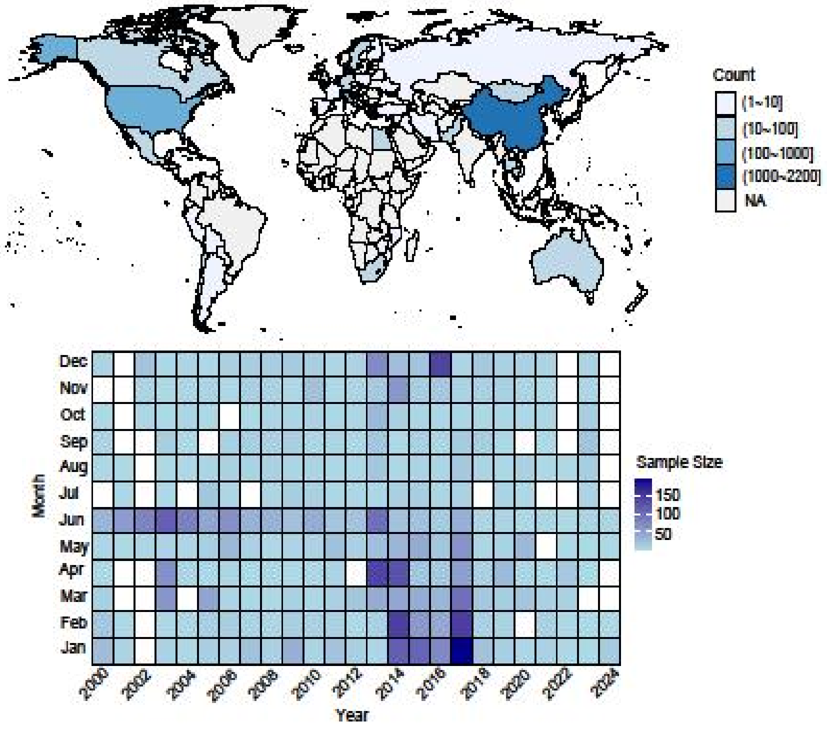

Supplementary Fig. 2. Distribution of sequences submitted to the GISAID and NCBI Influenza Virus Resource databases by region (top) and by month (bottom). Maps were generated using the R package rnaturalearth with data from Natural Earth (public domain, https://www.naturalearthdata.com).

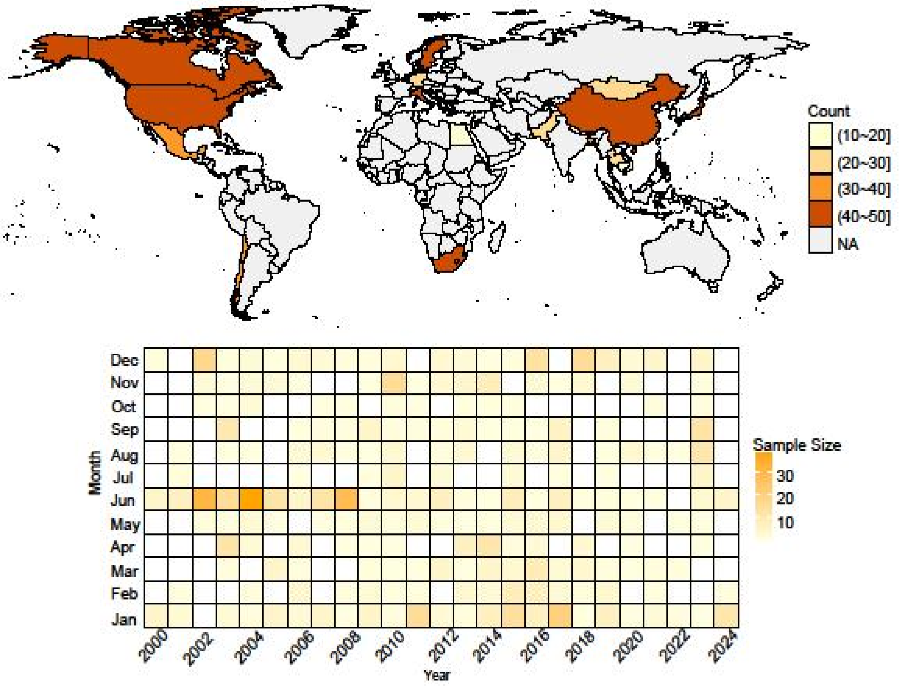

Supplementary Fig. 3. Distribution of sampled sequences submitted to the GISAID and NCBI Influenza Virus Resource databases by region (top) and by month (bottom). Maps were generated using the R package rnaturalearth with data from Natural Earth (public domain, https://www.naturalearthdata.com).

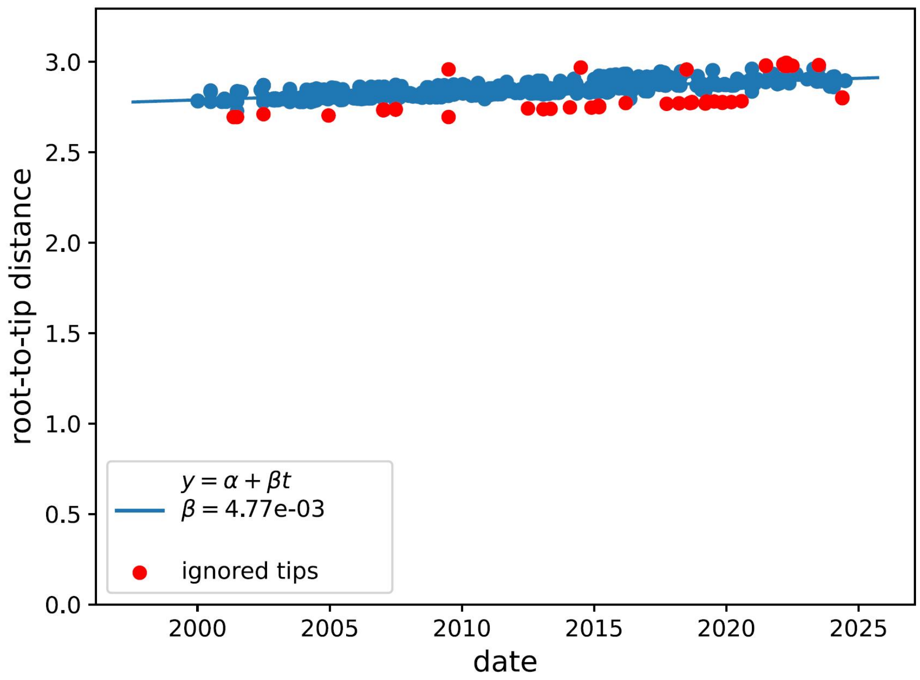

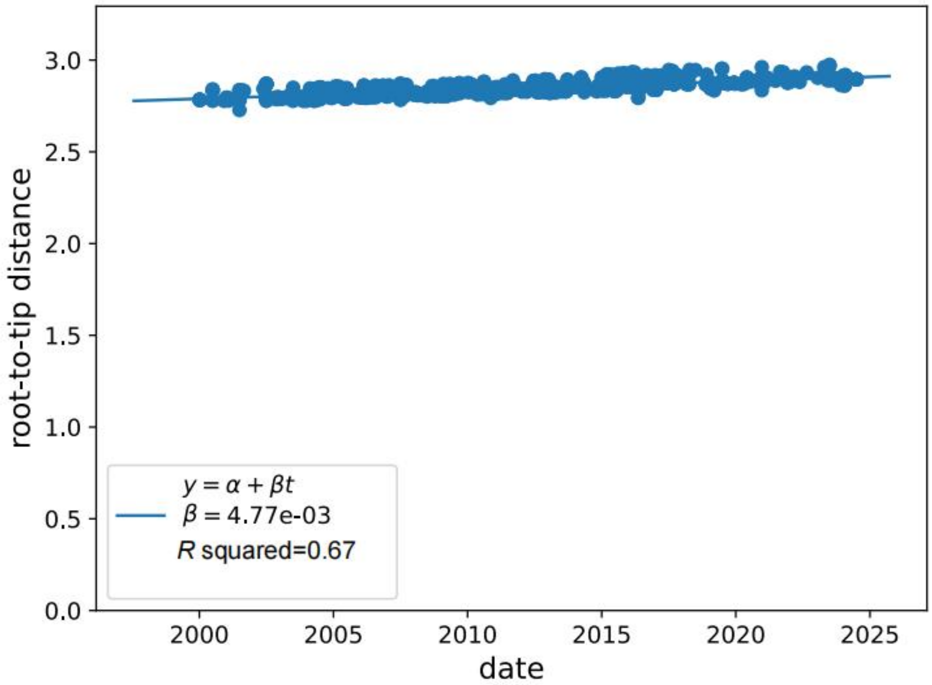

Supplementary Fig. 4. Strong temporal signal tested in Treetime of HA genes of H7 (R squared=0.67). The initial result of Treetime (Top). The red dots represent that this sequence does not have a time structure, and there are a total of 58 sequences that need to be excluded (Bottom).

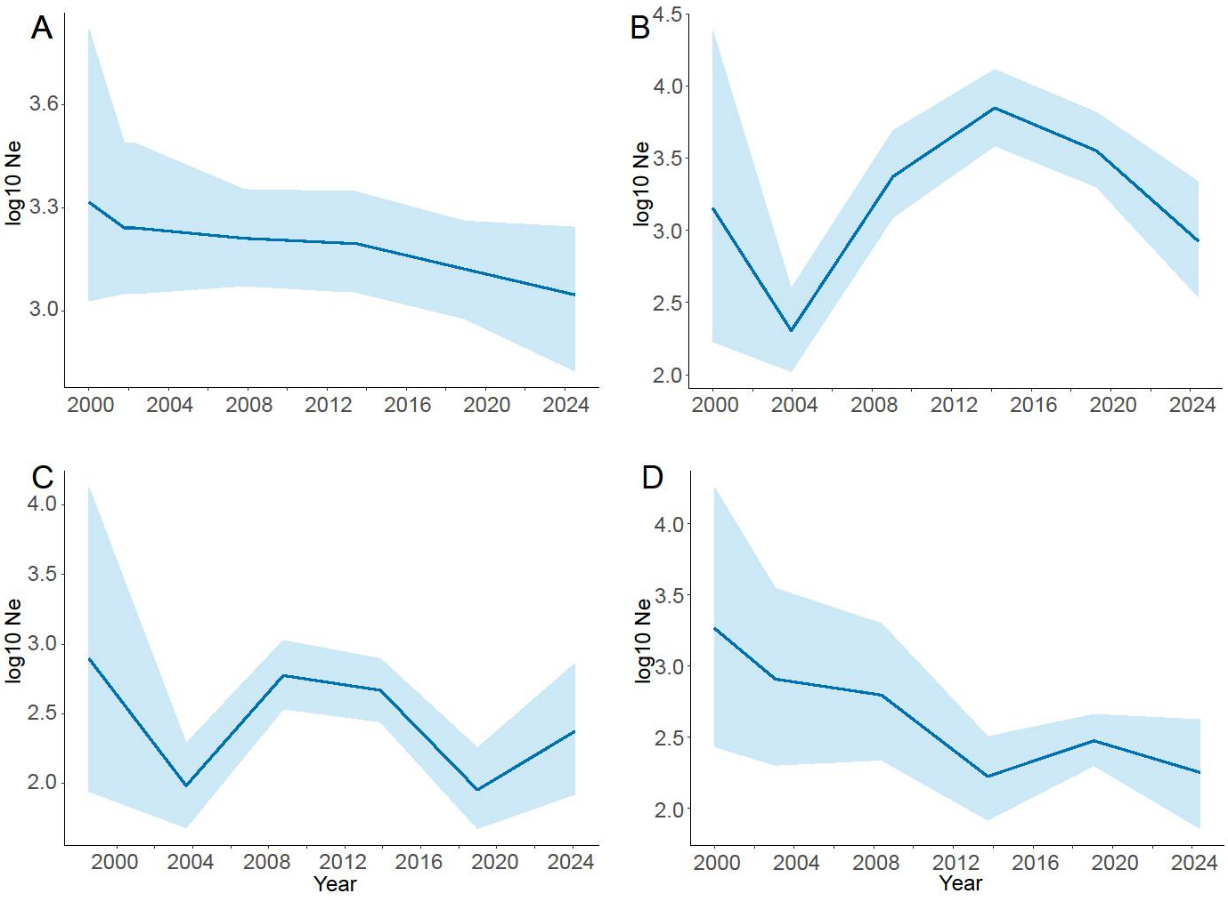

Supplementary Fig. 5. The solid (blue) line represents the posterior median of the population size over time, while the upper and lower lines indicate the 95% highest posterior density (HPD) interval. H7 (A), H7N3 (B), H7N7 (C), and H7N9 (D).

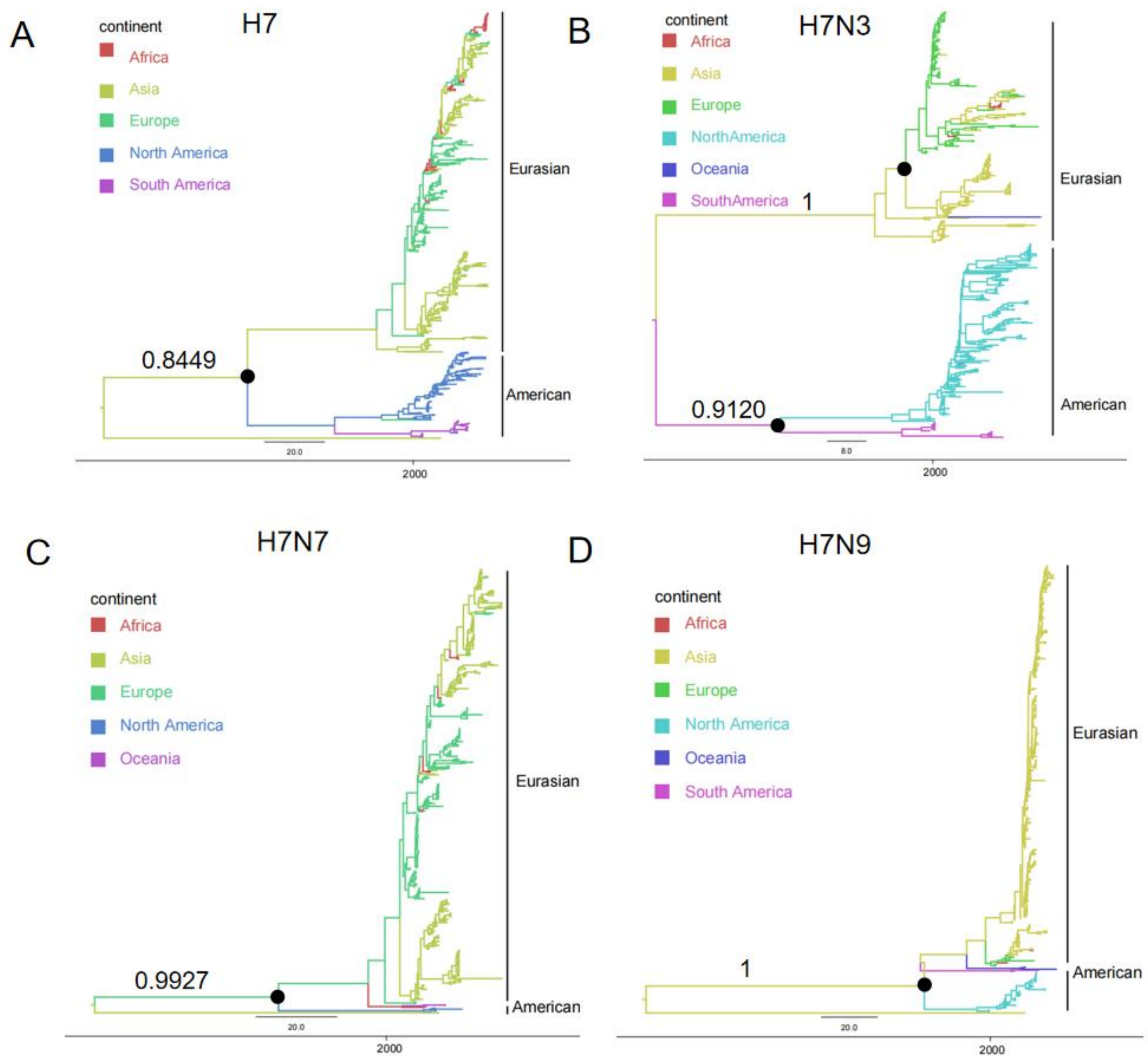

Supplementary Fig. 6. Time-scaled maximum clade credibility (MCC) trees of H7 HA genes sequences plotted by FigTree software, with branch colors representing geographic information. The number on the branch represents the posterior probability. H7 (A), H7N3 (B), H7N7 (C), and H7N9 (D).

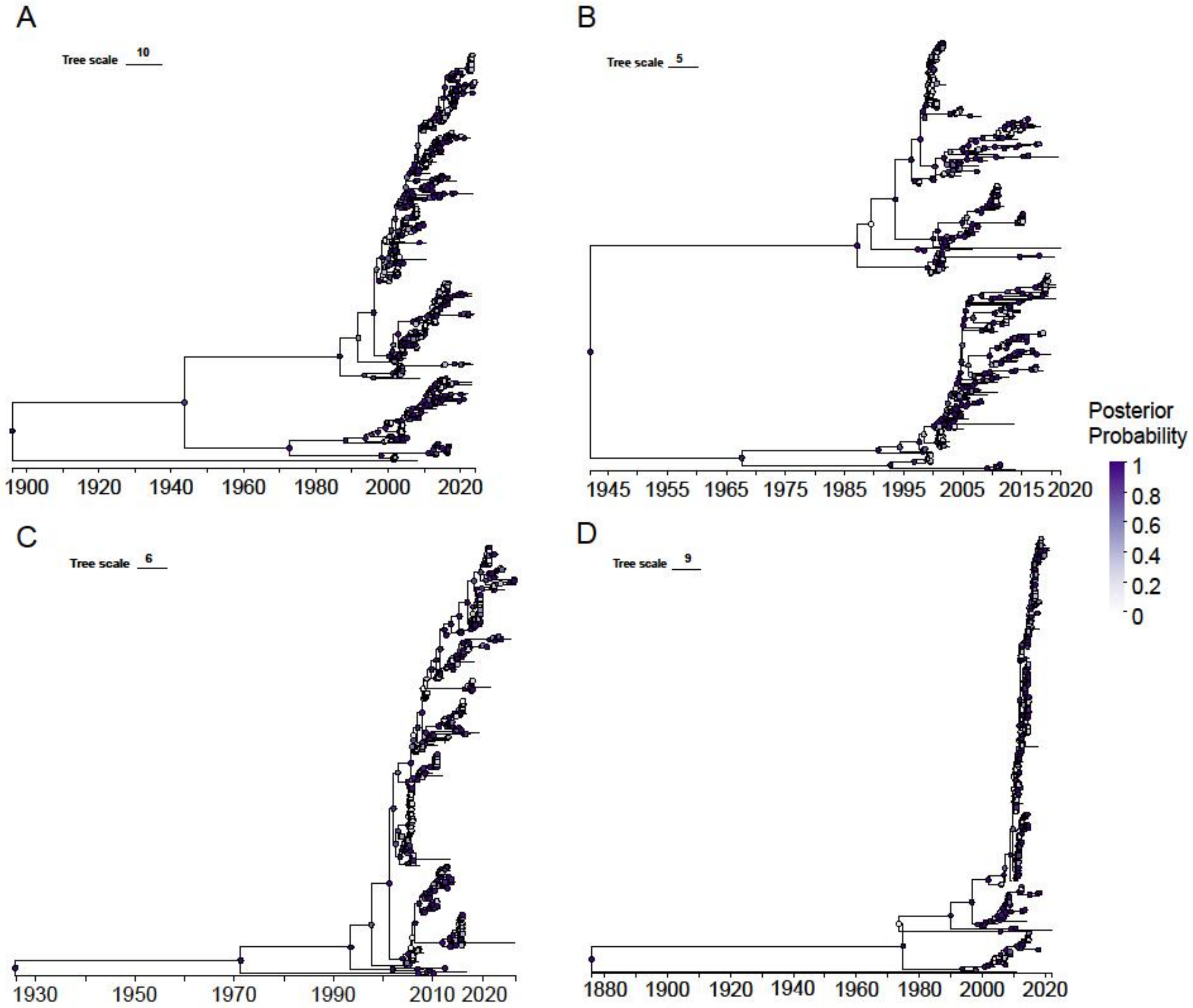

Supplementary Fig. 7. Time-scaled maximum clade credibility (MCC) trees derived using Bayesian phylogenetic analysis, where the support rate of nodes is represented by posterior probability. Nodes with a posterior probability greater than 0.95 are considered highly reliable, while nodes below 0.75 have weaker support. H7 (A), H7N3 (B), H7N7 (C), and H7N9 (D).

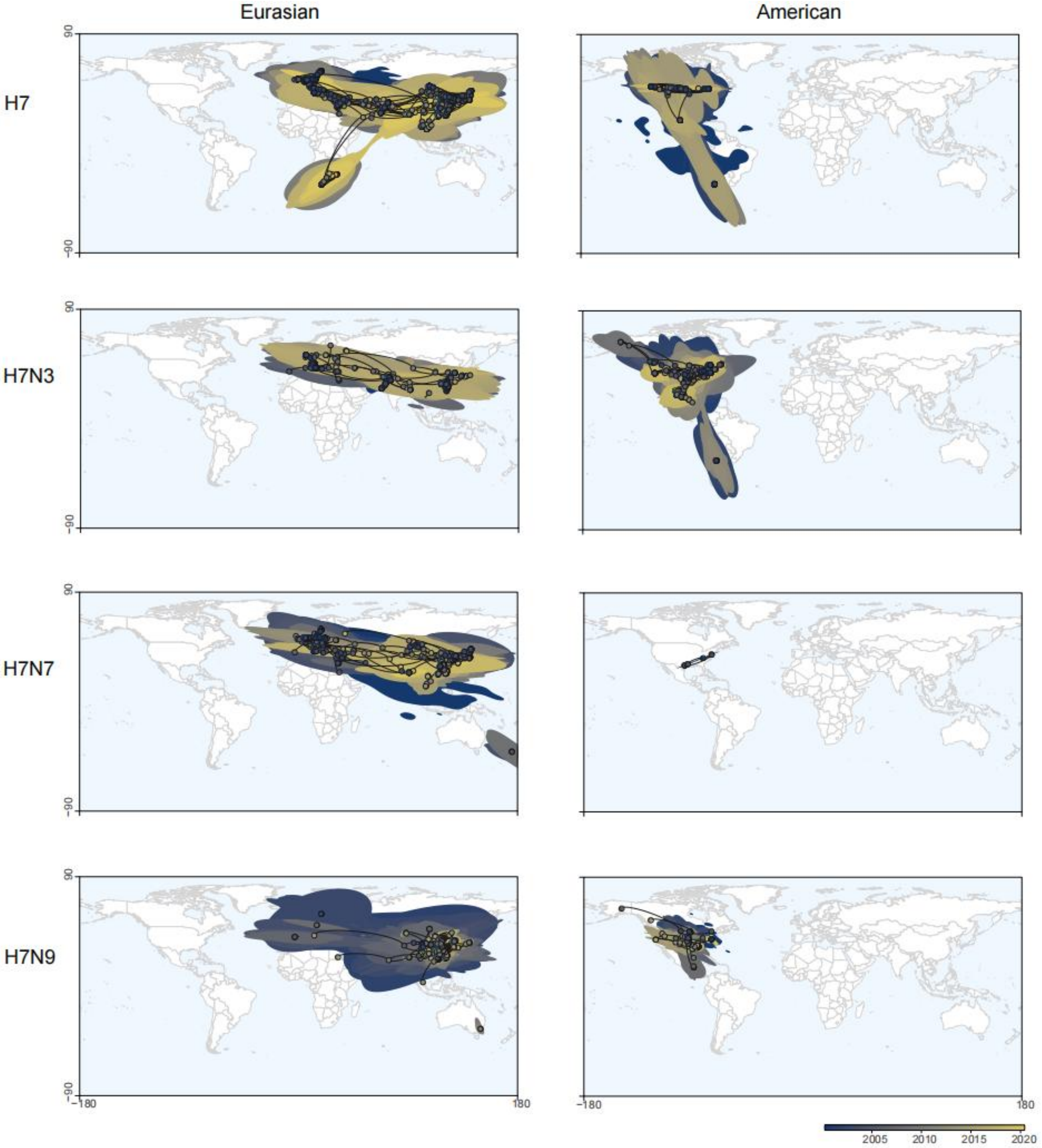

Supplementary Fig. 8. Continuous phylogeographic reconstruction of the spread of H7 and subtypes, with an interval of 80% HPD is depicted by shaded areas, illustrating the uncertainty of the phylogeographic estimates. The column on the left represent the Eurasian lineage, while the column on the right represent the American lineage. Maps were generated using the R package rnaturalearth with data from Natural Earth (public domain, https://www.naturalearthdata.com).

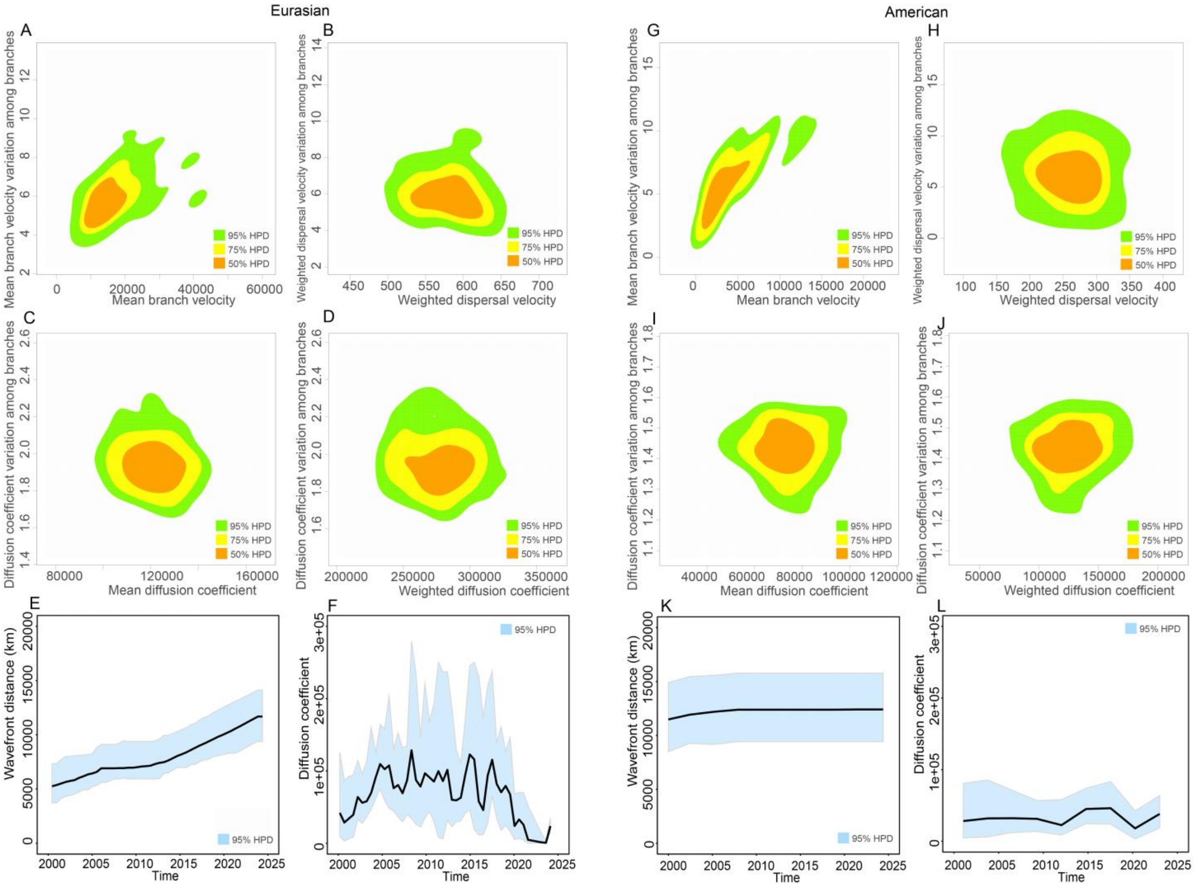

Supplementary Fig. 9. Estimated dispersal statistics for H7N3.

The two columns on the left represent the Eurasian lineage, while the two columns on the right represent the American lineage. A and G, kernel density estimates of mean branch dispersal velocity parameters. B and H, kernel density estimates of weighted branch dispersal velocity parameters. C and I, kernel density estimates of original diffusion coefficient parameters. D and J, kernel density estimates of weighted diffusion coefficient parameters. E and K, furthest extent of epidemic wavefront (spatial distance from epidemic origin). F and L, Evolution of weighted diffusion coefficient. For panels E, F, K, and L, solid lines represent the posterior median values of wavefront distance and diffusion coefficient, and shaded areas denote the 95% HPD intervals.

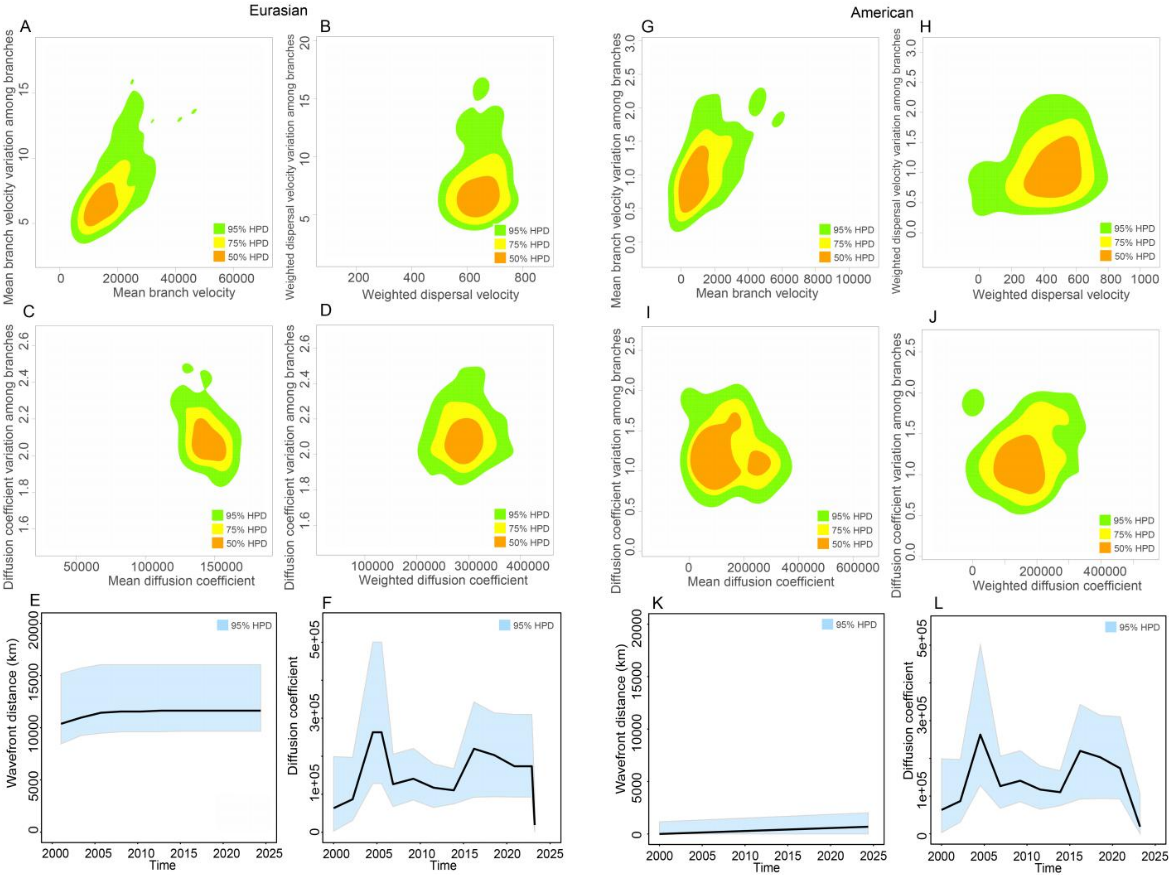

Supplementary Fig. 10. Estimated dispersal statistics for H7N7.

The two columns on the left represent the Eurasian lineage, while the two columns on the right represent the American lineage. A and G, kernel density estimates of mean branch dispersal velocity parameters. B and H, kernel density estimates of weighted branch dispersal velocity parameters. C and I, kernel density estimates of original diffusion coefficient parameters. D and J, kernel density estimates of weighted diffusion coefficient parameters. E and K, furthest extent of epidemic wavefront (spatial distance from epidemic origin). F and L, Evolution of weighted diffusion coefficient. For panels E, F, K, and L, solid lines represent the posterior median values of wavefront distance and diffusion coefficient, and shaded areas denote the 95% HPD intervals.

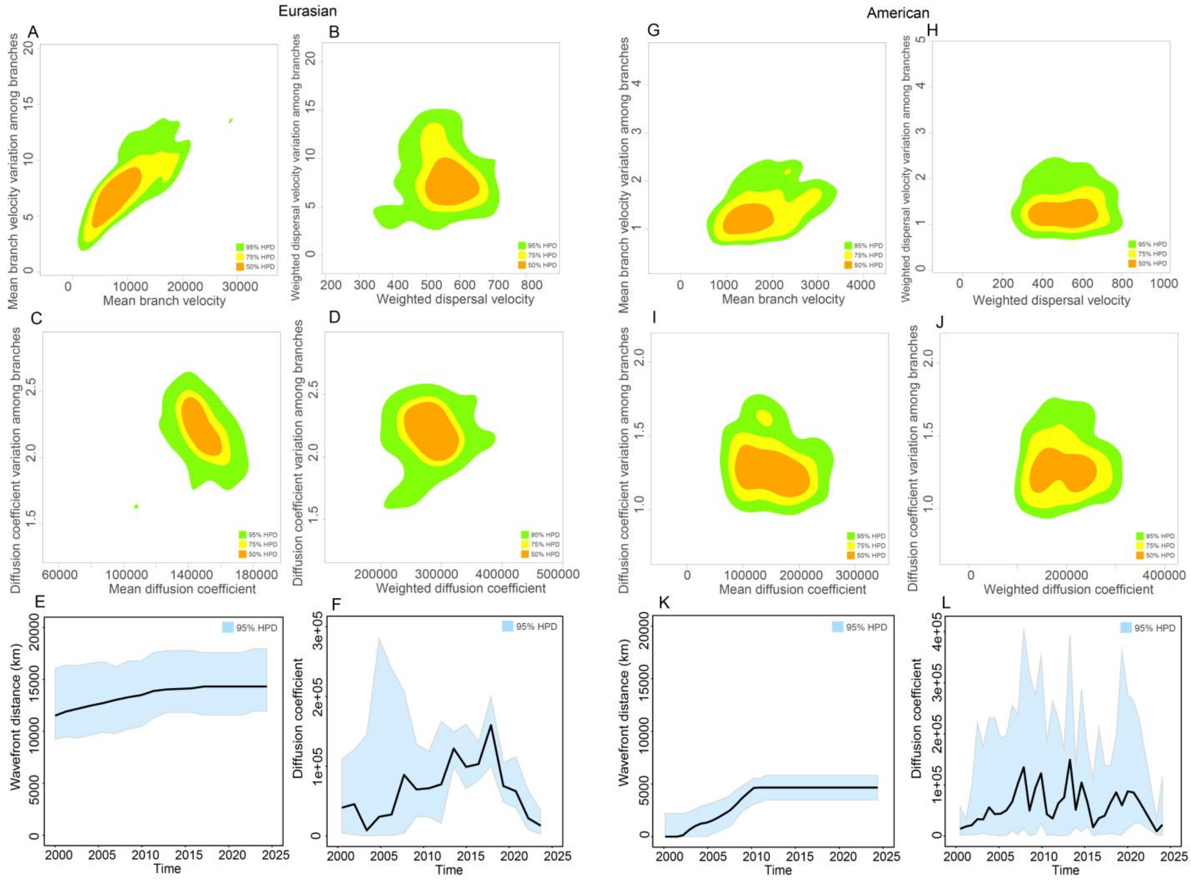

Supplementary Fig. 11. Estimated dispersal statistics for H7N9.

The two columns on the left represent the Eurasian lineage, while the two columns on the right represent the American lineage. A and G, kernel density estimates of mean branch dispersal velocity parameters. B and H, kernel density estimates of weighted branch dispersal velocity parameters. C and I, kernel density estimates of original diffusion coefficient parameters. D and J, kernel density estimates of weighted diffusion coefficient parameters. E and K, furthest extent of epidemic wavefront (spatial distance from epidemic origin). F and L, Evolution of weighted diffusion coefficient. For panels E, F, K, and L, solid lines represent the posterior median values of wavefront distance and diffusion coefficient, and shaded areas denote the 95% HPD intervals.

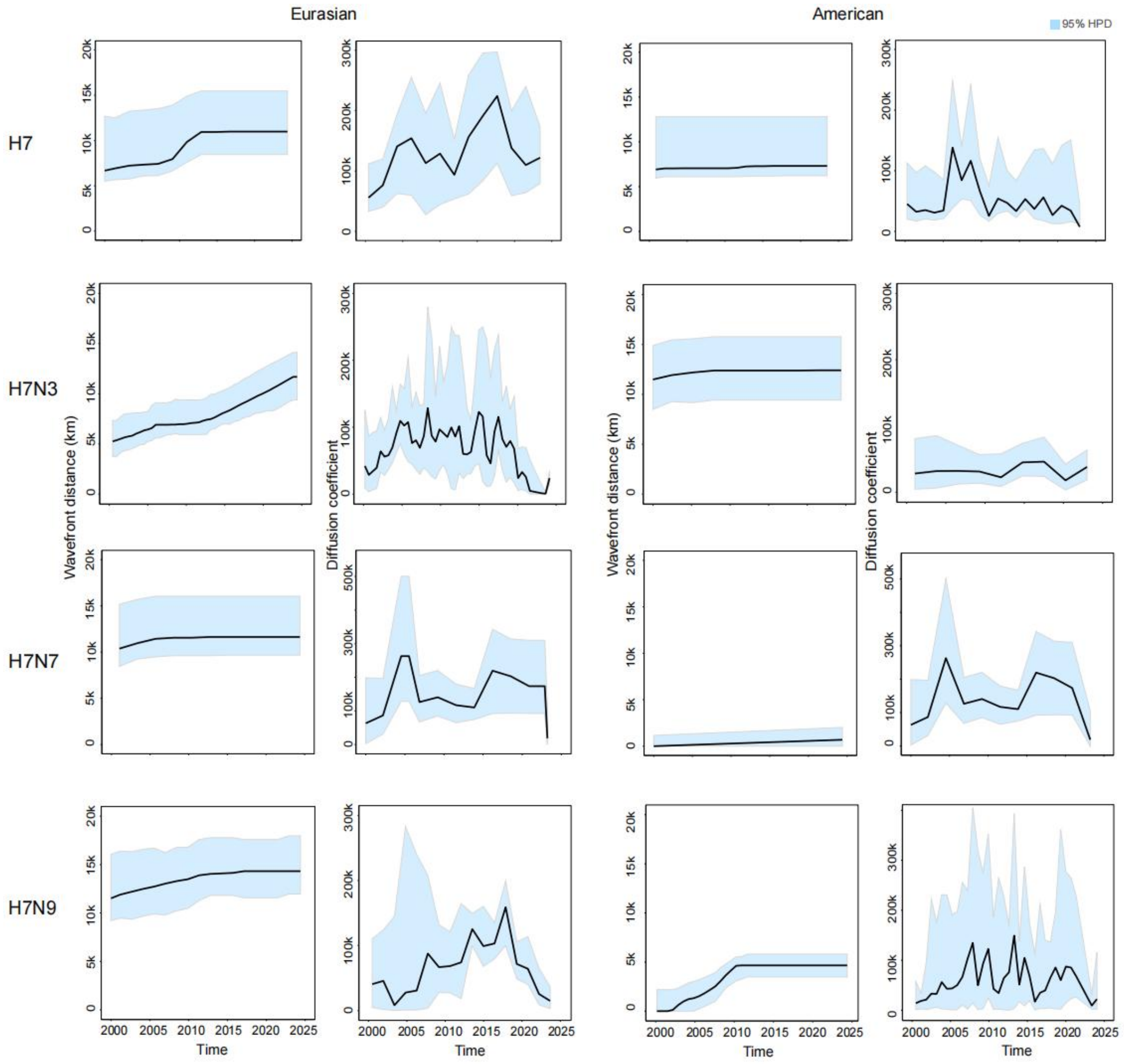

Supplementary Fig. 12. Estimated dispersal statistics of wavefront distance and diffusion coefficient for H7 and subtypes since 2000. Solid lines represent the posterior median values of wavefront distance and diffusion coefficient, and shaded areas denote the 95% HPD intervals.

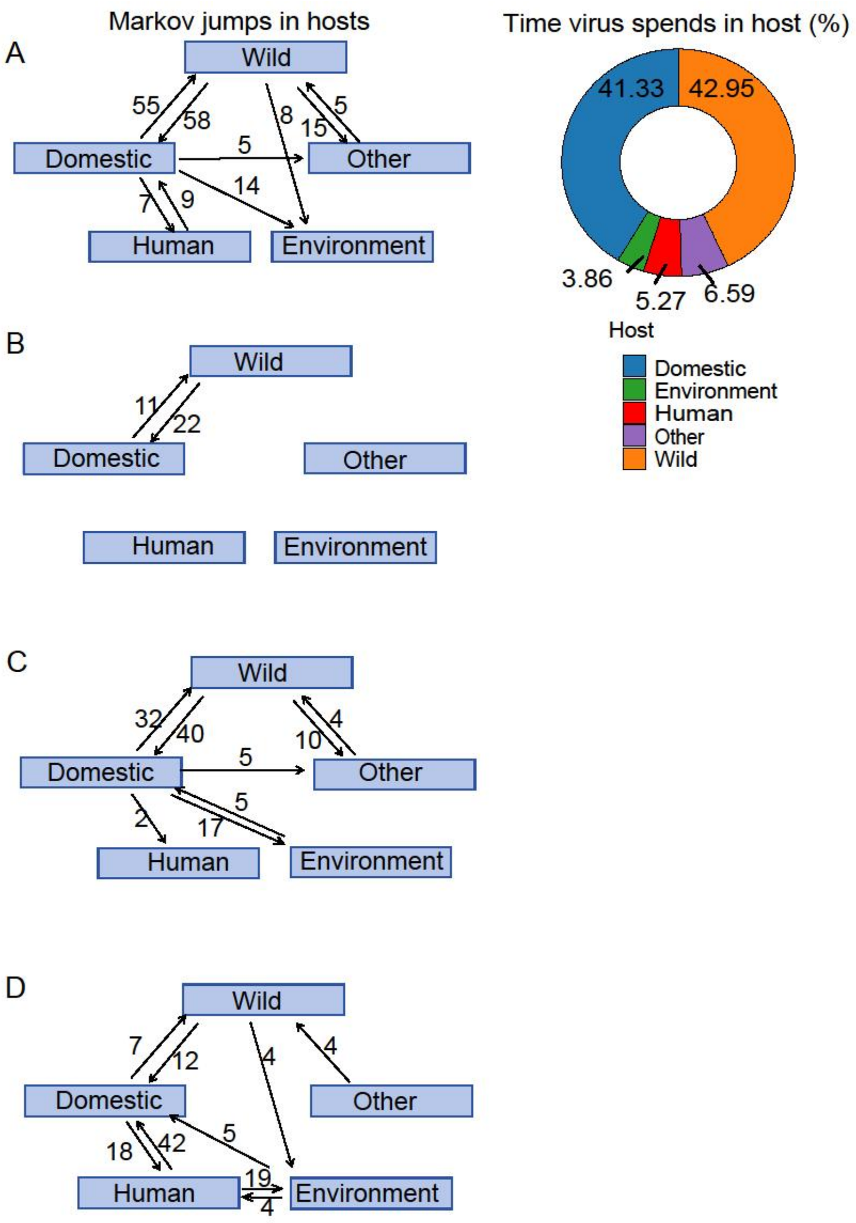

Supplementary Fig. 13. The contrasting host transmission patterns among H7 (A), H7N3 (B), H7N7 (C), and H7N9 (D) inferred from discrete-trait phylogenetics. From left to right, the figures represent host Markov jump counts and Markov rewards.

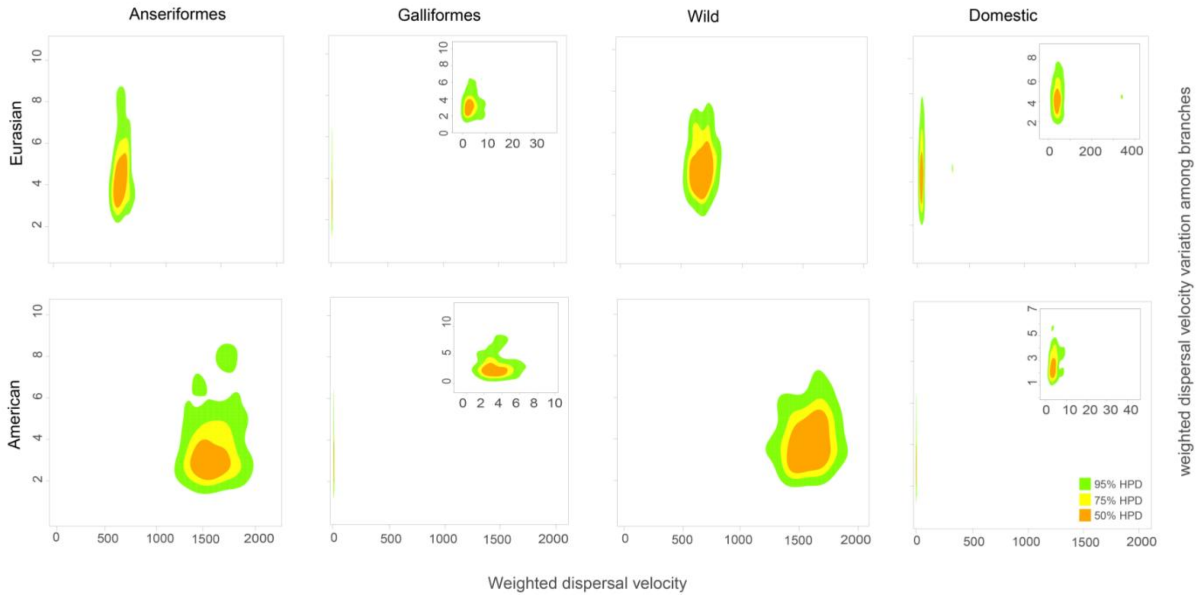

Supplementary Fig. 14. Host-specific dispersal velocity estimates for H7N3.

The top row represents the Eurasian lineage, while the bottom row represents the American lineage. Each panel shows dispersal velocities stratified by host type. Shaded contours represent the 50%, 75%, and 95% highest posterior density (HPD) regions, estimated via kernel density methods, with darker shades indicating higher certainty.

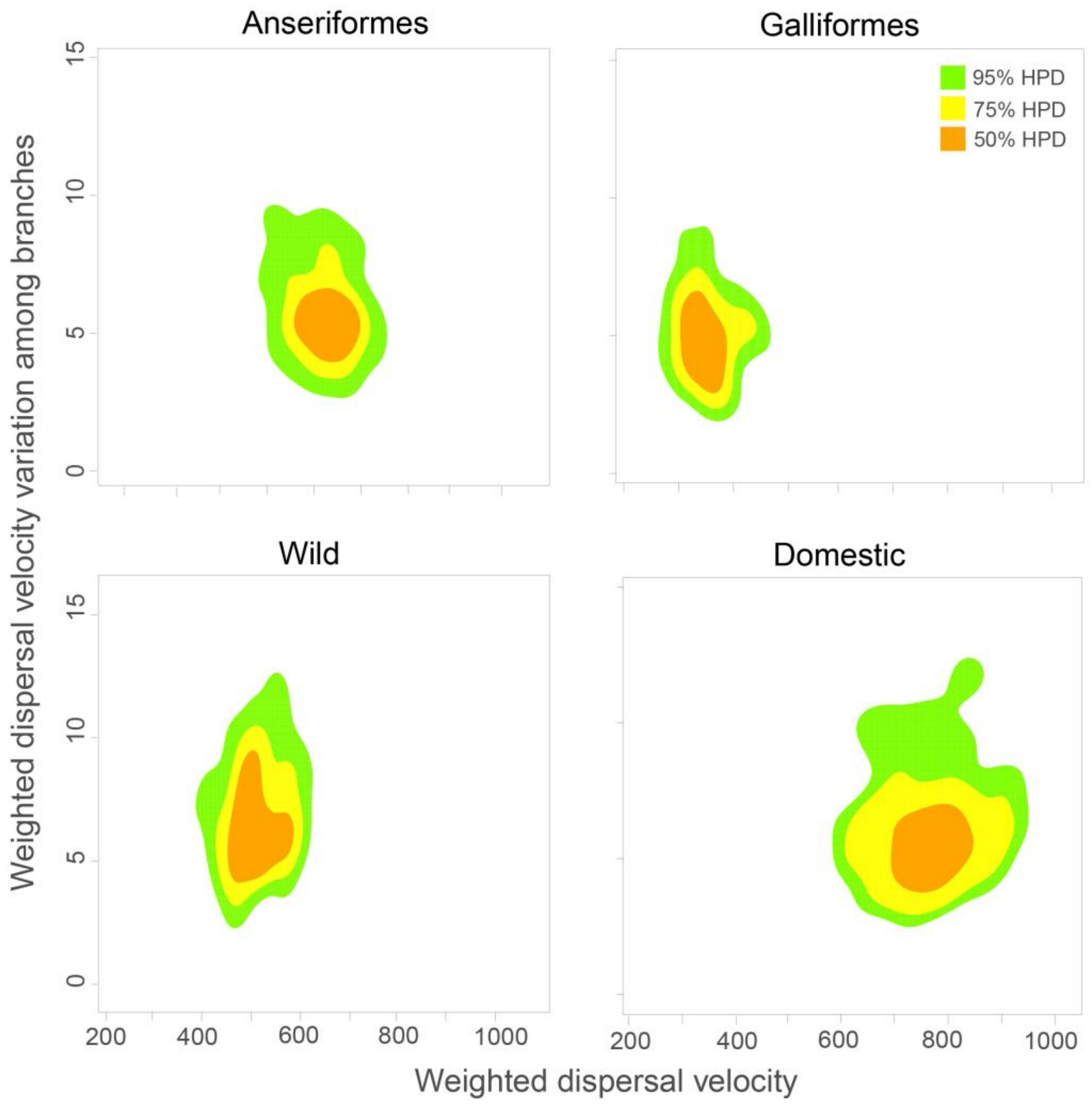

Supplementary Fig. 15. Host-specific dispersal velocity estimates for H7N7.

Each panel shows dispersal velocities stratified by host type. Shaded contours represent the 50%, 75%, and 95% highest posterior density (HPD) regions, estimated via kernel density methods, with darker shades indicating higher certainty.

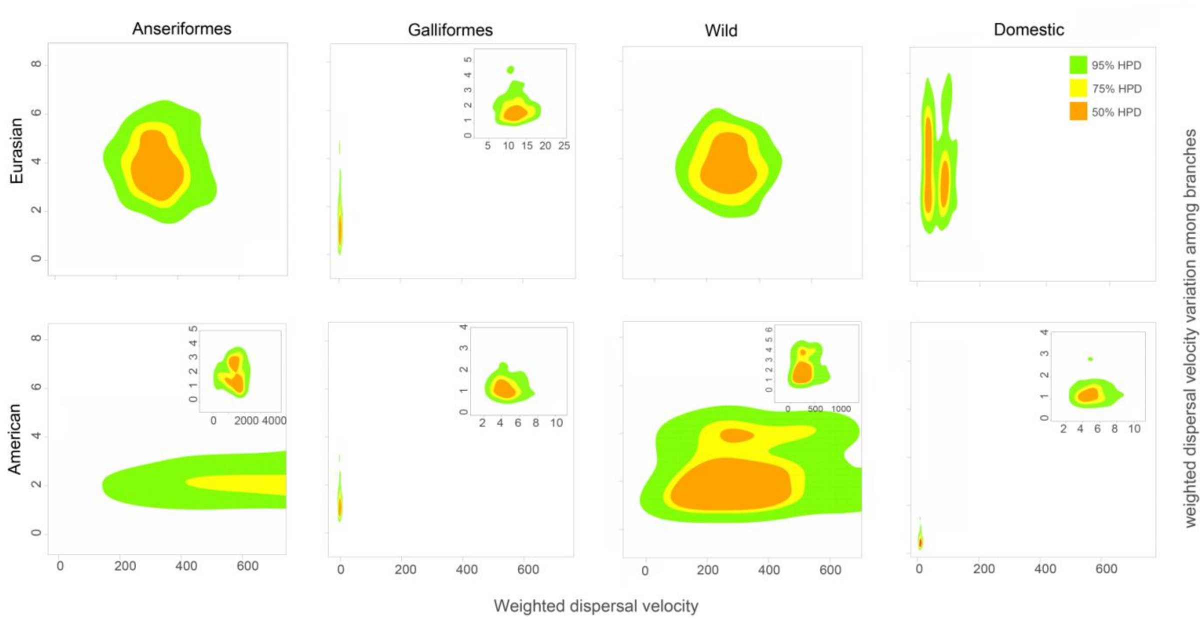

Supplementary Fig. 16. Host-specific dispersal velocity estimates for H7N9.

The top row represents the Eurasian lineage, while the bottom row represents the American lineage. Each panel shows dispersal velocities stratified by host type. Shaded contours represent the 50%, 75%, and 95% highest posterior density (HPD) regions, estimated via kernel density methods, with darker shades indicating higher certainty.

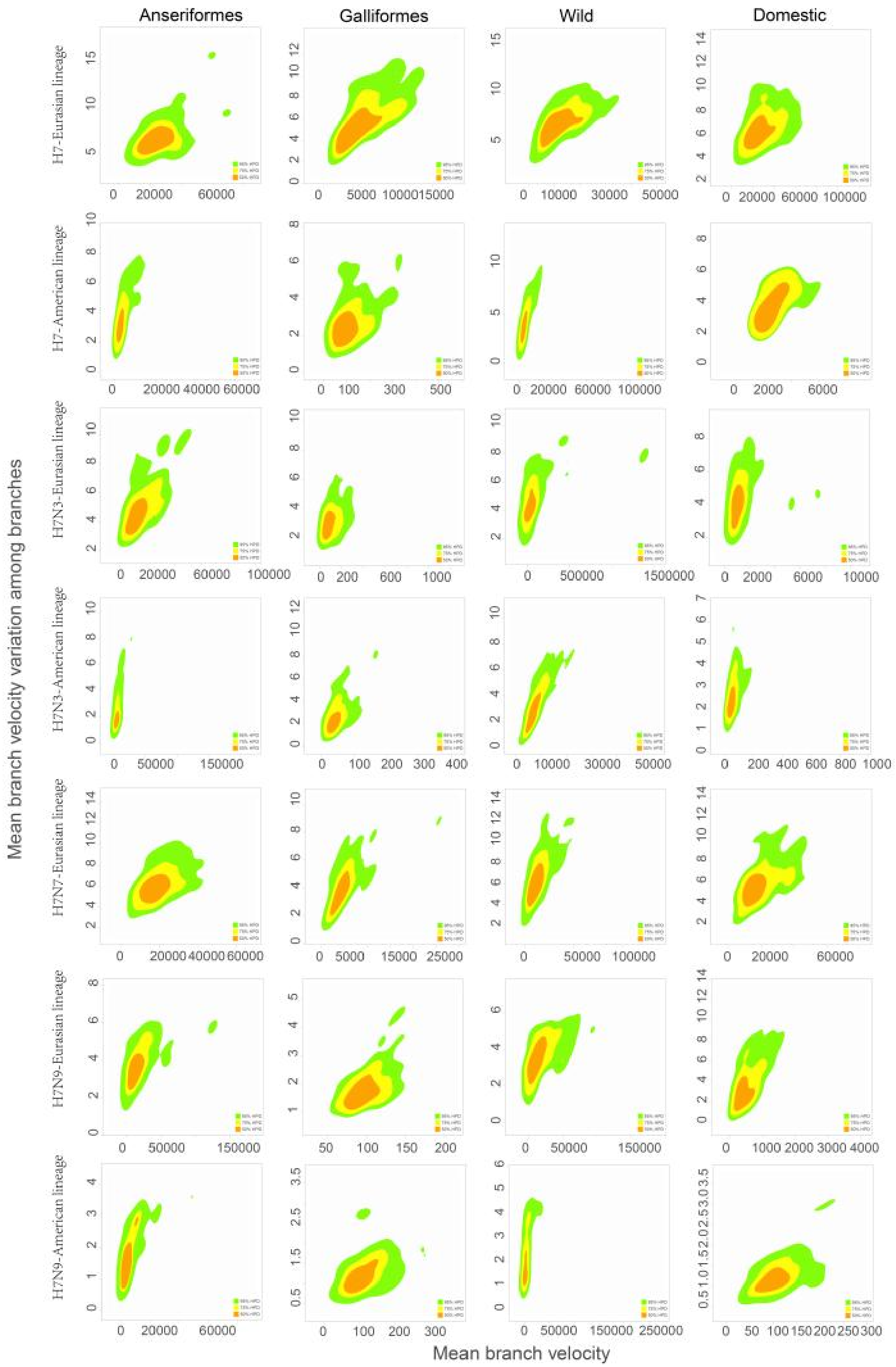

Supplementary Fig. 17. Host-specific mean dispersal velocity estimates for H7 and subtypes. For each graph, the three contours show, in shades of decreasing darkness, the 50%, 75%, and 95% HPD regions via kernel density estimation.

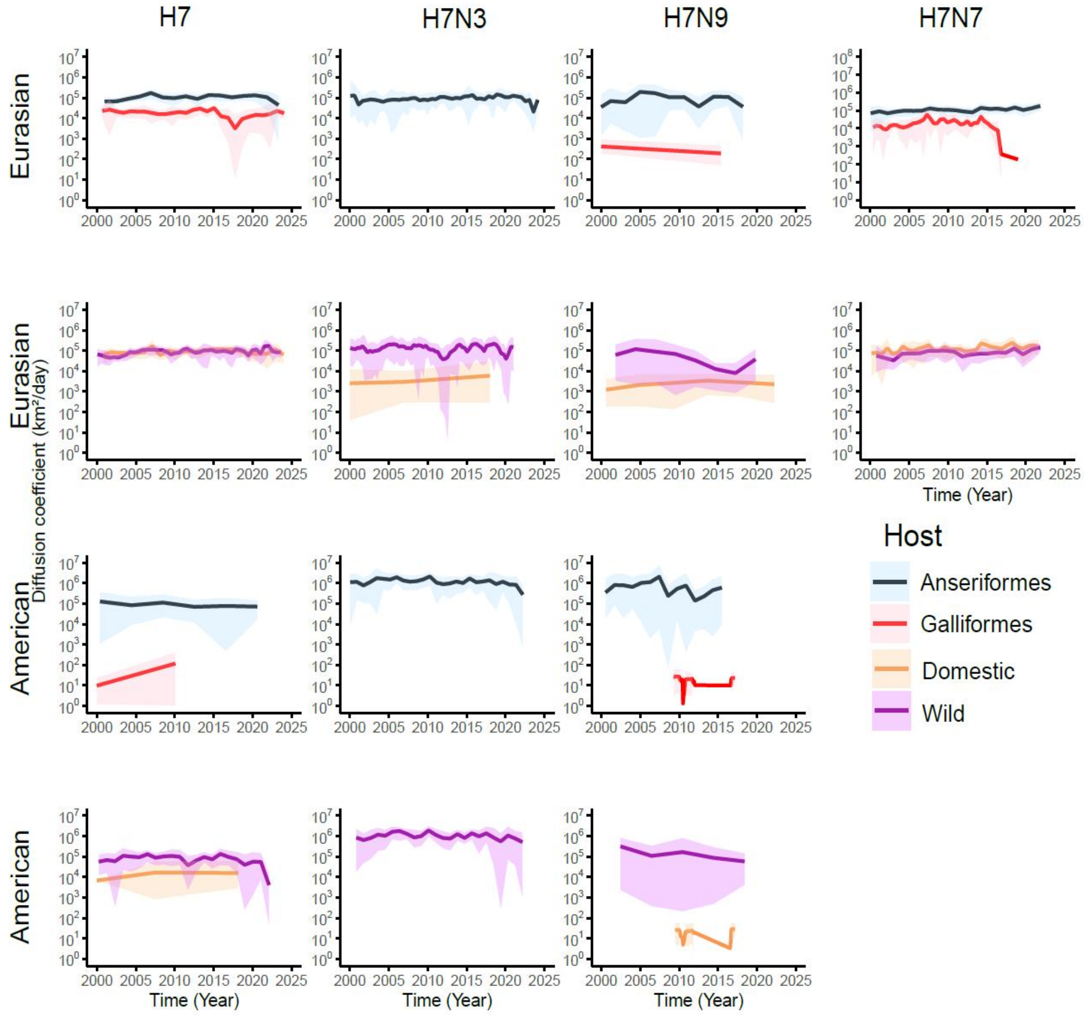

Supplementary Fig. 18. Host-specific and lineage-specific original diffusion coefficient estimates for H7 and subtypes (km^2/day). The shaded areas denote the 95% HPD. Due to insufficient data, the American lineage of H7N7 was not included in the analysis.

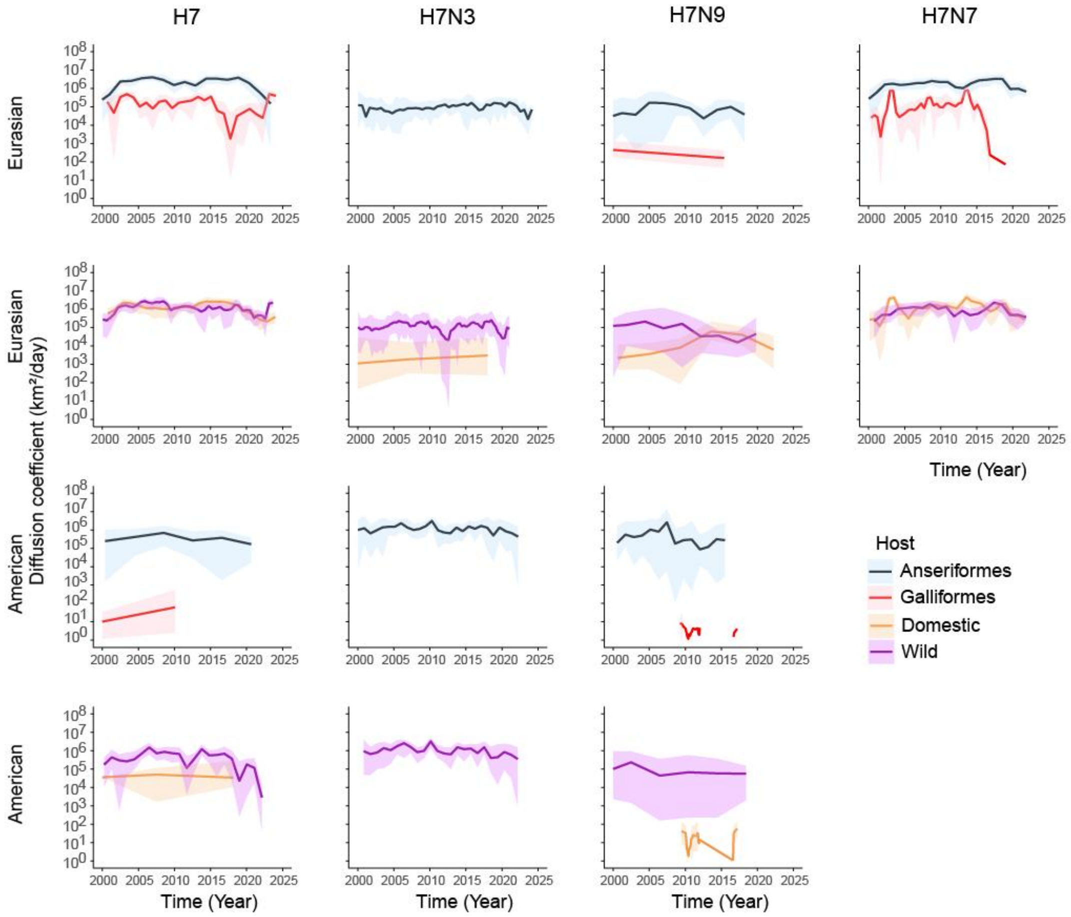

Supplementary Fig. 19. Host-specific and lineage-specific weighted diffusion coefficient estimates for H7 and subtypes (km^2/day). The shaded areas denote the 95% HPD. Due to insufficient data, the American lineage of H7N7 was not included in the analysis. Diffusion estimates for certain years are omitted due to irregular values likely resulting from limited sampling.

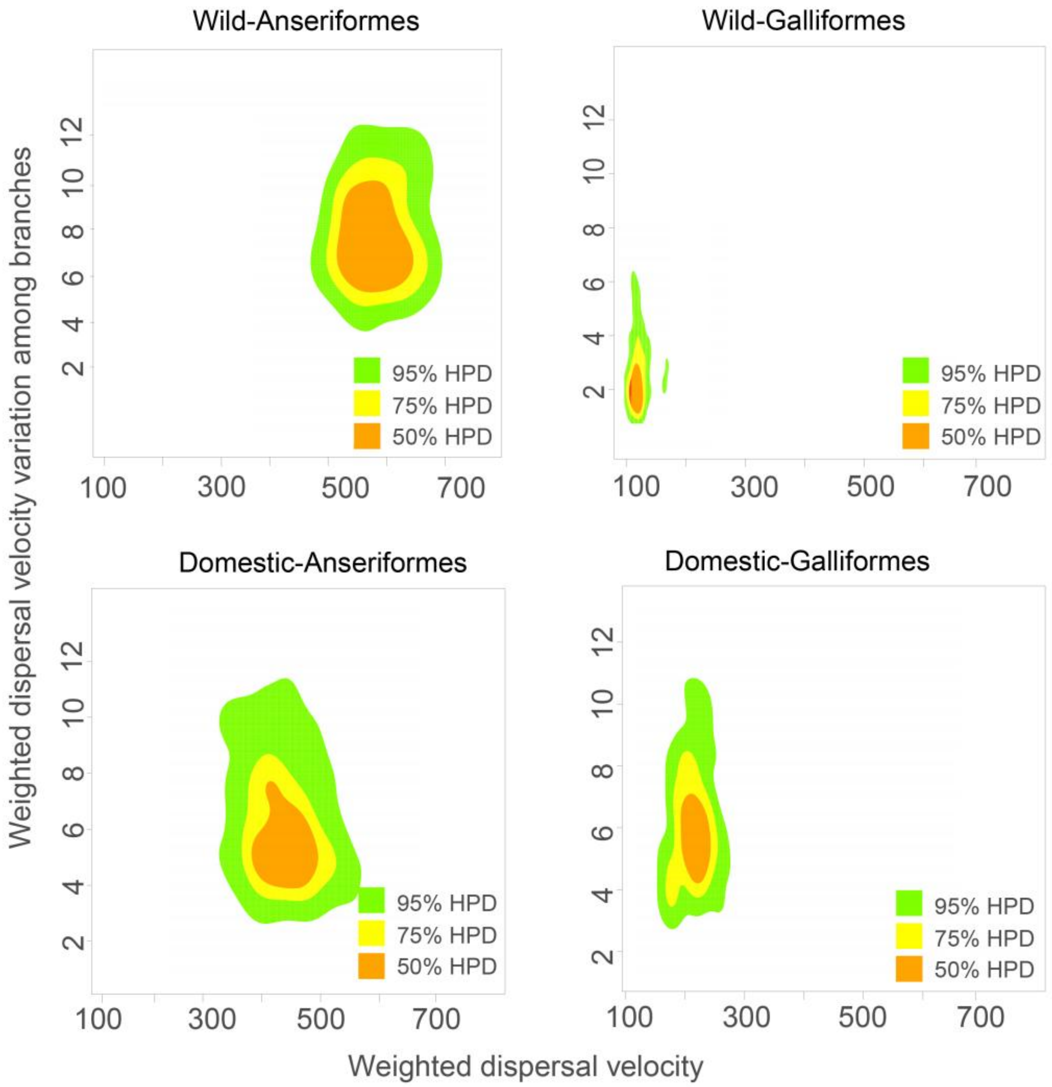

Supplementary Fig. 20. Host-specific weight dispersal velocity estimates for H7 virus. For each graph, the three contours show, in shades of decreasing darkness, the 50%, 75%, and 95% HPD regions via kernel density estimation.

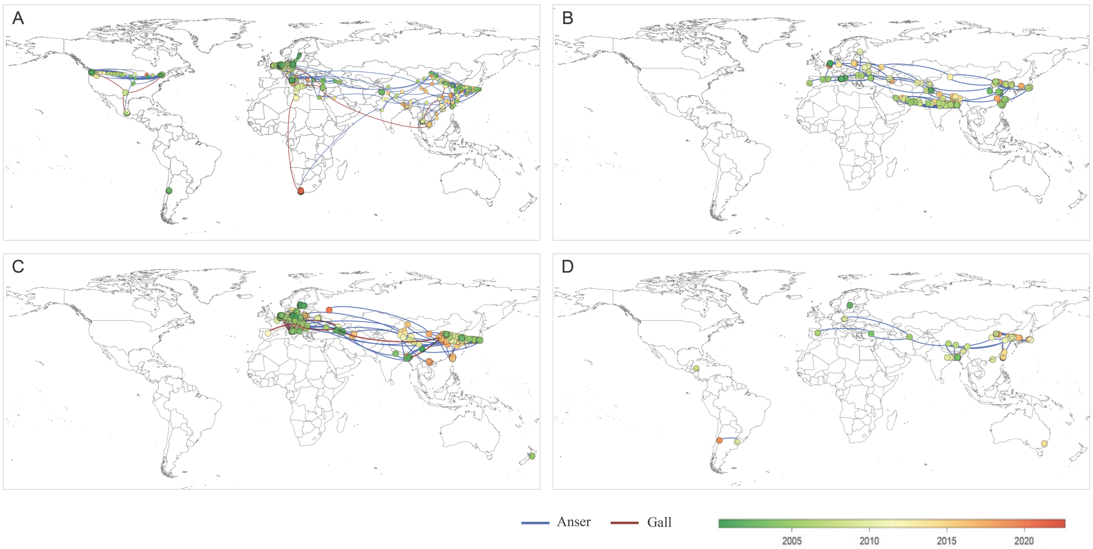

Supplementary Fig. 21. Dynamics of H7 transmission lineages (Anseriformes and Galliformes). H7 (A), H7N3 (B), H7N7 (C), and H7N9 (D). Virus lineage movements were inferred by continuous phylogeographic analysis for each subtype. Maps were generated using the R package rnaturalearth with data from Natural Earth (public domain, https://www.naturalearthdata.com).

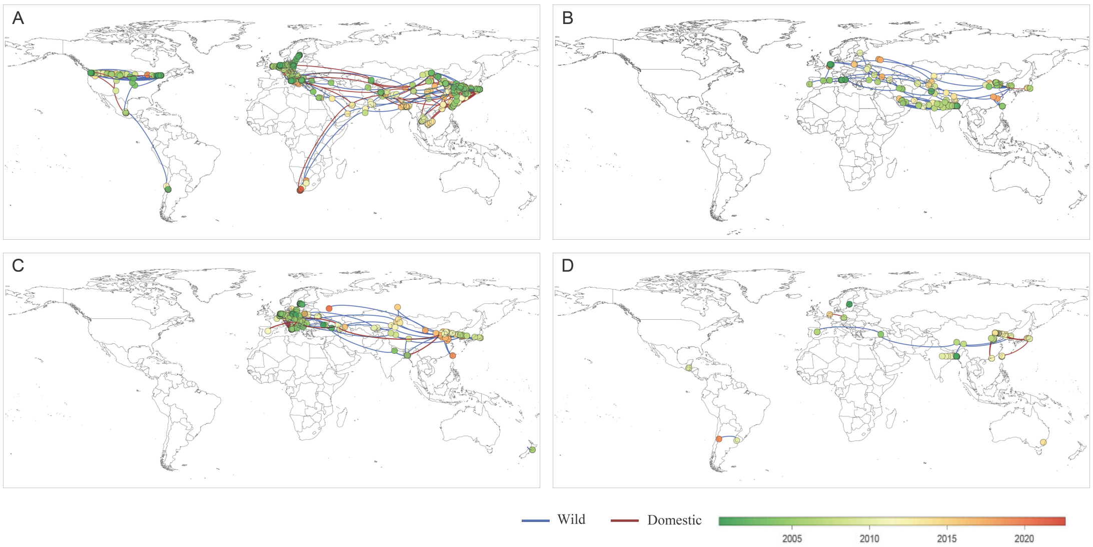

Supplementary Fig. 22. Dynamics of H7 transmission lineages (Wild and domestic birds). H7 (A), H7N3 (B), H7N7 (C), and H7N9 (D). Virus lineage movements were inferred by continuous phylogeographic analysis for each subtype. Maps were generated using the R package rnaturalearth with data from Natural Earth (public domain, https://www.naturalearthdata.com).

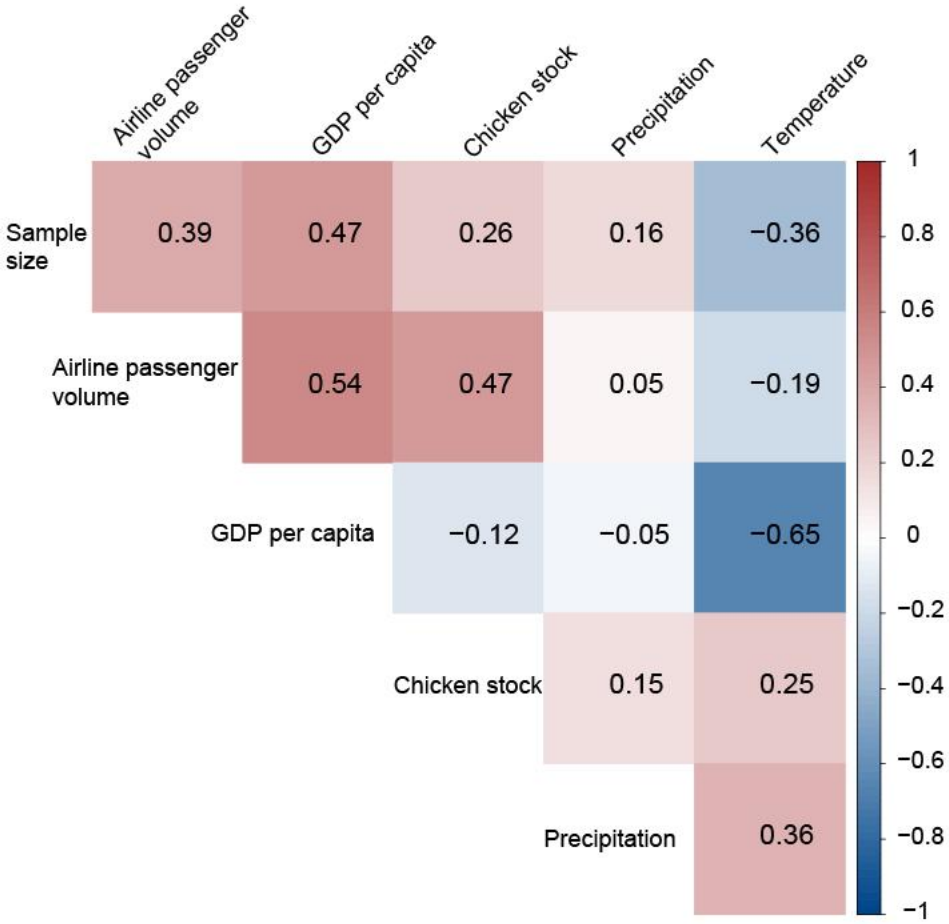

Supplementary Fig. 23. Correlation matrix of predictor variables for generalized linear model analysis. To adhere to a more stringent selection criterion, GDP per capita was excluded from the final model due to its moderate correlation with other variables (e.g., airline passenger volume, r = 0.54; temperature, r = -0.65). After the exclusion of GDP per capita, the absolute values of all remaining correlation coefficients are less than 0.5, indicating low inter-variable correlation and suitability for inclusion in the GLM.

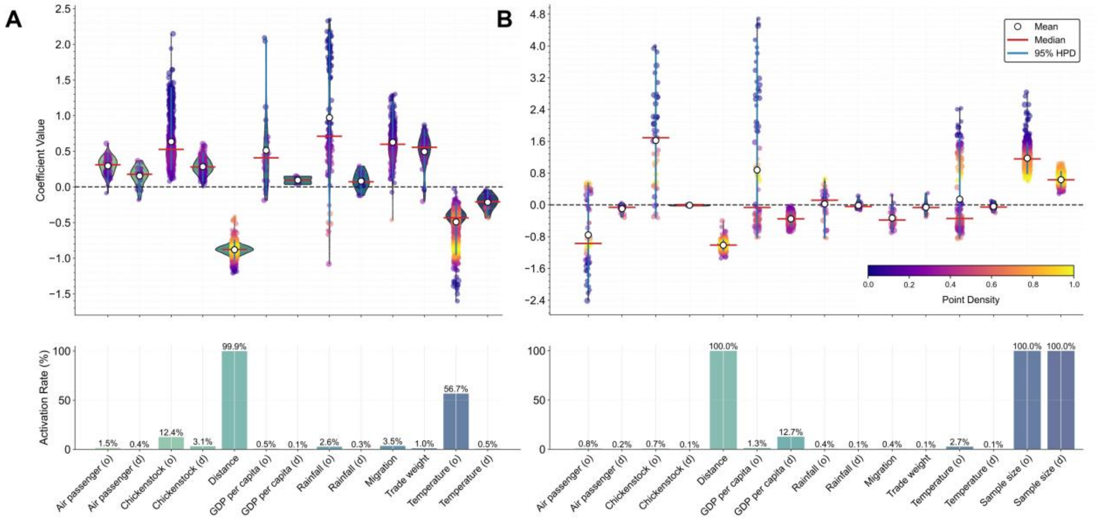

Supplementary Fig. 24. Results for the included predictor GDP per capita in the global H7 virus diffusion analysis. Analyzed predictors include average inter-location distance, monthly airline passenger volume (2010 - 2018), bird migration routes, live poultry trade volume (1996-2016), chicken stock (2000-2023), GDP per capita (2000-2023), annual mean temperature, annual precipitation and sample size. “o” and “d” indicate origin and destination predictors, respectively. Predictor support is expressed as inclusion probability based on indicator expectations.

(A) Model excluding sample size; (B) Model including sample size as a distinctive predictor. Bars show the mean and 95% HPD of GLM coefficient (β) on a log scale, conditioned on the inclusion of each predictor.

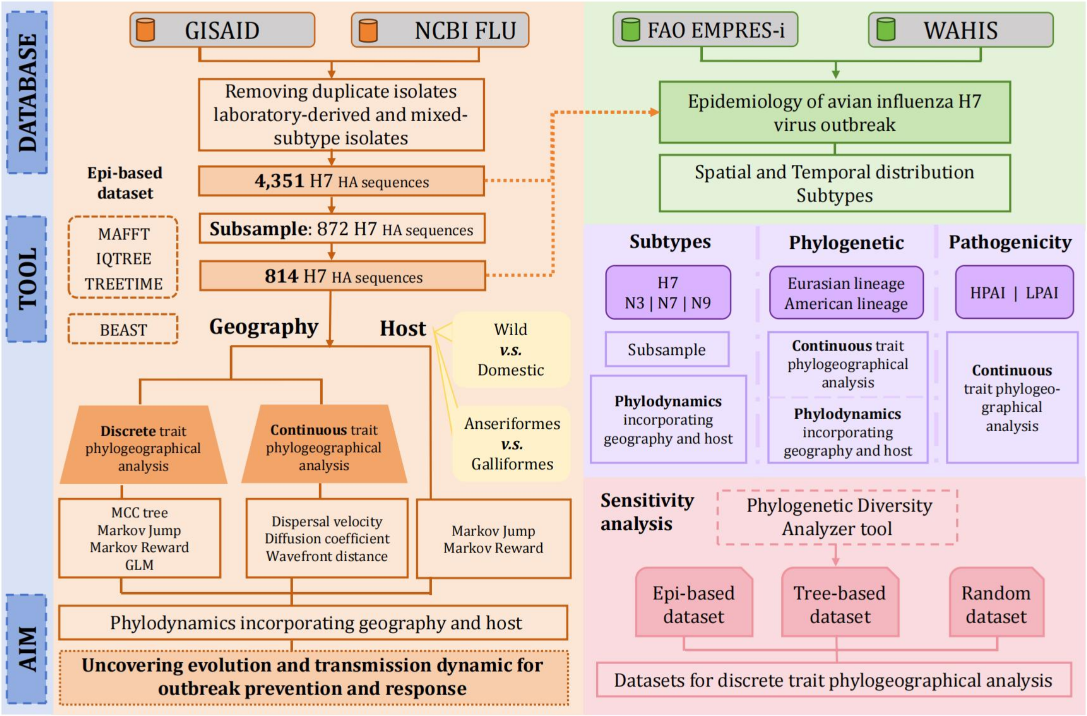

Supplementary Fig. 25. Overview of the analytic pipeline for dataset collection, analytical method and model development evaluation.

Supplementary Table 1. Number of sequences by administrative region.

Continent	Location	Number of sequences before/after down-sampling
Asia	Bangladesh	33/33
Europe	Belgium	21/21
Asia	Cambodia	14/14
North America	Canada	50/50
South America	Chile	33/33
Asia	China	2075/49
Africa	Egypt	21/20
Europe	Germany	29/29
Asia	Hong Kong (SAR) (China)	28/28
Europe	Italy	227/50
Asia	Japan	91/50
Asia	South Korea	132/50
North America	Mexico	63/31
Asia	Mongolia	28/28
Europe	Netherlands	234/50
Asia	Pakistan	29/27
Africa	South Africa	62/50
Europe	Sweden	52/50
Asia	Taiwan (China)	48/47
Asia	Thailand	29/29
Europe	United Kingdom	25/25
North America	USA	921/50

Supplementary Table 2. Bayes factor supports. Comparison of Bayes factor supports for individual transitions between discrete states inferred from the three distinct down-sampled datasets (Epi-based, tree-based and random) of each H7 subtype. Bayes factors >3 and Posterior probability >0.5 are marked in bold.

			Epi-based		Tree-based		Random	
Virus	FROM	TO	Bayes factor	Posterior probability	Bayes factor	Posterior probability	Bayes factor	Posterior probability
H7	Africa	Europe	**10176.897**	**1.000**	**62.064**	**0.974**	1.682	**0.598**
	Asia	Europe	**10176.897**	**1.000**	**14712.719**	**1.000**	**10176.897**	**1.000**
	North America	Europe	**10176.897**	**1.000**	2.143	**0.567**	**10176.897**	**1.000**
	South America	Europe	**10176.897**	**1.000**	0.148	0.083	1.682	**0.598**
	Africa	Asia	**1271.123**	**0.999**	**14712.719**	**1.000**	**37.717**	**0.971**
	South America	North America	**1271.123**	**0.999**	**62.064**	**0.974**	**37.717**	**0.971**
	Asia	Africa	**61.311**	**0.982**	0.110	0.063	**725.871**	**0.998**
	North America	South America	**61.311**	**0.982**	0.074	0.043	**725.871**	**0.998**
	Asia	North America	**10.609**	**0.904**	0.214	0.116	0.361	0.242
	North America	Asia	**10.609**	**0.904**	0.224	0.121	0.361	0.242
	Europe	Asia	0.539	0.323	0.074	0.043	0.425	0.273
	Europe	North America	0.539	0.323	2.143	**0.567**	0.425	0.273
	Europe	Africa	0.241	0.175	**14712.719**	**1.000**	0.614	0.352
	Europe	South America	0.241	0.175	0.224	0.121	0.614	0.352
	Asia	South America	0.182	0.139	**11.828**	**0.879**	0.457	0.288
	North America	Africa	0.182	0.139	0.989	0.377	0.457	0.288
	Africa	South America	0.173	0.132	0.190	0.104	0.354	0.239
	South America	Africa	0.173	0.132	0.199	0.108	0.354	0.239
	Africa	North America	0.153	0.119	0.148	0.083	0.264	0.189
	South America	Asia	0.153	0.119	0.190	0.104	0.264	0.189
	Africa	Oceania	-	-	0.199	0.108	-	-

	Asia	Oceania	-	-	0.200	0.109	-	-
	Europe	Oceania	-	-	0.989	0.377	-	-
	North America	Oceania	-	-	**14712.719**	**1.000**	-	-
	Oceania	Africa	-	-	0.200	0.109	-	-
	Oceania	Asia	-	-	**11.828**	**0.879**	-	-
	Oceania	Europe	-	-	0.214	0.116	-	-
	Oceania	North America	-	-	**14712.719**	**1.000**	-	-
	Oceania	South America	-	-	0.110	0.063	-	-
	South America	Oceania	-	-	**14712.719**	**1.000**	-	-
H7N3	Asia	Europe	**14712.719**	**1.000**	**14712.719**	**1.000**	**14712.719**	**1.000**
	Oceania	North America	**14712.719**	**1.000**	**14712.719**	**1.000**	**14712.719**	**1.000**
	Europe	Africa	**979.322**	**0.998**	**3676.954**	**1.000**	**3676.954**	**1.000**
	North America	South America	**979.322**	**0.998**	**3676.954**	**1.000**	**3676.954**	**1.000**
	Africa	Asia	**9.658**	**0.855**	**20.004**	**0.924**	**19.877**	**0.924**
	South America	Oceania	**9.658**	**0.855**	**20.004**	**0.924**	**19.877**	**0.924**
	Asia	Oceania	**4.131**	**0.716**	1.887	**0.536**	1.485	0.476
	Oceania	Asia	**4.131**	**0.716**	1.887	**0.536**	1.485	0.476
	Africa	Europe	**4.120**	**0.716**	1.467	0.473	1.341	0.451
	South America	North America	**4.120**	**0.716**	1.467	0.473	1.341	0.451
	Asia	South America	1.041	0.389	0.454	0.217	0.393	0.194
	Oceania	Africa	1.041	0.389	0.454	0.217	0.393	0.194
	Europe	Oceania	0.524	0.243	0.677	0.293	0.876	0.349
	North America	Asia	0.524	0.243	0.677	0.293	0.876	0.349
	Asia	North America	0.505	0.236	0.577	0.261	0.532	0.245
	Oceania	Europe	0.505	0.236	0.577	0.261	0.532	0.245
	Europe	South America	0.498	0.233	0.350	0.177	0.358	0.180

	North America	Africa	0.498	0.233	0.350	0.177	0.358	0.180
	Africa	Oceania	0.432	0.209	0.501	0.235	0.491	0.231
	South America	Asia	0.432	0.209	0.501	0.235	0.491	0.231
	Africa	South America	0.373	0.186	0.311	0.160	0.315	0.162
	South America	Africa	0.373	0.186	0.311	0.160	0.315	0.162
	Asia	Africa	0.348	0.176	0.600	0.268	0.630	0.278
	Oceania	South America	0.348	0.176	0.600	0.268	0.630	0.278
	Europe	North America	0.279	0.146	0.366	0.183	0.393	0.194
	North America	Europe	0.279	0.146	0.366	0.183	0.393	0.194
	Africa	North America	0.215	0.116	0.271	0.142	0.253	0.134
	South America	Europe	0.215	0.116	0.271	0.142	0.253	0.134
	Europe	Asia	0.199	0.108	0.573	0.260	0.655	0.286
	North America	Oceania	0.199	0.108	0.573	0.260	0.655	0.286
H7N7	Africa	Europe	**10176.897**	**1.000**	**1130.239**	**0.999**	**523.878**	**0.997**
	Asia	Europe	**10176.897**	**1.000**	**14712.719**	**1.000**	**14712.719**	**1.000**
	North America	Europe	**10176.897**	**1.000**	**3.483**	**0.681**	**12.834**	**0.887**
	Oceania	Europe	**10176.897**	**1.000**	0.254	0.135	0.189	0.104
	Africa	Oceania	**3.755**	**0.769**	1.095	0.401	0.769	0.320
	Oceania	Africa	**3.755**	**0.769**	0.346	0.175	0.351	0.177
	Africa	Asia	2.419	**0.681**	**4.996**	**0.753**	**5.722**	**0.778**
	Oceania	North America	2.419	**0.681**	**14712.719**	**1.000**	**14712.719**	**1.000**
	Europe	Asia	0.887	0.440	0.370	0.184	0.206	0.112
	Europe	North America	0.887	0.440	**3.483**	**0.681**	**12.834**	**0.887**
	Africa	North America	0.835	0.425	0.427	0.207	0.257	0.136
	Oceania	Asia	0.835	0.425	0.260	0.137	**3.377**	**0.674**
	Europe	Africa	0.502	0.307	0.648	0.284	0.770	0.320

	Europe	Oceania	0.502	0.307	2.434	**0.598**	0.859	0.344
	Asia	North America	0.444	0.282	0.254	0.135	0.189	0.104
	North America	Asia	0.444	0.282	2.434	**0.598**	0.859	0.344
	Asia	Africa	0.300	0.210	0.603	0.270	0.288	0.150
	North America	Oceania	0.300	0.210	0.370	0.184	0.206	0.112
	Asia	Oceania	0.167	0.129	0.260	0.137	**3.377**	**0.674**
	North America	Africa	0.167	0.129	1.078	0.397	1.510	0.480
	Africa	South America	-	-	0.613	0.273	0.535	0.247
	Asia	South America	-	-	0.346	0.175	0.351	0.177
	Europe	South America	-	-	1.078	0.397	1.510	0.480
	North America	South America	-	-	0.648	0.284	0.770	0.320
	Oceania	South America	-	-	0.603	0.270	0.288	0.150
	South America	Africa	-	-	0.613	0.273	0.535	0.247
	South America	Asia	-	-	1.095	0.401	0.769	0.320
	South America	Europe	-	-	0.427	0.207	0.257	0.136
	South America	North America	-	-	**1130.239**	**0.999**	**523.878**	**0.997**
	South America	Oceania	-	-	**4.996**	**0.753**	**5.722**	**0.778**
H7N9	Asia	Europe	**918.012**	**0.998**	**124.129**	**0.987**	**23.095**	**0.934**
	Oceania	North America	**918.012**	**0.998**	**124.129**	**0.987**	**23.095**	**0.934**
	Asia	North America	**18.945**	**0.921**	**22.213**	**0.931**	0.506	0.236
	Oceania	Europe	**18.945**	**0.921**	**22.213**	**0.931**	0.506	0.236
	Africa	Europe	**5.592**	**0.774**	**6.778**	**0.806**	**8.065**	**0.831**
	South America	North America	**5.592**	**0.774**	**6.778**	**0.806**	**8.065**	**0.831**
	Africa	Asia	**3.074**	**0.653**	2.769	**0.629**	0.723	0.307
	South America	Oceania	**3.074**	**0.653**	2.769	**0.629**	0.723	0.307
	Europe	Africa	**3.041**	**0.650**	**3.681**	**0.692**	**4.064**	**0.713**

North America	South America	**3.041**	**0.650**	**3.681**	**0.692**	**4.064**	**0.713**
Europe	Oceania	1.235	0.430	0.601	0.269	0.577	0.261
North America	Asia	1.235	0.430	0.601	0.269	0.577	0.261
Asia	Oceania	0.930	0.363	1.165	0.416	**6.769**	**0.805**
Oceania	Asia	0.930	0.363	1.165	0.416	**6.769**	**0.805**
Europe	Asia	0.687	0.296	1.325	0.448	0.442	0.213
North America	Oceania	0.687	0.296	1.325	0.448	0.442	0.213
Europe	South America	0.547	0.251	0.418	0.204	0.995	0.378
North America	Africa	0.547	0.251	0.418	0.204	0.995	0.378
Europe	North America	0.542	0.249	0.377	0.188	1.372	0.456
North America	Europe	0.542	0.249	0.377	0.188	1.372	0.456
Asia	South America	0.498	0.234	0.459	0.219	0.437	0.211
Oceania	Africa	0.498	0.234	0.459	0.219	0.437	0.211
Asia	Africa	0.485	0.229	0.740	0.312	0.450	0.216
Oceania	South America	0.485	0.229	0.740	0.312	0.450	0.216
Africa	Oceania	0.464	0.221	0.423	0.206	0.454	0.217
South America	Asia	0.464	0.221	0.423	0.206	0.454	0.217
Africa	South America	0.352	0.177	0.362	0.181	0.658	0.287
South America	Africa	0.352	0.177	0.362	0.181	0.658	0.287
Africa	North America	0.306	0.158	0.342	0.173	0.690	0.297
South America	Europe	0.306	0.158	0.342	0.173	0.690	0.297

Supplementary Table 3. The Markov jumps for H7 and subtypes by region for epi-based dataset.

Subtype	Region Transition			
	Region (from)	Region (to)	Markov Jumps	Percentage (%)
H7	Africa	Asia	2.3950	0.0720
	Africa	Europe	2.1780	0.0654
	Africa	North America	0.0461	0.0014
	Africa	South America	0.0432	0.0013
	Asia	Africa	3.5640	0.1071
	Asia	Europe	7.9860	0.2399
	Asia	North America	1.7280	0.0519
	Asia	South America	0.0784	0.0024
	Europe	Africa	4.4260	0.1330
	Europe	Asia	6.9080	0.2075
	Europe	North America	0.1010	0.0030
	Europe	South America	0.2800	0.0084
	North America	Africa	0.0401	0.0012
	North America	Asia	0.8980	0.0270
	North America	Europe	0.2970	0.0089
	North America	South America	1.0050	0.0302
	South America	Africa	0.0167	0.0005
	South America	Asia	0.0329	0.0010
	South America	Europe	0.0644	0.0019
	South America	North America	1.1990	0.0360
H7N3	Africa	Asia	0.32	1.6913
	Africa	Europe	0.16	0.8457
	Africa	South America	0.08	0.4228
	Africa	North America	0.04	0.2114
	Africa	Oceania	0.13	0.6871
	Asia	South America	0.08	0.4228
	Asia	Africa	1.53	8.0867
	Asia	Europe	5.37	28.3827
	Asia	North America	0.18	0.9514
	Asia	Oceania	0.79	4.1755
	Europe	South America	0.19	1.0042
	Europe	Africa	0.96	5.0740
	Europe	Asia	4.84	25.5814
	Europe	North America	0.09	0.4757
	Europe	Oceania	0.21	1.1099
	North America	South America	0.11	0.5814
	North America	Africa	0.05	0.2643
	North America	Asia	0.18	0.9514
	North America	Europe	0.07	0.3700

	North America	Oceania	0.04	0.2114
	Oceania	South America	1.73	9.1438
	Oceania	Africa	0.04	0.2114
	Oceania	Asia	0.15	0.7928
	Oceania	Europe	0.05	0.2643
	Oceania	North America	0.04	0.2114
	South America	Africa	0.08	0.4228
	South America	Asia	0.26	1.3742
	South America	Europe	0.13	0.6871
	South America	North America	0.92	4.8626
	South America	Oceania	0.10	0.5285
H7N7	Africa	Asia	0.64	2.7495
	Africa	Europe	1.27	5.5034
	Africa	North America	0.38	1.6342
	Africa	Oceania	0.82	3.5493
	Asia	Africa	1.20	5.1705
	Asia	Europe	5.76	24.8798
	Asia	North America	0.23	0.9943
	Asia	Oceania	0.07	0.2955
	Europe	Africa	5.67	24.5080
	Europe	Asia	5.56	24.0368
	Europe	North America	0.40	1.7422
	Europe	Oceania	0.31	1.3359
	North America	Africa	0.14	0.6052
	North America	Asia	0.10	0.4410
	North America	Europe	0.15	0.6528
	North America	Oceania	0.06	0.2745
	Oceania	Africa	0.16	0.6787
	Oceania	Asia	0.03	0.1456
	Oceania	Europe	0.07	0.3186
	Oceania	North America	0.11	0.4842
H7N9	Africa	Asia	0.245	1.4944
	Africa	Europe	0.329	2.0067
	Africa	North America	0.0681	0.4154
	Africa	Oceania	0.151	0.9210
	Africa	South America	0.09799	0.5977
	Asia	Africa	1.15	7.0144
	Asia	Europe	3.181	19.4023
	Asia	North America	2.056	12.5405
	Asia	Oceania	0.391	2.3849
	Asia	South America	0.219	1.3358
	Europe	Africa	1.479	9.0211
	Europe	Asia	2.706	16.5051

Europe	North America	0.276	1.6834
Europe	Oceania	0.462	2.8179
Europe	South America	0.222	1.3541
North America	Africa	0.09343	0.5699
North America	Asia	0.836	5.0991
North America	Europe	0.176	1.0735
North America	Oceania	0.289	1.7627
North America	South America	0.756	4.6112
Oceania	Africa	0.07166	0.4371
Oceania	Asia	0.11	0.6709
Oceania	Europe	0.152	0.9271
Oceania	North America	0.144	0.8783
Oceania	South America	0.134	0.8173
South America	Africa	0.05055	0.3083
South America	Asia	0.06821	0.4160
South America	Europe	0.09399	0.5733
South America	North America	0.261	1.5920
South America	Oceania	0.126	0.7685

Supplementary Table 4. The Markov rewards for H7 and subtypes by region.

Subtype	Region	Reward Time (year)	Percentage (%)
H7	Africa	62.908	4.80
	Asia	558.194	42.63
	Europe	349.981	26.73
	North America	275.364	21.03
	South America	62.908	4.80

Supplementary Table 5. The Markov jumps for H7 and subtypes by host (Anseriformes and Galliformes).

Subtype	Host Transition			
	Host (from)	Host (to)	Markov Jumps	Percentage (%)
H7-Eurasian lineage	Anseriformes	Environment	23.930	23.50
	Anseriformes	Galliformes	29.370	28.85
	Anseriformes	Human	1.129	1.11
	Anseriformes	Other	13.096	12.86
	Environment	Anseriformes	3.352	3.29
	Environment	Galliformes	0.015	0.01
	Environment	Human	0.011	0.01
	Environment	Other	0.018	0.02
	Galliformes	Anseriformes	14.812	14.55
	Galliformes	Environment	0.014	0.01
	Galliformes	Human	2.958	2.91
	Galliformes	Other	0.347	0.34
	Human	Anseriformes	2.832	2.78
	Human	Environment	0.011	0.01
	Human	Galliformes	7.099	6.97
	Human	Other	0.016	0.02
	Other	Anseriformes	2.618	2.57
	Other	Environment	0.014	0.01
	Other	Galliformes	0.160	0.16
	Other	Human	0.012	0.01
H7-American lineage	Anseriformes	Galliformes	8.011	35.21
	Anseriformes	Human	0.033	0.15
	Anseriformes	Other	4.143	18.21
	Galliformes	Anseriformes	3.640	16.00
	Galliformes	Human	2.991	13.15
	Galliformes	Other	2.282	10.03
	Human	Anseriformes	0.015	0.07
	Human	Galliformes	0.491	2.16
	Human	Other	0.028	0.12
	Other	Anseriformes	0.608	2.67
	Other	Galliformes	0.464	2.04
	Other	Human	0.044	0.19
H7N3-Eurasian lineage	Anseriformes	Galliformes	9.903	48.42
	Anseriformes	Other	5.316	25.99
	Galliformes	Anseriformes	4.532	22.16
	Galliformes	Other	0.031	0.15
	Other	Anseriformes	0.642	3.14
	Other	Galliformes	0.028	0.14
H7N3-American lineage	Anseriformes	Environment	0.850	3.36
	Anseriformes	Galliformes	6.583	26.03

	Anseriformes	Other	12.110	47.89
	Environment	Anseriformes	0.054	0.21
	Environment	Galliformes	0.019	0.07
	Environment	Other	0.022	0.09
	Galliformes	Anseriformes	2.134	8.44
	Galliformes	Environment	0.073	0.29
	Galliformes	Other	0.729	2.88
	Other	Anseriformes	2.369	9.37
	Other	Environment	0.161	0.64
	Other	Galliformes	0.184	0.73
H7N7-Eurasian lineage	Anseriformes	Environment	17.334	22.06
	Anseriformes	Galliformes	18.437	23.46
	Anseriformes	Human	0.006	0.01
	Anseriformes	Other	23.540	29.95
	Environment	Anseriformes	3.732	4.75
	Environment	Galliformes	0.011	0.01
	Environment	Human	0.004	0.00
	Environment	Other	0.429	0.55
	Galliformes	Anseriformes	4.995	6.36
	Galliformes	Environment	0.011	0.01
	Galliformes	Human	2.091	2.66
	Galliformes	Other	0.838	1.07
	Human	Anseriformes	0.006	0.01
	Human	Environment	0.005	0.01
	Human	Galliformes	0.027	0.03
	Human	Other	0.006	0.01
	Other	Anseriformes	6.143	7.82
	Other	Environment	0.371	0.47
	Other	Galliformes	0.603	0.77
	Other	Human	0.005	0.01
H7N9-Eurasian lineage	Anseriformes	Environment	4.162	4.05
	Anseriformes	Galliformes	1.849	1.80
	Anseriformes	Human	2.965	2.88
	Anseriformes	Other	3.493	3.40
	Environment	Anseriformes	1.185	1.15
	Environment	Galliformes	0.754	0.73
	Environment	Human	4.007	3.90
	Environment	Other	0.229	0.22
	Galliformes	Anseriformes	2.114	2.06
	Galliformes	Environment	2.091	2.03
	Galliformes	Human	16.205	15.76
	Galliformes	Other	0.171	0.17
	Human	Anseriformes	5.308	5.16
	Human	Environment	19.766	19.22

	Human	Galliformes	32.893	31.99
	Human	Other	3.849	3.74
	Other	Anseriformes	1.412	1.37
	Other	Environment	0.071	0.07
	Other	Galliformes	0.022	0.02
	Other	Human	0.284	0.28
H7N9-American lineage	Anseriformes	Environment	1.061	8.53
	Anseriformes	Galliformes	4.182	33.64
	Anseriformes	Human	0.512	4.12
	Anseriformes	Other	1.044	8.40
	Environment	Anseriformes	0.166	1.34
	Environment	Galliformes	0.025	0.20
	Environment	Human	0.120	0.97
	Environment	Other	0.082	0.66
	Galliformes	Anseriformes	2.828	22.75
	Galliformes	Environment	0.144	1.16
	Galliformes	Human	0.406	3.27
	Galliformes	Other	0.240	1.93
	Human	Anseriformes	0.088	0.71
	Human	Environment	0.044	0.35
	Human	Galliformes	0.043	0.34
	Human	Other	0.082	0.66
	Other	Anseriformes	0.890	7.16
	Other	Environment	0.113	0.91
	Other	Galliformes	0.186	1.50
	Other	Human	0.176	1.42

Supplementary Table 6. The Markov rewards for H7 and subtypes by host (Anseriformes and Galliformes).

Subtype	Host	Reward Time (year)	Percentage (%)
H7-Eurasian lineage	Anseriformes	543.875	56.03
	Galliformes	52.105	5.37
	Environment	228.241	23.51
	Human	67.025	6.90
	Other	79.433	8.18
H7-American lineage	Anseriformes	149.095	43.24
	Galliformes	148.555	43.08
	Human	27.308	7.92
	Other	19.849	5.76
H7N3-Eurasian lineage	Anseriformes	224.138	60.61
	Galliformes	139.26	37.66
	Other	6.408	1.73
H7N3-American lineage	Anseriformes	299.621	57.67
	Environment	2.937	0.57
	Galliformes	177.599	34.18
	Other	39.43	7.59
H7N7-Eurasian lineage	Anseriformes	355.89	74.13
	Environment	23.605	4.92
	Galliformes	66.035	13.75
	Human	0.654	0.14
	Other	33.901	7.06
H7N9-Eurasian lineage	Anseriformes	135.309	36.93
	Environment	19.199	5.24
	Galliformes	95.244	26.00
	Human	103.006	28.11
	Other	13.628	3.72
H7N9-American lineage	Anseriformes	33.636	62.21
	Environment	0.943	1.74
	Galliformes	8.657	16.01
	Human	2.64	4.88
	Other	8.193	15.15

Supplementary Table 7. The Markov jumps for H7 and subtypes by host (Wild and Domestic).

Subtype	Host Transition			
	Host (from)	Host (to)	Markov Jumps	Percentage (%)
H7-Eurasian lineage	Environment	Domestic	2.091	1.49
	Environment	Human	0.003	0.00
	Environment	Other	0.954	0.68
	Environment	Wild	2.766	1.96
	Domestic	Environment	13.969	9.92
	Domestic	Human	4.622	3.28
	Domestic	Other	4.279	3.04
	Domestic	Wild	37.344	26.53
	Human	Environment	0.003	0.00
	Human	Domestic	8.301	5.90
	Human	Other	0.036	0.03
	Human	Wild	0.004	0.00
	Other	Environment	0.861	0.61
	Other	Domestic	1.005	0.71
	Other	Human	0.004	0.00
	Other	Wild	3.635	2.58
	Wild	Environment	8.514	6.05
	Wild	Domestic	43.941	31.21
	Wild	Human	0.003	0.00
	Wild	Other	8.439	5.99
H7-American lineage	Domestic	Human	3.013	11.15
	Domestic	Other	1.284	4.75
	Domestic	Wild	6.631	24.54
	hman	Domestic	0.423	1.57
	Human	Other	0.016	0.06
	hman	Wild	0.018	0.06
	Other	Domestic	0.175	0.65
	Other	Human	0.025	0.09
	Other	Wild	0.092	0.34
	Wild	Domestic	14.453	53.49
	Wild	Human	0.047	0.17
	Wild	Other	0.846	3.13
H7N3-Eurasian lineage	Domestic	Wild	7.249	35.49
	Wild	Domestic	13.178	64.51
H7N3-American lineage	Environment	Domestic	0.036	0.32
	Environment	Wild	0.077	0.69
	Domestic	Environment	0.152	1.37
	Domestic	Wild	1.870	16.88
	Wild	Environment	0.959	8.66
	Wild	Domestic	7.983	72.07
H7N7-Eurasian lineage	Environment	Domestic	3.288	3.01

	Environment	Human	0.001	0.00
	Environment	Other	1.290	1.18
	Environment	Wild	0.084	0.08
	Domestic	Environment	16.598	15.22
	Domestic	Human	2.106	1.93
	Domestic	Other	4.308	3.95
	Domestic	Wild	30.756	28.20
	Human	Environment	0.001	0.00
	Human	Domestic	0.020	0.02
	Human	Other	0.001	0.00
	Human	Wild	0.001	0.00
	Other	Environment	0.634	0.58
	Other	Domestic	0.541	0.50
	Other	Human	0.001	0.00
	Other	Wild	3.141	2.88
	Wild	Environment	0.226	0.21
	Wild	Domestic	36.802	33.74
	Wild	Human	0.002	0.00
	Wild	Other	9.262	8.49
H7N9-Eurasian lineage	Environment	Domestic	1.648	1.63
	Environment	Human	3.690	3.65
	Environment	Other	0.760	0.75
	Environment	Wild	1.127	1.12
	Domestic	Environment	4.224	4.18
	Domestic	Human	17.976	17.79
	Domestic	Other	0.062	0.06
	Domestic	Wild	2.021	2.00
	Human	Environment	18.554	18.37
	Human	Domestic	37.956	37.57
	Human	Other	0.164	0.16
	Human	Wild	1.824	1.81
	Other	Environment	0.012	0.01
	Other	Domestic	0.006	0.01
	Other	Human	0.005	0.00
	Other	Wild	0.008	0.01
	Wild	Environment	3.455	3.42
	Wild	Domestic	6.262	6.20
	Wild	Human	1.235	1.22
	Wild	Other	0.038	0.04
H7N9-American lineage	Environment	Human	0.158	1.82
	Environment	Domestic	0.032	0.37
	Environment	Wild	0.124	1.43
	Human	Environment	0.060	0.70
	Human	Domestic	0.044	0.50

Human	Wild	0.059	0.68
Domestic	Environment	0.171	1.97
Domestic	Human	0.431	4.98
Domestic	Wild	1.916	22.12
Wild	Environment	1.062	12.26
Wild	Human	0.554	6.40
Wild	Domestic	4.051	46.77

Supplementary Table 8. The Markov rewards for H7 and subtypes by host (Wild and Domestic).

Subtype	Host	Reward Time (year)	Percentage (%)
H7-Eurasian lineage	Environment	42.326	4.86
	Domestic	330.607	37.94
	Human	53.887	6.18
	Other	105.758	12.14
	Wild	338.797	38.88
H7-American lineage	Domestic	119.238	34.45
	Human	24.638	7.12
	Other	3.753	1.08
	Wild	198.529	57.35
H7N3-Eurasian lineage	Domestic	236.546	64.89
	Wild	127.976	35.11
H7N3-American lineage	Environment	2.154	0.47
	Domestic	163.181	35.52
	Wild	294.104	64.01
H7N7-Eurasian lineage	Environment	25.248	5.13
	Domestic	196.911	40.03
	Human	0.648	0.13
	Other	22.226	4.52
	Wild	246.891	50.19
H7N9-Eurasian lineage	Environment	22.996	6.14
	Domestic	127.272	33.99
	Human	106.318	28.39
	Other	2.78	0.74
	Wild	115.088	30.73
H7N9-Eurasian lineage	Environment	0.872	1.54
	Human	2.856	5.04
	Domestic	7.193	12.70
	Wild	45.719	80.72

Supplementary Table 9. The comparison of the weighted branch dispersal velocity (median value, 95% HPD).

Subtype	Anseriformes	Galliformes	*W*	*P*	Wild	Domestic	*W*	*P*
H7-Eurasian lineage	586.51 (518.91, 682.14)	294.44 (253.42, 343.79)	10000	< 2.2e-16	654.60 (595.14, 751.59)	581.32 (478.06, 653.14)	1016	< 2.2e-16
H7-American lineage	189.66 (112.79, 929.07)	8.34 (2.29, 19.94)	10000	< 2.2e-16	408.41 (304.88, 511.47)	172.85 (87.74, 513.66)	167	0.0005459
H7N3-Eurasian lineage	594.388 (516.63, 691.75)	2.94 (1.30, 10.37)	10000	< 2.2e-16	677.24 (560.01, 805.16)	43.02 (22.82, 305.56)	0	< 2.2e-16
H7N3-American lineage	1561.18 (1293.88, 1881.26)	3.15 (1.46, 5.91)	10000	< 2.2e-16	1602.18 (1274.80, 1859.94)	3.70 (2.23, 9.42)	0	< 2.2e-16
H7N7-Eurasian lineage	621.22 (503.67, 707.20)	346.68 (271.21, 429.61)	9999	< 2.2e-16	507.47 (416.94, 609.94)	766.87 (631.36, 943.98)	9982	< 2.2e-16
H7N9-Eurasian lineage	338.39 (205.39, 497.05)	12.10 (7.49, 17.44)	10000	< 2.2e-16	275.37 (105.04, 451.31)	56.62 (34.74, 122.20)	87	< 2.2e-16
H7N9-Eurasian lineage	1444.47 (201.08, 2198.11)	4.36 (2.68, 6.51)	10000	< 2.2e-16	296.41 (54.74, 760.83)	4.90 (3.05, 7.52)	0	< 2.2e-16

Note: Statistical significance was assessed using the two-sided Mann-Whitney U test (Wilcoxon rank-sum test), as the data did not meet the assumption of normality (Shapiro–Wilk test, $p < 0.05$ for at least one group). No adjustment was made for multiple comparisons across subtypes. Test statistics (W) and exact p-values are reported. p-values are displayed as "< 2.2e-16" due to computational precision limits in R. All analyses were based on independent virus isolates.

Supplementary Table 10. The weighted branch dispersal velocity of high and low pathogenic H7 viruses and subtypes in different hosts (median value, 95% HPD).

Subtype	Anseriformes	Galliformes	Wild	Domestic
H7-HPAI	-	175.42 (129.97, 211.20)	274.63 (144.90, 1121.37)	196.67 (153.66, 256.48)
H7-LPAI	651.40 (581.88, 719.04)	156.14 (115.70, 199.84)	465.09 (122, 655.54)	540.60 (474.40, 610.32)
H7N3-HPAI	-	33.13 (18.49, 54.49)	-	37.30 (21.77, 55.83)
H7N3-LPAI	497.21 (430.18, 569.87)	77.06 (21.23, 126.09)	494.84 (413.20, 574.23)	282.04 (218.76, 376.17)
H7N7-HPAI	-	353.08 (279.38, 429.86)	-	364.20 (278.78, 430.15)
H7N7-LPAI	600.54 (508.59, 691.17)	144.85 (104.33, 212.12)	471.99 (395.99, 567.30)	774.76 (652.39, 860.32)
H7N9-HPAI	-	330.54 (252.64, 425.51)	-	290.48 (194.47, 396.10)
H7N9-LPAI	425.46 (313.98, 529.27)	247.20 (145.30, 349.14)	358.86 (263.01, 491.65)	335.67 (240.19, 461.93)

Note: HPAI, high-pathogenic avian influenza; LPAI, low-pathogenic avian influenza. "- " indicates insufficient data for calculation.
