# Supplementary material for: Disentangling the drivers and host-mediated global spread of H7 influenza A virus
Source: Nat Commun. 2026 May 6;17:6076. doi: 10.1038/s41467-026-72718-9 (PMC13351079; doi:10.1038/s41467-026-72718-9)
Supplement: Supplementary file 3 — Reporting Summary [file 41467_2026_72718_MOESM3_ESM.pdf]

nature portfolio

Corresponding author(s): Shigui Yang

Last updated by author(s): Apr 18, 2026

Reporting Summary

Nature Portfolio wishes to improve the reproducibility of the work that we publish. This form provides structure for consistency and transparency in reporting. For further information on Nature Portfolio policies, see our Editorial Policies and the Editorial Policy Checklist.

Statistics

For all statistical analyses, confirm that the following items are present in the figure legend, table legend, main text, or Methods section.

n/a	Confirmed	
☐	☒	The exact sample size (*n*) for each experimental group/condition, given as a discrete number and unit of measurement
☐	☒	A statement on whether measurements were taken from distinct samples or whether the same sample was measured repeatedly
☐	☒	The statistical test(s) used AND whether they are one- or two-sided<br>*Only common tests should be described solely by name; describe more complex techniques in the Methods section.*
☐	☒	A description of all covariates tested
☐	☒	A description of any assumptions or corrections, such as tests of normality and adjustment for multiple comparisons
☐	☒	A full description of the statistical parameters including central tendency (e.g. means) or other basic estimates (e.g. regression coefficient) AND variation (e.g. standard deviation) or associated estimates of uncertainty (e.g. confidence intervals)
☐	☒	For null hypothesis testing, the test statistic (e.g. *F*, *t*, *r*) with confidence intervals, effect sizes, degrees of freedom and *P* value noted<br>*Give P values as exact values whenever suitable.*
☐	☒	For Bayesian analysis, information on the choice of priors and Markov chain Monte Carlo settings
☐	☒	For hierarchical and complex designs, identification of the appropriate level for tests and full reporting of outcomes
☐	☒	Estimates of effect sizes (e.g. Cohen's *d*, Pearson's *r*), indicating how they were calculated

Our web collection on statistics for biologists contains articles on many of the points above.

Software and code

Policy information about availability of computer code

Data collection	No software was used for data collection. Code and software used to process sequence-data generated in this study are provided in the Methods.
Data analysis	All software tools used for the data analysis are open-source unless otherwise stated. Sequence alignments were performed with MAFFFT v7.526 using AliView v1.28 and trimmed using trimAL v1.4. Temporal signal and phylogenetic analyses were performed using TreeTime v0.11.3, IQ-TREE v2.0.7 and BEAST v1.10.4. Tracer v1.7.2 was used to visualise and analyse MCMC trace files from Bayesian phylogenetic analysis. The date of each sequence was converted to a decimal format using BioAider v1.5.2. SpreaD3 v0.9.7 was used to estimate Bayes factor (BF). TreeAnnotator v1.10.4, FigTree v1.4.4 and Chiplot (a website tool) were used to visualise the trees. The R package 'seraphim' and 'diagram' (R 4.4.4) were used to estimate and visualize the results of the continuous phylogeographic analysis. All the code and software used for data analysis will be available in the data repository associated with the manuscript at https://doi.org/10.5281/zenodo.19239968.

For manuscripts utilizing custom algorithms or software that are central to the research but not yet described in published literature, software must be made available to editors and reviewers. We strongly encourage code deposition in a community repository (e.g. GitHub). See the Nature Portfolio guidelines for submitting code & software for further information.

Data

Policy information about availability of data

All manuscripts must include a data availability statement. This statement should provide the following information, where applicable:

- Accession codes, unique identifiers, or web links for publicly available datasets
- A description of any restrictions on data availability
- For clinical datasets or third party data, please ensure that the statement adheres to our policy

The genome sequences and associated metadata used in this study are available in Global Initiative on Sharing All Influenza Data (GISAID) (https://platform.epicov.org) and NCBI influenza Virus Resource (https://www.ncbi.clm.gov/geomes/FLU/) databases.
Avian hosts were classified as Anseriformes and Galiformes, as wel as wild and domestic birds using strain names, associated metadata, and original publication.
Outbreaks in birds worldwide were obtained from World Animal Heath ihformation system (WAHIS), World Organisation for Animal Heath (https://wahis.woah.org/), and EMPREs-i+ Global Animal isease information System, Food and Agricuture Organisation (https://empres-i.apps.fao.org). All reported and confirmed infections of H7 virus inhumans were obtained from WHO (httos://www.who.int/emergencies/disease-outbreak-news).
A comprehensive list of GenBank and GISAID accession identifiers is provided in the data/Supplementary Data.xlsx file of the GitHub repository (http://github.com/skylynf/H7_Dynamics), and archived at Zenodo with the http://doi.org/10.5281/zenodo.19239968.

Research involving human participants, their data, or biological material

Policy information about studies with human participants or human data. See also policy information about sex, gender (identity/presentation), and sexual orientation and race, ethnicity and racism.

Reporting on sex and gender	N/A
Reporting on race, ethnicity, or other socially relevant groupings	N/A
Population characteristics	N/A
Recruitment	N/A
Ethics oversight	N/A

Note that full information on the approval of the study protocol must also be provided in the manuscript.

Field-specific reporting

Please select the one below that is the best fit for your research. If you are not sure, read the appropriate sections before making your selection.

☒ Life sciences ☐ Behavioural & social sciences ☐ Ecological, evolutionary & environmental sciences

For a reference copy of the document with all sections, see nature.com/documents/nr-reporting-summary-flat.pdf

Life sciences study design

All studies must disclose on these points even when the disclosure is negative.

Sample size	In this study,no sample size calculations were performed; however, all available H7 avian influenza sequences in GenBank and GISIAD were used.
Data exclusions	For phylogenetic analysis,duplicate sequences,laboratory-derived and mixed subtype isolates were excluded.
Replication	All raw public datasets used in this study are fully documented with open access links at https://doi.org/10.5281/zenodo.19239968. A comprehensive list of GenBank and GISAID accession identifiers is provided in the data/Supplementary Data.xlsx file of the GitHub repository (https://github.com/skylynf/H7_Dynamics), and archived at Zenodo with the https://doi.org/10.5281/zenodo.19239968. For all phylogenetic analysis, a minimum of two independent runs were performed, and their convergence and effective sample sizes were confirmed to be adequate prior to inference.
Randomization	Randomization was not required for this study as it contained no experimental group or comparisons.
Blinding	Blinding was not relevant as publicly available surveillance data, with de-identified patient data, were utilised in the study.

Reporting for specific materials, systems and methods

We require information from authors about some types of materials, experimental systems and methods used in many studies. Here, indicate whether each material, system or method listed is relevant to your study. If you are not sure if a list item applies to your research, read the appropriate section before selecting a response.

Materials & experimental systems

n/a	Involved in the study
☒	☐ Antibodies
☒	☐ Eukaryotic cell lines
☒	☐ Palaeontology and archaeology
☒	☐ Animals and other organisms
☒	☐ Clinical data
☒	☐ Dual use research of concern
☒	☐ Plants

Methods

n/a	Involved in the study
☒	☐ ChIP-seq
☒	☐ Flow cytometry
☒	☐ MRI-based neuroimaging

Plants

Seed stocks	*Report on the source of all seed stocks or other plant material used. If applicable, state the seed stock centre and catalogue number. If plant specimens were collected from the field, describe the collection location, date and sampling procedures.*
Novel plant genotypes	*Describe the methods by which all novel plant genotypes were produced. This includes those generated by transgenic approaches, gene editing, chemical/radiation-based mutagenesis and hybridization. For transgenic lines, describe the transformation method, the number of independent lines analyzed and the generation upon which experiments were performed. For gene-edited lines, describe the editor used, the endogenous sequence targeted for editing, the targeting guide RNA sequence (if applicable) and how the editor was applied.*
Authentication	*Describe any authentication procedures for each seed stock used or novel genotype generated. Describe any experiments used to assess the effect of a mutation and, where applicable, how potential secondary effects (e.g. second site T-DNA insertions, mosiacism, off-target gene editing) were examined.*
