# Supplementary material for: Disentangling the drivers and host-mediated global spread of H7 influenza A virus
Source: Nat Commun. 2026 May 6;17:6076. doi: 10.1038/s41467-026-72718-9 (PMC13351079; doi:10.1038/s41467-026-72718-9)
Supplement: Supplementary file 2 — Peer Review file [file 41467_2026_72718_MOESM2_ESM.pdf]

nature portfolio

Peer Review File

Disentangling the Drivers and Host-Mediated Global Spread of Influenza A (H7) Virus

Corresponding Author: Professor Shi-Gui Yang

This file contains all reviewer reports in order by version, followed by all author rebuttals in order by version.

Version 0:

Reviewer comments:

Reviewer #1

(Remarks to the Author)
In this study, the authors present a comprehensive and well-executed phylogenetic analysis of the H7 avian influenza virus on a global scale, aiming to elucidate its evolutionary dynamics, spatial transmission patterns, host-specific roles, and factors influencing its spread. By employing cutting-edge Bayesian phylogenetic methods, the authors systematically and comprehensively consider the viral persistence, cross-regional transitions, and cross-host spillover of the H7 avian influenza virus. Their findings suggest that Asia serves as the primary evolutionary hub for the H7 virus, with phylogeographic evidence supporting ongoing lineage diversification. Additionally, the study identifies Anseriformes and wild birds as likely key hosts facilitating the cross-continental transmission of the H7 virus.
In summary, this study holds significant scientific value, as these findings provide crucial evidence for future H7 surveillance, early warning systems, and preparedness for potential pandemics. However, there are still some issues here that need to be addressed:

Major comments:
The author investigated the transmission speed of viruses in four different hosts separately. The results suggested H7 viruses spread faster among Anseriformes and wild birds. However, I wonder if the transmission speed would be for different among Anseriformes-wild, Anseriformes-domestic, Galliformes-wild, and Galliformes-domestic. To comprehensively assess transmission dynamics, we recommend further work to quantify spread velocities across host interface combinations.

Minor comments:
1.Line 39-40 The sentence "which resulted in over 1,687 cases caused by H7N9" in the Introduction is inconsistent with the data of "with a total of 1564 human infections" in Line 410 (Discussion section). Please proofread and unify them as well as add and verify the references.
2.Line 140 What does BSSVS mean? It seems that the entire text does not provide any explanation for it.
3.Line 146 What does Markov reward mean? I saw the legend in Figure 3 explaining it, so what are the time units in years, months, and days. Or other units of time?
4.Lines 263-264 "A Bayesian phylogeographic GLM was used to reconstruct the spatiotemporal dynamics of H7 viruses and assess potential predictors of spatial diffusion", Please explain in detail the Bayesian phylogenetic GLM method. Can the GLM method reconstruct the spatiotemporal dynamics of H7? Or if the author's expression is incorrect and clear, the GLM method should be used to evaluate the impact of spatial diffusion.
5.Lines 265-266 The sentence "showing a strong negative association with transmission (coefficient = -1.015, 95% CI: -1.173 to -0.879)", Please verify whether it should be 95% CI or 95% highest posterior density (HPD), as the Line 270 shows 95% HPD.
6.Lines 291-292 What does the sentence "The number of triangles represents the order of the number of outbreaks" mean? Please reorganize the language.
7.Lines 293-294 The arrows of different colors represent the transmission routes of different subtypes. Do they spread in a straight line along the direction of the arrows, and how do they spread?
8.Line 346 There are occasional language errors throughout the entire text, such as "A large fraction of poultry products are traded" should be changed to "A large fraction of poultry products is traded". Please read and polish thoroughly.
9.Lines 421-424 The discussion of the biological mechanisms that negatively correlate with temperature in the existing text is somewhat vague. I suggest adding 1-2 sentences to clarify the significance of this result for the spread of avian influenza in the context of climate change.

10.Lines 415-444 This paragraph of discussion has too many words, which affects readers' reading and understanding. It is recommended to have a segmented and targeted discussion.
11.Lines 458-475 The conclusion is a bit lengthy, please condense it.
12.Line 471-472 The One Health-oriented strategy needs more depth. Rather than providing actionable recommendations, it just mentions high-risk areas and targeted surveillance in a general way. While the results find important phenomenon such as there have been a relatively high number of H7 virus outbreaks in Africa in recent years, the author does not offer discussion how this finding should enlighten specific policies. For example, the authors may propose directing resources to regions severely affected by avian influenza.
13.Line 473 The conclusion advocates for targeted monitoring in high-risk areas, but lacks description of specific regions. The research findings should be used to define 'high-risk' regions/hosts (e.g. prioritizing the attention of Anseriformes on the migration routes of East Asia or live poultry trading centers in Asia).
14.Line 575 There is an extra space after the bracket, Please carefully check the punctuation throughout the entire manuscript.
15.Given complexity of the methods in this study, I wonder whether a pipeline added in Methods could help clarify this.

(Remarks on code availability)

Reviewer #2

(Remarks to the Author)
This paper presents an analysis of the spread of avian influenza H7 viruses using phylodynamic and phylogeographic techniques on publicly accessible sequence data from 2000 to 2024 (present). The analysis is split into subtypes, the transmission patterns between host species and spatial dispersion patterns are analysed.

The methods used for the analyses are generally good and well described, and mirrors what has already been done for H5 (highly pathogenic). However, I have a few detailed comments which will hopefully improve the study design, analysis and interpretation of results.

Introduction

Please check the wording of the first sentence, references 1 and 2 relate to H5 (& H13 / H16) viruses, only reference 3 mentions H7. Suggest remove ‘particularly those of the H7 subtype’.

I think you need to mention low and high pathogenicity types for H5 and H7 in the introduction; this is important because most of the recent H5 spread studies have been on high pathogenicity (and specific clades of H5) types, whereas you are considering both low and high pathogenicity types of H7 - you don’t distinguish between them, and do not explain why you chose to consider them together.

Results

Besides the data in figure 1 (cases, sequences, hosts), since the methods section of this paper is at the back, please include a very brief description of what the dataset for phylogenetic analysis is in the main text (noted that the details are in the supplementary). Something along the lines of ‘We complied four data sets of segment 4 (coding for the hemagglutinin surface protein) sequences from 2000-2024, from A: a sample of all hosts and H7 subtypes; B: H7N3 only; etc.. after subsampling these ranged in size from X-Y, please see methods [etc] for details’. This needs to come before, or at the start of the ‘Spatial diffusion of H7 virus section’

Page 5 - suggest “Supplementary figure 3 shows a relatively even spatiotemporal distribution of sequences, which could support”

Page 5 - The use of ‘widespread’ in this sentence is misleading - suggest removing it
[and/or something like this - “Multiple local spill over events of H7N9 from poultry to humans were observed between 2013 and 2017, and the inferred genetic diversity (Ne) showed a declining trend. This decrease may be attributed to..”]

Page 7 - Spatial diffusion of H7 virus
It is not clear from the text as it currently is what the four datasets are; specifically you need to put in the main text that the ‘H7’ dataset contains all the subtypes. Aside question - why did you not exclude the sequences with only H7 subtype listed and no NA type ?

Figure 2 - maybe add a bar for subtype and continent to all sub-figures (so they all have 3 bars) ?
Also, can you indicate which are highly pathogenic ? (Another bar ?).

Page 9 / Figure 3 - considering that the H7N3, H7N7, H7N9 subtypes do not form monophyletic clades (of only those subtypes) in the H7 (all subtypes) tree - is it valid to calculate Markov rewards in those subtypes ? (I am suggesting that it is not).

Pages 10-13 - for the more detailed continuous trait phylogeography; especially per subtype - suggest that you analyse individual monophyletic clades (initial outbreaks + spread) separately, and not mix geographically and genetically

unconnected outbreaks. Just because the HA H7's are paired with the same subtype numbered NA does not mean that the viruses are particularly closely related. The same idea of not mixing geographically and genetically unconnected outbreaks also applies for the Host transmission analysis on pages 12 and 13.

Page 13 - regarding observation of faster spread in one species than another - is this also geographically segregated ? Or are the dispersal velocities the same in different continents ? And what about dispersal of high vs low pathogenicity viruses ? Would you expect to see slow spread of high pathogenicity H7 amongst galliformes in a restricted geographic area ? (Suggesting that this should be detectable).

Page 16 - H7N9 Human sequences; I think the way you have analysed and described these is very misleading. There is was not widespread human to human transmission of H7N9, the majority of the human cases were from poultry to human spill over events. It is only biased sampling of human cases, and under sampling of the transmitting birds which makes the trees look like there is persistence in humans. Suggest you remove this paragraph and concentrate on the avian species.

Page 20 - "In addition the long term presence of H7N9 in Asia may be related to its ability to adapt to poultry hosts.." - do the recent H7N9 sequences have the characteristic mutations for alpha 2,6 rather than alpha 2,3 ? (Reference 18 is about 1918 virus, not H7N9 mutations, or do you mean reference 19 - but that is about North America).

Page 22 - why would 'long term adaption within the host may enhance the potential for cross species transmission' ? If the virus is well adapted to a host species, why does would that mean it could easily adapt to a different species (surely it would be less compatible rather than more).
Also, as above - what are those mutations which allow partial binding to alpha 2,6 ?

Page 24 - presumably it was human mediated activity such as trade that accelerated the geographic spread of the virus, not humans as hosts themselves ? Suggest that you re-write this part.

Page 25 - correlated predictors in GLM - can you pre-select the least correlated set of variables and just run with those rather than all of them ?

Supplementary Figure 6 and similar - please add the dataset labels for A, B, C, D in the panels and figure legend.

(Remarks on code availability)
The authors will make the datasets available on Zenodo but havent done so yet.

Reviewer #3

(Remarks to the Author)
In the paper "Disentangling the Drivers and Host-Mediated Global Spread of Influenza A (H7) Virus" the authors used Bayesian phylodynamic analysis to explore geographic dispersal of H7 and highlight the subtypes H7N3, H7N7, and H7N9 over time, along with exploring how host animal interacted with dispersal dynamics. We appreciated the cohesive and focused narrative throughout the results - which used appropriate methods to address relevant questions. Overall, this was an interesting paper with important implications. We have a few primarily concerns that we would like to see addressed.

Major concerns
1. Wave front distance shown in top panels of Fig 7 is currently presented in a manner that is somewhat difficult to reconcile for the reader because the majority of travel occurs prior to the shown timeline. We feel that further discussion and interpretation would be beneficial for the reader to understand. Supplemental figures 8-11E and F provide meaningful context to Figure 7 and we would suggest incorporating them in the main text. This could be tied into earlier geographical divergence results (ex. line 119). Alternatively, Fig 7 could be reconfigured to present the final wave front distance, and not introduce the element of time, reflecting the current discussion in lines 238-260.
2. The data and xmls have not been made available for review, that we have found.

Minor concerns
1. For most figures it would be helpful to have the panels visually labeled in the figure (as was done fig 5), rather than only in the legend (as was done fig 2, 3, 4, 6, 7).
2. Line 48 Galliformes are not introduced as was done for Anseriformes.
3. Figure 1a has some ambiguity in the figure legends. It is unclear if the line plot or the bar plot refers to the left or right axis using only information in the figure/legend. Additionally with the density of the information it might be helpful to 1) separate the bar and line plot (allowing the authors to color code the lines as well as indicate by shape) and 2) to log scale the y axis. Although we will note we appreciate that vertically aligning the x-axis timeline is helpful for readability.
4. Supp Figures 5. and 6. do not specify which panel is associated with each subtype.
5. Line 528/Supp Figure 5, what are the time intervals used with estimating Ne?
6. Line 111-113 is not a complete sentence.
7. Fig 2. It is unclear why the deeper internal branches are not colored. Was there a threshold for support, if so that should be discussed.
8. Line 120-125. Including the uncertainty in the time estimates of divergence events would be informative.
9. Figure 5. The direction arrow heads are indistinct and can be easily mistaken as dots, making them fully opaque or

outlined could improve readability.
10. Line 200. It initially appears that these are a separate category with non-overlapping samples from the Galliformes and Anseriformes. Introducing that all samples from bird hosts were partitioned into wild vs domestic here would improve readability.
11. Line 210. Was there a statistical test done to show statistical significance of the difference?
12. Line 265. The word "barrier" and phrase "positive contribution to the virus spread" implies some level of inferred causality, which has not been shown. "Correlated" and "associated" do not have this issue.
13. At lines 267 and 268-9 defining chicken stock and virus sample size, respectively could improve clarity of the text.
14. Figure 9. A visual color legend in the figure, instead of only describing in the legend text, would improve interpretability. It would also be helpful to use referential color schemes for the subtypes and outbreaks (eg. If H7N9 is red then use light and dark red arrows for 2013/2014 and 2016/2017 and blue or orange for 2023). Overall figure 9 is a helpful, elegant summary figure.
15. Line 507-509 there is a lack of detail in this process, was there a threshold used, how many sequences were removed?
16. Line 551. A supplementary table that specified what indicated domestic vs wild would improve the reproducibility of the paper, but we would not require it.

(Remarks on code availability)
We have not been able to find any code. The authors say the data XMLs have been uploaded to zenodo and will be available upon publication, however we have not seen them, nor been able to reproduce the analyses. Data and reproducible analyses must be made available for further review.

Reviewer #4

(Remarks to the Author)
I co-reviewed this manuscript with one of the reviewers who provided the listed reports. This is part of the Nature Communications initiative to facilitate training in peer review and to provide appropriate recognition for Early Career Researchers who co-review manuscripts.

(Remarks on code availability)

Version 1:

Reviewer comments:

Reviewer #1

(Remarks to the Author)
I would like to express my appreciation to the authors for their thoughtful and comprehensive responses to the my comments, which have effectively addressed all of my questions.
The revised manuscript has been substantially improved, demonstrating significant enhancements in methodological rigor, analytical robustness, and overall clarity, with conclusions reasonably supported by the presented evidence. The findings represent a valuable contribution, with formative insights that critically inform the development of robust public health readiness and operational response protocols for avian influenza pandemics.
I recommend its acceptance for publication.

(Remarks on code availability)

Reviewer #2

(Remarks to the Author)
Many thanks for considering and replying to my comments of the first round. I think your revised version now has much improved analysis and interpretation; and you have addressed the points I raised sufficiently well. I think the new analyses splitting by host type and hpai/lpai is much better and shows more clearly the details of the H7's transmission patterns.

1. New minor points:
However, there are now a couple of new minor points, to reword some sentences, which require attention -

Page 2 Lines 37-47 "Historically, outbreaks caused by high-pathogenic avian influenza (HPAI) viruses have mostly arisen from virulence shifts by low-pathogenic avian influenza (LPAI) viruses" - This is true, but I think it might be misinterpreted, for example the recent H5 clade 2.3.4.4b epizootic outbreaks, although 'historically' these did arise from a LPAI to HPAI event in 1996 (Gs/Gd Lineage), they are not really referred to as a single outbreak now since the H5 2.3.4.4b viruses are widespread amongst continents, countries, species and years (2014 onwards). So suggest re-wording to talk about origin of clades or lineages ? perhaps
"Historically, the major high-pathogenic avian influenza (HPAI) virus lineages are known to have originated from previously low-pathogenic avian influenza (LPAI) strains" [give reference to a review paper, e.g. your reference 6].

"Notably, HPAI H5 virus has demonstrated unprecedented pandemic potential, by becoming endemic in bird reservoirs and causing widespread infections across 84 countries in Asia, Africa, Europe, and North America (ref 6)" - possibly true but misleading as written; pandemic potential usually refers to human pandemic potiential, so it is unclear how 'endemic in wild bird reservoirs' would cause this. I think you are meaning to refer to the recent H5 2.3.4.4b epizootic here, so I think it would be better to specific. Suggest something more like:
"Notably, a major lineage of HPAI H5, clade 2.3.4.4b viruses, are the cause of an epizootic in wild bird reservoirs with widespread infections across 84 countries in Asia, Africa, Europe, and North America (ref 6)" [suggest including some other references from your list also].

This sentence is also problematic - (part 1)
"The mechanisms by which LPAI viruses of the H5 and H7 subtypes evolve into HPAI viruses involve mutations, insertions, and recombination events occurring at the proteolytic cleavage site of the hemagglutinin (HA) protein" - really ? I thought the main mechanism was acquisition of a multi basic cleavage site between HA1 and HA2, which is mostly insertion rather than mutation or recombination. Please give references for the H5 and H7 mechanisms.

(part 2) "playing a critical role in mediating the virus's attachment to host cell receptors, thereby initiating the viral infection cycle (ref 6)". This a bit misleading, this part of the sentence makes it sound like the cleavage site is the same as the receptor binding site, which it is not. (And note that in the HA 2.3.4.4b the receptor binding site is avian-like, not human-like, hence the issue with the earlier sentence on 'pandemic potential'...)

[Page 3, Lines 60-62]
"However, the roles of different host groups in the global dissemination and cross-species transmission of H7 subtypes remain poorly understood, and it is still unclear how fast the LPAI and HPAI H7 viruses spread among diverse host populations" - great to analyse the spread in different host groups, but suggest that you re-word this sentence to be positive.

[Page 17, Lines 366-381]
"The differential dispersal velocities between LPAI and LPAI" - typo, one of the LPAI should be HPAI

2: About the revised analysis and figure changes - thanks for considering my comments, and I'm pleased that you were able to improve the manuscript, all these changes look good and answer my original points. Specifically -

Response 7: many thanks for including the continent information and alternations to the figures
Response 8: good, thanks for updating the analysis
Response 9: (analysis of individual monophyletic clades) - excellent thanks for doing this, and I'm glad that it helped the overall analysis and interpretation of what is happening.
Response 10: (faster dispersal in one host-type / geographic region than another) - excellent that you can split these effects out, and thanks for the additional comments in the discussion

(Remarks on code availability)
The data sets and scripts/code are well described in this repository, including readme and installation instructions, many thanks for making this available.

Reviewer #3

(Remarks to the Author)
The authors have addressed some of our concerns, however there are a few remaining points of interest that should be addressed:

Major concerns

With respect to Reviewer #3 Response 1: We feel that the authors have not addressed the concern about showing the full temporal dynamics of the wavefront. The authors state in their response "we agree that the results from original Supplemental Figures 8-11E and F provide important context that enhances the interpretation of original Figure 7. Accordingly, these figures have been incorporated into the main text" however, the key information in those figures (the time period in which the wave front expands) has not actually been incorporated into the main text and been removed from the supplement.

Regarding Response 7 there is missing analysis to support some of the discussed results about the deeper phylogeographic history in the manuscript. Line 28 and Line 112-113: there are currently no inferences shown in figures or tables that support Asia as the source H7; and the inference of geographic divergence discussed in Line 119-127 is not actually shown in fig 2. Temp Fig 1 in response to reviewers shows the phylogeographic inference.

Minor concerns

Additionally, with respect to Reviewer #3 Response 1, the authors summarized the wavefront focusing on final distance in Fig 8a; a similar approach could be taken for Fig 8b, given that the temporal dynamics of the diffusion coefficient are also not

addressed in the text.

With respect to the changes made in Response 6, in the updated manuscript Line 112, the phrase “unidirectional evolution” is unclear, “unidirectional transmissions events” would be understood.

(Remarks on code availability)

Reviewer #4

(Remarks to the Author)
I co-reviewed this manuscript with one of the reviewers who provided the listed reports. This is part of the Nature Communications initiative to facilitate training in peer review and to provide appropriate recognition for Early Career Researchers who co-review manuscripts.

(Remarks on code availability)

Version 2:

Reviewer comments:

Reviewer #3

(Remarks to the Author)
The authors have satisfied our concerns.

(Remarks on code availability)

Reviewer #4

(Remarks to the Author)
I co-reviewed this manuscript with one of the reviewers who provided the listed reports. This is part of the Nature Communications initiative to facilitate training in peer review and to provide appropriate recognition for Early Career Researchers who co-review manuscripts.

(Remarks on code availability)

Open Access This Peer Review File is licensed under a Creative Commons Attribution 4.0 International License, which

permits use, sharing, adaptation, distribution and reproduction in any medium or format, as long as you give appropriate credit to the original author(s) and the source, provide a link to the Creative Commons license, and indicate if changes were made.
In cases where reviewers are anonymous, credit should be given to 'Anonymous Referee' and the source.
The images or other third party material in this Peer Review File are included in the article's Creative Commons license, unless indicated otherwise in a credit line to the material. If material is not included in the article's Creative Commons license and your intended use is not permitted by statutory regulation or exceeds the permitted use, you will need to obtain permission directly from the copyright holder.
To view a copy of this license, visit https://creativecommons.org/licenses/by/4.0/

Point-by-point responses to the reviewers' comments:

Reviewer #1:

In this study, the authors present a comprehensive and well-executed phylogenetic analysis of the H7 avian influenza virus on a global scale, aiming to elucidate its evolutionary dynamics, spatial transmission patterns, host-specific roles, and factors influencing its spread. By employing cutting-edge Bayesian phylogenetic methods, the authors systematically and comprehensively consider the viral persistence, cross-regional transitions, and cross-host spillover of the H7 avian influenza virus. Their findings suggest that Asia serves as the primary evolutionary hub for the H7 virus, with phylogeographic evidence supporting ongoing lineage diversification. Additionally, the study identifies Anseriformes and wild birds as likely key hosts facilitating the cross-continental transmission of the H7 virus.

In summary, this study holds significant scientific value, as these findings provide crucial evidence for future H7 surveillance, early warning systems, and preparedness for potential pandemics. However, there are still some issues here that need to be addressed:

Response: We would like to express our sincere thanks to the reviewer for the review of our manuscript and for the insightful suggestions. We sincerely appreciate the reviewer's positive evaluation of our study's scientific value and methodological rigor, particularly the use of cutting-edge Bayesian phylogenetic approaches. At the same time, we have thoroughly revised the manuscript to address all the comments raised by the reviewer. Regarding every comment mentioned by the reviewer, we have also made extensive revisions to the manuscript to address them. Below, we provide a point-by-point response to each comment. These recommendations have fortified the robustness of our findings as well as enhanced the overall quality of our manuscript.

Major comments:

The author investigated the transmission speed of viruses in four different hosts separately. The results suggested H7 viruses spread faster among Anseriformes and wild birds. However, I wonder if the transmission speed would be for different among Anseriformes-wild, Anseriformes-domestic, Galliformes-wild, and Galliformes-domestic. To comprehensively assess transmission dynamics, we recommend further work to quantify spread velocities across host interface combinations.

Response:

Thank you for this excellent and insightful comment. We agree entirely that understanding transmission dynamics at the interface between different host types is crucial for a comprehensive assessment of viral spread risk, and we appreciate the suggestion to further explore the potential differences of transmission velocities in hosts cross-categorized not only by host order but also by domestication status.

To address this valuable point, we further analyzed dispersal velocities in different categorized hosts: Anseriformes-wild, Anseriformes-domestic, Galliformes-wild and Galliformes-domestic. The results revealed a clear hierarchy of dispersal velocities, with H7 virus spreading the fastest in the Anseriformes-wild populations. These new findings have been integrated into the Results section and are now discussed in depth in the Discussion section. We believe this will significantly strengthen our study.

Change in the text (the Results section):

[Page 10, Lines 213-217]

In addition, Anseriformes-wild populations exhibited the fastest dispersal velocity (median: 580.89 km/year), followed by Anseriformes-domestic populations (437.20 km/year). Galliformes-domestic populations showed moderate velocity (216.37 km/year), while Galliformes-wild populations displayed the slowest velocity (46.88 km/year) (Supplementary Fig.18). This result highlights Anseriformes-wild populations represent the most efficient drivers of long-range virus transmission.

Change in the text (the Discussion section):

[Page 17, Lines 356-365]

Our refined analysis of host interface combinations also offers novel insights into the mechanisms of H7 virus spread. The markedly high velocity observed in Anseriformes-wild populations may be likely attributable to the long-distance migratory behavior of wild waterfowl, which function as efficient natural vectors for viral dispersal across continents[9]. The intermediate velocity of Anseriformes-domestic populations likely reflects the role of domestic duck farming and trade, which facilitates regional spread but at a slower pace than natural migration. In contrast, the lower velocities among Galliformes-domestic and particularly Galliformes-wild populations may stem from the sedentary nature of many Galliformes species such as pheasants in confined habitats and

the absence of strong anthropogenic drivers like trade networks.

*[9]Olsen, B. et al. Global patterns of influenza a virus in wild birds. Science **312**, 384 –388 (2006).*

Minor comments:

1. Line 39-40 The sentence "which resulted in over 1,687 cases caused by H7N9" in the Introduction is inconsistent with the data of "with a total of 1564 human infections" in Line 410 (Discussion section). Please proofread and unify them as well as add and verify the references.

Response 1:

Thank you for your careful comment. After verification, we expressed in the Introduction section that 1,687 cases of human infection with H7 virus is the global statistics; while the 1564 cases mentioned in our Discussion section explicitly state that this is the number of human cases of H7N9 avian influenza in China between February 2013 and September 2017,.

To clarify this point and avoid misunderstandings, we have revised the sentences both in the Introduction and Discussion section, and references are also added to provide the basis for the data source.

Change in the text (the Introduction section):

[Page 2, Lines 36-37]

The H7 subtype has been responsible for several outbreaks in poultry and sporadic but severe human infections, most notably the H7N9 epidemic, resulting in in over 1,687 cases globally[4,5].

[4] Shi, J., Zeng, X., Cui, P., Yan, C. & Chen, H. Alarming situation of emerging H5 and H7 avian influenza and effective control strategies. Emerg Microbes Infect 12, 2155072 (2023).

[5] Feoktistova, S. G. et al. Phylogenetic Insights into H7Nx Influenza Viruses: Uncovering Reassortment Patterns and Geographic Variability. Viruses 16, (2024).

Change in the text (the Discussion section):

[Page 18, Lines 390-391]

However, H7N9 caused at least five waves in China during February 2013 and September 2017,

with a total of 1564 human infections[4].

[4] Shi, J., Zeng, X., Cui, P., Yan, C. & Chen, H. Alarming situation of emerging H5 and H7 avian influenza and effective control strategies. Emerg Microbes Infect 12, 2155072 (2023).

2. Line 140 What does BSSVS mean? It seems that the entire text does not provide any explanation for it.

Response 2:

We sincerely appreciate the reviewer's careful reading and valuable feedback. The term "BSSVS" refers to Bayesian Stochastic Search Variable Selection, a method for high-dimensional variable selection in Bayesian frameworks. Since we wrote the full name in the method section, which is located in the end of the full text. We acknowledge that its abbreviation was introduced without prior definition. To ensure readability, we have modified it now.

Change in the text (the Results section):

[Page 7, Lines 132-134]

the most strongly supported transition rates between discrete geographic locations were compiled using Bayesian stochastic search variable selection (BSSVS) and are summarized in Fig. 3.

Change in the text (the Methods section):

[Page 25, Lines 524-526]

Symmetric discrete-trait phylogeographic analyses (i.e., reversible CTMC model) with BSSVS were conducted in BEAST v.1.10.4 to determine a sparse set of transition rates that fully summarizes the epidemiological connectivity.

3. Line 146 What does Markov reward mean? I saw the legend in Figure 3 explaining it, so what are the time units in years, months, and days. Or other units of time?

Response 3:

We sincerely appreciate the reviewer's insightful question regarding the time units of the Markov

reward in Figure 3. The Markov reward was calculated based on annual units (years) in the underlying model. We normalized the raw values to percentages as shown in Figure 3 to enable direct comparison of transmission risks across continents. In addition, the absolute Markov reward has been provided in Supplementary Table 4 in the original version.

4. Lines 263-264 "A Bayesian phylogeographic GLM was used to reconstruct the spatiotemporal dynamics of H7 viruses and assess potential predictors of spatial diffusion", Please explain in detail the Bayesian phylogenetic GLM method. Can the GLM method reconstruct the spatiotemporal dynamics of H7? Or if the author's expression is incorrect and clear, the GLM method should be used to evaluate the impact of spatial diffusion.

Response 4:

Thank you for your constructive and insightful comment. We acknowledge that the original phrasing could be misinterpreted and have revised the text to precisely reflect the method. The GLM is incorporated into the discrete phylogenetic framework to investigate the potential predictors' contribution to the dispersal between locations. Actually, the GLM does not directly reconstruct spatiotemporal dynamics but quantifies how predictors modulate diffusion rates inferred from phylogeny. In light of these concerns, we have revised the expression to maintain methodological rigor and correct.

Change in the text (the Results section):

[Page 11 , Lines 235-236]

A Bayesian phylogeographic GLM was used to assess potential predictors of spatial diffusion of H7 viruses.

5. Lines 265-266 The sentence "showing a strong negative association with transmission (coefficient = -1.015, 95% CI: -1.173 to -0.879)", Please verify whether it should be 95% CI or 95% highest posterior density (HPD), as the Line 270 shows 95% HPD.

Response 5:

We sincerely appreciate the reviewer's attention to methodological terminology. The reviewer's

comment is absolutely correct that the interval reported in Lines 265-266 should be specified as 95% Highest Posterior Density (HPD) rather than confidence interval (CI). This was an oversight in terminology during manuscript preparation. We are sorry that we did not detect this error when checking the article. Thank you again for your correction.

Change in the text (the Results section):

[Page 12, Lines 240-242]

Geographic distance was strongly negatively associated with viral spread (coefficient = -0.873, 95% HPD: -0.995 to -0.753).

6. Lines 291-292 What does the sentence "The number of triangles represents the order of the number of outbreaks" mean? Please reorganize the language.

Response 6:

We sincerely appreciate the reviewer's feedback regarding the ambiguity in the caption. The original phrasing did not adequately convey the intended meaning. We have reorganized the description to clarify the relationship between triangle quantity and outbreak frequency ranking.

Change in the text (the Results section):

[Page 46, Lines 930-932]

The number of triangles on each continent corresponds to its ascending rank in outbreak frequency during the same period (e.g., △=lowest, △△=intermediate, △△△=highest).

7. Lines 293-294 The arrows of different colors represent the transmission routes of different subtypes. Do they spread in a straight line along the direction of the arrows, and how do they spread?

Response 7:

We regret the lack of sufficient detail interpretation of directional arrows. We acknowledge that the original caption did not sufficiently clarify the spatial representation of transmission routes. The colored arrows do not represent literal geographic paths but instead indicate directionality of intercontinental spread according to the result of Markov jumps. We have amended the figure caption to avoid misunderstandings.

Change in the text (the Results section):

[Page 46, Lines 933-934]

These colored arrows do not represent literal geographic paths but instead indicate directionality of intercontinental spread according to the result of Markov jumps.

8. Line 346 There are occasional language errors throughout the entire text, such as "A large fraction of poultry products are traded" should be changed to "A large fraction of poultry products is traded". Please read and polish thoroughly.

Response 8:

We sincerely appreciate the reviewer's meticulous attention to linguistic precision throughout the manuscript. We acknowledge that occasional grammatical inaccuracies in "A large fraction of poultry products are traded" which may detract from the scientific rigor of our presentation. We have also carefully proofread the text to ensure consistency in tense, article usage, and phrasing throughout. Thank you again for this suggestion, which has significantly improved our paper.

Change in the text (the Results section):

[Page 15, Line 304]

A large fraction of poultry products is traded through live poultry markets in Asia.

9. Lines 421-424 The discussion of the biological mechanisms that negatively correlate with temperature in the existing text is somewhat vague. I suggest adding 1-2 sentences to clarify the significance of this result for the spread of avian influenza in the context of climate change.

Response 9:

Thank you for your insightful comment. We sincerely appreciate the reviewer's valuable suggestion to clarify the biological mechanisms underlying the temperature-transmission relationship. We have added two key sentences in the Discussion section to explicitly articulate the biophysical mechanism of temperature-dependent viral degradation and climate change implications for avian influenza risk management.

Change in the text (the Discussion section):

[Page 19, Lines 405-408]

Influenza viruses degrade more quickly in warm or dry conditions[53], so high temperature may reduce viral persistence in the environment, which explains the negative correlation between temperature and transmission risk.

[Page 20, Lines 418-421]

Integrating real-time environmental variables such as temperature monitoring into early warning systems is important, particularly in regions with high poultry-human contact. Timely interventions such as market closures or poultry vaccination campaigns are crucial during the periods of climatically favorable viral transmission.

[53] Prosser, D. J., Teitelbaum, C. S., Yin, S., Hill, N. J. & Xiao, X. Climate change impacts on bird migration and highly pathogenic avian influenza. Nature microbiology 8, 2223-2225 (2023).

10. Lines 415-444 This paragraph of discussion has too many words, which affects readers' reading and understanding. It is recommended to have a segmented and targeted discussion.

Response 10:

We thank the reviewer for this constructive suggestion. As recommended, we have made it more concise and restructured this part into two thematic paragraphs. One paragraph discussed in detail the significant factors that affect transmission, and the other paragraph discussed what these effects mean for our efforts to control avian influenza.

Change in the text (the Discussion section):

[Pages 19-20, Lines 397-425]

To the best of our knowledge, this study represents the first application of the GLM approach to the H7 AIVs, offering a novel perspective on its transmission dynamics. Geographic distance is identified as a consistently significant factor influencing migration rates, demonstrating that intercontinental transmission intensity decreases with increasing distance, consistent with spatial diffusion constraints. This discovery is consistent with previous research on H9N2 subtype[50]. Chicken stock origin consistently made a positive contribution to the spread of virus, while temperature origin was considered as a supportively negative predictor. Environmental factors that

may influence transmission include the stocking density, temperature, and airflow[51]. It was estimated 2-22 ° C a risky window for human H7N9 infection in previous study[52]. Influenza viruses degrade more quickly in warm or dry conditions[53], so high temperature may reduce viral persistence in the environment, which explains the negative correlation between temperature and transmission risk. Besides, many supplied birds arrive already exposed to AIVs, resulting in many broiler chickens entering the market as infected[54], probably causing the virus to remain in the chicken. And sample size was also an important factor. GLMs that include and do not include sample size predictors were both conducted to examine whether the sample size may mask the effects of other variables. When including sample size in the model, the economically developed regions may have stronger prevention and control capabilities including health facilities and quarantine measures and significantly reduce the risk of transmission[55].

These findings suggest that we can focus on the following aspects for the prevention and control of H7 viruses: First, enhance surveillance in regions with small sample sizes, particularly in areas with dense poultry farming and along migratory bird routes, to enable early detection and containment of virus transmission. Integrating real-time environmental variables such as temperature monitoring into early warning systems is important, particularly in regions with high poultry-human contact. Timely interventions such as market closures or poultry vaccination campaigns are crucial during the periods of climatically favorable viral transmission. Second, optimize resource allocation by prioritizing the prevention and control efforts in the virus origin areas and their surrounding regions to reduce the risk of local spread. Finally, improve monitoring capabilities in low-income regions through international cooperation to minimize the impact of sampling bias on the assessment of virus transmission.

11. Lines 458-475 The conclusion is a bit lengthy, please condense it.

Response 11:

Thank you for your helpful suggestion.We have thoroughly revised the Conclusion section to eliminate redundant statements and focus solely on the core findings and their implications. The revised version is now more focused and highlights the main takeaways of our study more clearly.

Change in the text (the Discussion section):

[Page 21, Lines 441-452]

In conclusion, this study provides a comprehensive global analysis of the spread, evolution, and host transmission dynamics of H7 avian influenza viruses. Through phylogenetic and spatial modeling, we reveal distinct lineage diversification patterns and identify key ecological and human-driven factors influencing cross-species transmission. Our findings highlight how geographic distance, temperature, GDP, and poultry density collectively shape viral dispersal across regions. By integrating genetic, spatial, and host data, this study advances understanding of H7 virus evolution and pandemic potential. To mitigate the risk of cross-species transmission and global spread of H7 viruses, this study highlights the need for One Health-oriented strategies grounded in multisectoral collaboration and interdisciplinary integration, aimed at strengthening genomic surveillance and transboundary data sharing, with particular attention to Anseriformes on the migration routes and high-risk regions such as Asia and Africa that are severely affected by avian influenza.

12. Line 471-472 The One Health-oriented strategy needs more depth. Rather than providing actionable recommendations, it just mentions high-risk areas and targeted surveillance in a general way. While the results find important phenomenon such as there have been a relatively high number of H7 virus outbreaks in Africa in recent years, the author does not offer discussion how this finding should enlighten specific policies. For example, the authors may propose directing resources to regions severely affected by avian influenza.

Response 12:

Thank you for your helpful comment. We have revised the discussion to provide concrete policy guidance based on our findings, particularly regarding recent H7 outbreaks in Africa. The results in Figure 1 showed that there have been more outbreaks of avian influenza in Africa in recent years, and it is therefore recommended to pay particular attention to Anseriformes on the migration routes and high-risk regions such as Asia and Africa that are severely affected by avian influenza, and where more resources should be allocated on a priority basis.

Change in the text (the Discussion section):

[Page 21, Lines 447-452]

To mitigate the risk of cross-species transmission and global spread of H7 viruses, the study highlights the need for a One Health-oriented strategies grounded in multisectoral collaboration and interdisciplinary integration, aimed at strengthening genomic surveillance and transboundary data sharing, with particular attention to Anseriformes on the migration routes and high-risk regions such as Asia and Africa that are severely affected by avian influenza.

13. Line 473 The conclusion advocates for targeted monitoring in high-risk areas, but lacks description of specific regions. The research findings should be used to define 'high-risk' regions/hosts (e.g. prioritizing the attention of Anseriformes on the migration routes of East Asia or live poultry trading centers in Asia).

Response 13:

Thank you for this thoughtful suggestion. We agree that specifying high-risk regions would make our recommendations more actionable. We have revised the conclusion to define high-risk areas based on our findings, explicitly highlighting regions such as Anseriformes on the migration routes and high-risk regions such as Asia and Africa. This change strengthens the practical implication of our work.

Change in the text (the Discussion section):

[Page 21, Lines 447-452]

To mitigate the risk of cross-species transmission and global spread of H7 viruses, the study highlights the need for a One Health-oriented strategies grounded in multisectoral collaboration and interdisciplinary integration, aimed at strengthening genomic surveillance and transboundary data sharing, with particular attention to Anseriformes on the migration routes and high-risk regions such as Asia and Africa that are severely affected by avian influenza.

14. Line 575 There is an extra space after the bracket, Please carefully check the punctuation throughout the entire manuscript.

Response 14:

We sincerely thank the reviewer for attention to the format issue. We have corrected the extra spaces you mentioned in the revised manuscript. Furthermore, following your suggestion, we have

conducted a thorough check of the punctuation throughout the entire manuscript to ensure correctness. We appreciate your help in improving the quality of our manuscript.

Change in the text (the Methods section):

[Page 26, Lines 565-566]

The mean branch dispersal velocity vbranch (vbranch) was also estimated using the spatio-temporal information extracted from posterior trees using the spreadStatistics function as equation (3).

15. Given complexity of the methods in this study, I wonder whether a pipeline added in Methods could help clarify this.

Response 15:

Thank you for this excellent suggestion. We agree that a pipeline will greatly help in clarifying the complex workflow of our study and have added a detailed workflow diagram as follows.

Change in the text (the Methods section):

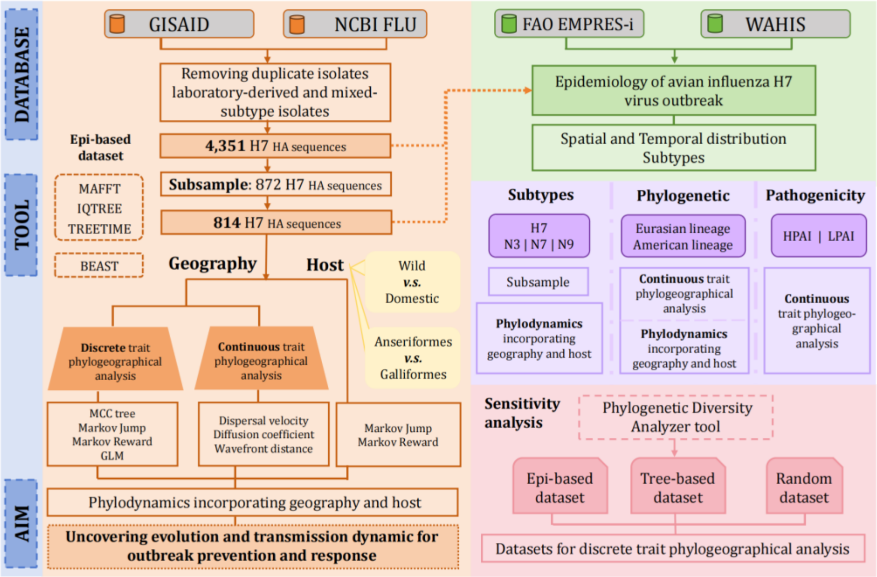

Fig. 11. Overview of the analytic pipeline for dataset collection, analytical method application and model development evaluation.

Reviewer #2:

This paper presents an analysis of the spread of avian influenza H7 viruses using phylodynamic and phylogeographic techniques on publicly accessible sequence data from 2000 to 2024 (present). The analysis is split into subtypes, the transmission patterns between host species and spatial dispersion patterns are analysed.

The methods used for the analyses are generally good and well described, and mirrors what has already been done for H5 (highly pathogenic). However, I have a few detailed comments which will hopefully improve the study design, analysis and interpretation of results.

Response:

We greatly appreciate your time and effort in reviewing our manuscript, and we are pleased that you found the methods to be generally good and well-described. Thank you for your positive and constructive feedback on our manuscript. We also thank you for drawing the parallel to the existing work on H5 viruses; we aimed to provide a similarly rigorous analysis for the H7 virus, which we believe is equally critical for understanding avian influenza dynamics.

We also appreciate your detailed comments and these provided us with excellent guidance to improve the study design, analysis, and interpretation of our results. We have addressed each of your points in detail below. We believe that following your suggestions has strengthened the manuscript.

1. Please check the wording of the first sentence, references 1 and 2 relate to H5 (& H13 / H16) viruses, only reference 3 mentions H7. Suggest remove 'particularly those of the H7 subtype'.

Response 1:

Thank you for your instructive comment. We have removed the phrase 'particularly those of the H7 subtype' in the first sentence, since References 1-2 do not specifically involve H7 viruses. The revised text now accurately reflects the scope of the cited literature.

Change in the text (the Introduction section):

[Page 2, Lines 34-35]

Avian influenza viruses (AIVs) pose a persistent threat to both animals and human health due to their potential to cause zoonotic infections and evolve into highly pathogenic forms.

2. I think you need to mention low and high pathogenicity types for H5 and H7 in the introduction;

this is important because most of the recent H5 spread studies have been on high pathogenicity (and specific clades of H5) types, whereas you are considering both low and high pathogenicity types of H7 - you don't distinguish between them, and do not explain why you chose to consider them together.

Response 2:

We sincerely thank the reviewer for this insightful comment. It is important to clarify the distinction between low pathogenicity (LPAI) and high pathogenicity (HPAI) avian influenza viruses, particularly for H5 and H7 subtypes, which is essential for framing the context and rationale of our study. As you suggested, we have also distinguished between transmission modes of high pathogenicity and low pathogenicity of H7 and subtypes, and have added this description in Introduction section (as follows) and analysis in the Results and Discussion section (Please refer to **Response to comment 10**).

Change in the text (the Introduction section):

[Page 2, Lines 37-47]

Historically, outbreaks caused by high-pathogenic avian influenza (HPAI) viruses have mostly arisen from virulence shifts by low-pathogenic avian influenza (LPAI) viruses. Notably, HPAI H5 virus has demonstrated unprecedented pandemic potential by becoming endemic in bird reservoirs and causing widespread infections across 84 countries in Asia, Africa, Europe, and North America[6]. The mechanisms by which LPAI viruses of the H5 and H7 subtypes evolve into HPAI viruses involve mutations, insertions, and recombination events occurring at the proteolytic cleavage site of the hemagglutinin (HA) protein, playing a critical role in mediating the virus's attachment to host cell receptors, thereby initiating the viral infection cycle[6]. While much attention has focused on the H5 subtype, the transmission dynamic and ecological drivers of H7 viruses remain comparatively underexplored.

[Page 3, Lines 60-62]

However, the roles of different host groups in the global dissemination and cross-species transmission of H7 subtypes remain poorly understood, and it is still unclear how fast the LPAI and HPAI H7 viruses spread among diverse host populations.

[Page 4, Lines 70-72]

quantifies the relative contributions of key avian host groups to viral spread, specifically

investigating the differences in dispersal velocity of LPAI and HPAI H7 viruses among these hosts.

[6] Lee, D. H., Criado, M. F. & Swayne, D. E. Pathobiological Origins and Evolutionary History of Highly Pathogenic Avian Influenza Viruses. Cold Spring Harbor perspectives in medicine 11, a038679 (2021).

3. Besides the data in figure 1 (cases, sequences, hosts), since the methods section of this paper is at the back, please include a very brief description of what the dataset for phylogenetic analysis is in the main text (noted that the details are in the supplementary). Something along the lines of 'We complied four data sets of segment 4 (coding for the hemagglutinin surface protein) sequences from 2000-2024, from A: a sample of all hosts and H7 subtypes; B: H7N3 only; etc.. after subsampling these ranged in size from X-Y, please see methods [etc] for details'. This needs to come before, or at the start of the 'Spatial diffusion of H7 virus section'.

Response 3:

Thank you for your helpful suggestion. As recommended, we have now added a very brief description of what the dataset for phylogenetic analysis at the start of the 'Spatial diffusion of H7 virus' section to improve the clarity and flow of the manuscript.

Change in the text (the Results section):

[Page 6, Lines 106-109]

For phylogenetic and phylogeographic analysis, we complied four data sets of HA gene (coding for the hemagglutinin surface protein) sequences from 2000-2024, from A: a sample of all H7 subtypes and hosts; B: H7N3 only; C: H7N7 only; D: H7N9 only. The 'H7' dataset contains all the subtypes. After subsampling these ranged in size from 430-814, please see Methods section for details.

4. Page 5 - suggest "Supplementary figure 3 shows a relatively even spatiotemporal distribution of sequences, which could support".

Response 4:

We thank you for the constructive suggestion to clarify the interpretation of Supplementary figure 3. We have revised the sentence to reflect that the even spatiotemporal distribution could support

unbiased analysis.

Change in the text (the Results section):

[Page 5, Lines 96-97]

Supplementary Fig.3 shows a relatively even spatiotemporal distribution of sequences, which could support unbiased and comprehensive analysis of virus dynamics.

5. Page 5 - The use of ‘widespread’ in this sentence is misleading - suggest removing it [and/or something like this - “Multiple local spill over events of H7N9 from poultry to humans were observed between 2013 and 2017, and the inferred genetic diversity (Ne) showed a declining trend. This decrease may be attributed to..”]

Response 5:

We thank the reviewer for this critical correction. We agree that ‘widespread’ was an inappropriate expression to describe the transmission dynamics of H7N9, which were characterized by repeated spillover events rather than sustained human-to-human transmission. We have removed 'widespread' and have replaced the entire sentence with the reviewer's suggested text.

Change in the text (the Results section):

[Page 5, Lines 100-103]

Multiple local spill over events of H7N9 from poultry to humans were observed between 2013 and 2017, and the inferred genetic diversity (Ne) showed a declining trend. This decrease may be attributed to a combination of viral lineage bottlenecks, effective control measures, and sampling biases.

6. Page 7 - Spatial diffusion of H7 virus

It is not clear from the text as it currently is what the four datasets are; specifically you need to put in the main text that the ‘H7’ dataset contains all the subtypes. Aside question - why did you not exclude the sequences with only H7 subtype listed and no NA type ?

Response 6:

Thank you for these insightful comments. We have now revised the text and added a brief

description of what the four datasets at the start of the 'Spatial diffusion of H7 virus'. We believe this clarification eliminates potential ambiguity.

We chose to retain the sequences with only the H7 subtype for two primary reasons: **First**, excluding sequences without NA subtype could reduce the representativeness of the entire H7, especially if these excluded sequences have data for certain time periods, geographic regions, or host species and may misrepresent the true genetic diversity and temporal-spatial distribution of H7 virus. **Second**, to maximize data utilization for our analysis, since the primary focus of our phylogenetic and phylogeographic analysis is on the whole H7 subtype, and sequences with a confirmed H7 HA are entirely valid and informative for addressing core research questions.

Change in the text (the Results section):

[Page 6, Lines 106-109]

For phylogenetic and phylogeographic analysis, we complied four data sets of HA gene (coding for the hemagglutinin surface protein) sequences from 2000-2024, from A: a sample of all H7 subtypes and hosts; B: H7N3 only; C: H7N7 only; D: H7N9 only. The 'H7' dataset contains all the subtypes. After subsampling these ranged in size from 430-814, please see Methods section for details.

7. Figure 2 - maybe add a bar for subtype and continent to all sub-figures (so they all have 3 bars)? Also, can you indicate which are highly pathogenic? (Another bar ?).

Response 7:

Thank you for this insightful comment. In response, we have ensured that the H7 MCC tree panel include information on virus subtype, continent, host, and pathogenicity, while subtype-specific panels displayed 3 bars (continent, host and pathogenicity). The continental information was also visualized through terminal branch coloring to facilitate the delineation of lineages. A consolidated legend improves readability without redundancy.

Change in the figure (the Results section):

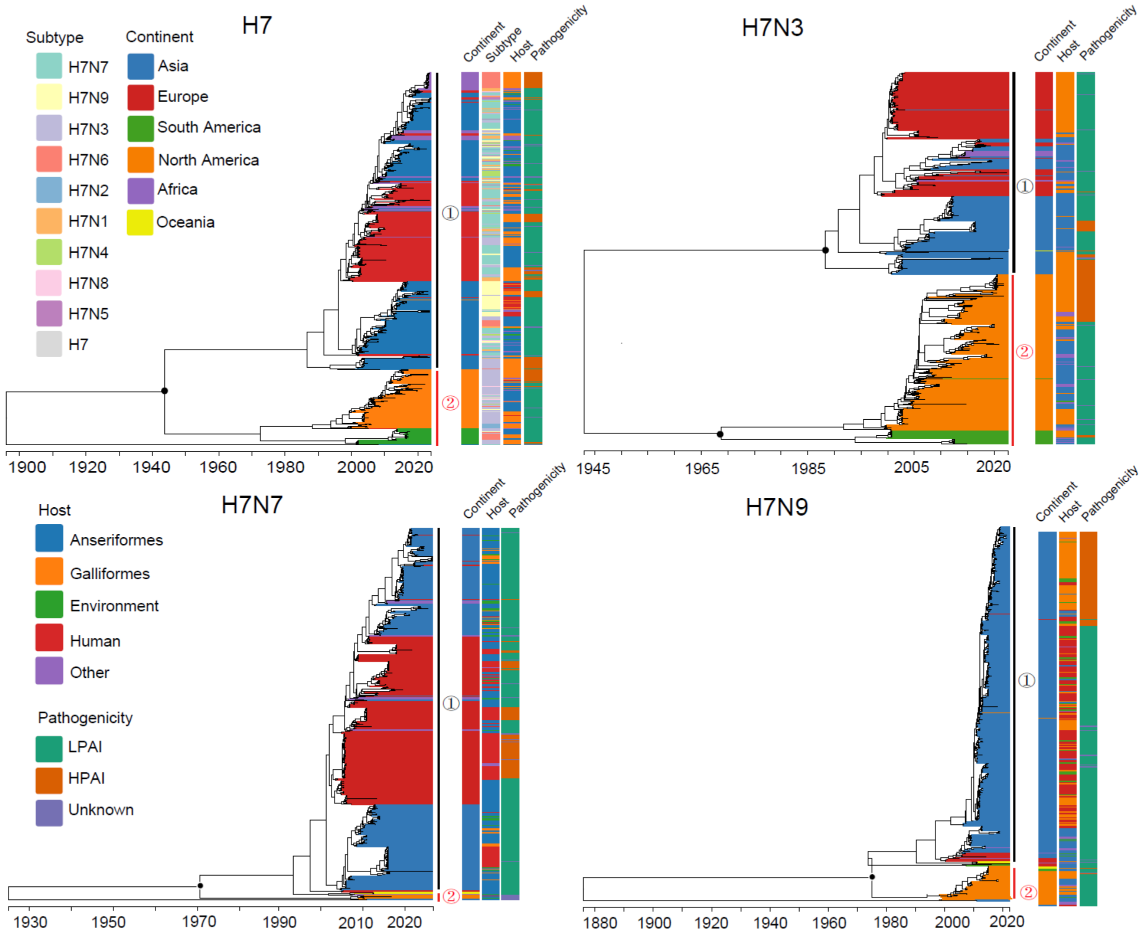

Fig. 2. Time-scaled maximum clade credibility (MCC) tree of H7 HA genes sequences.

The evolutionary relationships of the H7 HA gene segment were reconstructed using full-length sequences from viruses collected across different geographic regions. The geographic information was displayed in the end branch. The sequences in HA lineage □ were mainly from Asia and Europe, so we named □ as Eurasian lineage. The sequences in HA lineage □ were from America, so we named □ as American lineage.

8. Page 9 / Figure 3 - considering that the H7N3, H7N7, H7N9 subtypes do not form monophyletic clades (of only those subtypes) in the H7 (all subtypes) tree - is it valid to calculate Markov rewards in those subtypes ? (I am suggesting that it is not).

Response 8:

We sincerely thank the reviewer for this critical comment. We agree that it is not valid to calculate Markov rewards for H7N3, H7N7, H7N9 subtypes. Therefore, we do not calculate Markov rewards

for subtypes and revised our manuscript. We have updated Figure 3.

Change in the text (the Results section):

[Page 7, Lines 141-143]

Similarly, H7N3, H7N7 and H7N9 exhibited extended presence in Asia, with 28.38%, 24.88% and 19.40% Markov jumps from Asia to Europe, respectively, and these Markov jumps had high bayes factors (Fig. 3, Supplementary Table 3-4).

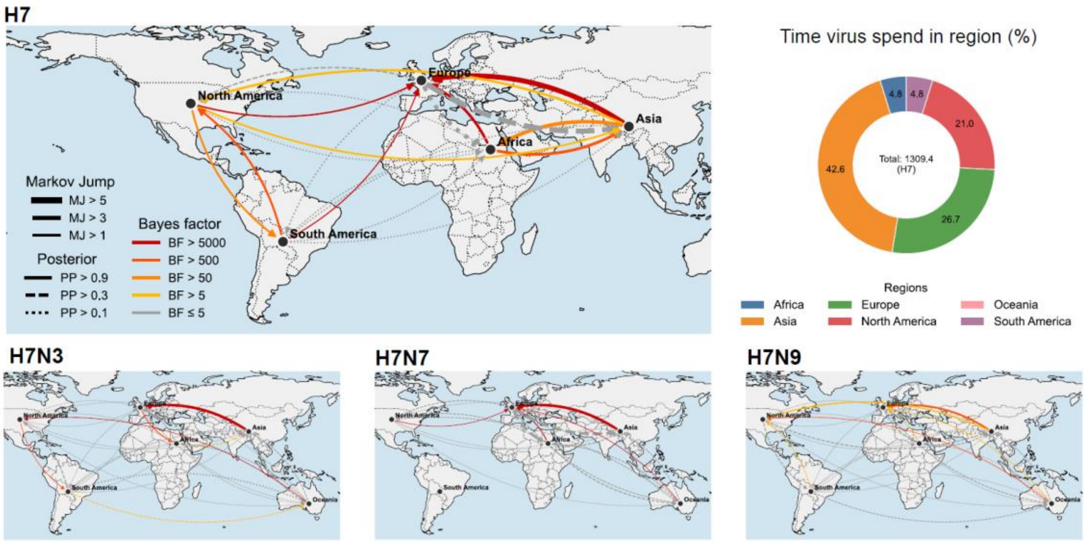

Fig. 3. Contrasting geographic transmission patterns of H7 subtypes.

Each panel illustrated spatial diffusion patterns inferred from discrete phylogeographic analysis. From left to right, the plots show regional Markov jump counts (transmission events), Markov rewards (residence times), and Bayes factors indicating the statistical support for transmission routes.

9. Pages 10-13 - for the more detailed continuous trait phylogeography; especially per subtype - suggest that you analyse individual monophyletic clades (initial outbreaks + spread) separately, and not mix geographically and genetically unconnected outbreaks. Just because the HA H7's are paired with the same subtype numbered NA does not mean that the viruses are particularly closely related. The same idea of not mixing geographically and genetically unconnected outbreaks also applies for the Host transmission analysis on pages 12 and 13.

Response 9:

Thank you for this insightful comment. According to your comment, we have further analysed individual monophyletic clades (initial outbreaks + spread) separately. **Firstly**, from the new Figure 2, we identified the major monophyletic lineages within each subtype based on clear geographical clustering and separately extracted the following lineage: ①Eurasia lineage; ②American lineage. **Secondly**, we have re-performed all continuous trait phylogeography analysis on individual monophyletic lineages to reconstruct the spatial transmission dynamics of H7 virus and subtypes. **Besides**, we have also re-ran the host transmission analysis for each lineage of subtyes to estimate host jumping, dispersal velocity, wavefront distance, and diffusion coefficient.

The results of these new analyses were presented in Figs 4-8; Supplementary Figures 7-17. The new results provide a much clearer and more precise picture of transmission patterns of each viral lineage. We are grateful to the reviewer for significantly improving the rigor of our study.

Change in the text (the Results section):

[Pages 7-8, Lines 144-169]

Continuous phylogeographic reconstruction provides a detailed depiction of the temporal and spatial transmission of H7 viruses across the transmission network (Fig. 4, Supplementary Fig.7). The continuous phylogeography analysis suggests that Eurasian lineage of H7 virus has two primary transmission trajectories. One transmission route showed westward spread of the virus from East Asia into Southeast and West Asia, while another indicated eastward movement from Western Europe into West Asia. By around 2010, the virus converged in West Asia before spreading to South Africa. For the American lineage, transmission occurred primarily in the northern United States along the Canadian border, with subsequent southward spread into Mexico. Overall, H7 viruses exhibited sustained and widespread transmission across multiple continents. The Eurasian lineages of H7N3 and H7N7 circulated actively within Eurasia, while the Eurasian lineage of H7N9 spread extensively in East Asia, particularly in China after 2015. The American lineage of the H7N3 virus has circulated widely across the United States, extending as far as Alaska, and over the past decade has spread southward into Mexico, where it remains actively transmitted. The American lineage of H7N9 exhibited a similar transmission pattern, whereas the H7N7 lineage showed comparatively limited circulation.

Furthermore, the spatial expansion of the Eurasian lineage and American lineage of H7 viruses in

continuous space showed a median weighted branch dispersal velocity of 661.23 km/year (95% HPD: 400.12 - 733.47) and 354.18 km/year (260.13 - 444.50), respectively (Fig. 5). Similarly, the median dispersal velocities for the Eurasian lineage of H7N3, H7N7, and H7N9 subtypes were estimated at 585.37 km/year (514.10 - 648.87), 645.28 km/year (512.61 - 747.96), and 562.48 km/year (380.76 - 689.73), respectively (Supplementary Fig.8-10). Wavefront distance was defined as the mean great-circle distance from the epidemic origin to the most recently circulating lineages. As of the latest estimate, the Eurasian lineage of H7 viruses exhibited a wavefront distance of 10,986.55 km (7,625.84 - 14,640.81), compared with 7,311.98 km (5,380.59- 11,971.32) for the American lineage (Fig. 5). The diffusion coefficient was consistently higher for the Eurasian lineage than for the American lineage.

[Page 9, Lines 184-207]

Host Transmission Patterns

The Eurasian lineage of the H7N9 virus exhibited a similar pattern (Supplementary Tables 5-6). We also analyzed the transmission dynamics of hosts, classified as either wild or domestic, which included samples from Galliformes and Anseriformes. H7 viruses exhibited more frequent host transmissions between wild and domestic birds, with a slightly longer duration in wild birds than in domestic poultry (Supplementary Fig.14; Supplementary Table 7-8). In H7N9, particularly the Eurasian lineage, transmission from humans to domestic birds was more common than between wild and domestic birds. Compared with other subtypes, humans also displayed higher Markov rewards, highlighting their prominent role in virus transmission.

To evaluate host contributions to spatial transmission, we conducted a combined analysis integrating discrete host states with continuous geographic data. The Eurasian lineage of H7 virus spread more rapidly among Anseriformes (586.51 km/year, 518.90 - 682.14) and wild birds (654.60 km/year, 595.14 - 751.59) than among other hosts, whereas Galliformes exhibited the slowest dispersal velocity (294.44 km/year, 253.42 - 343.79) (Fig. 7). This pattern was consistent across subtypes: Anseriformes displayed significantly higher dispersal velocity than Galliformes in H7N3 (Mann-Whitney U test, $P < 0.01$) , H7N7 ($P < 0.01$), and H7N9 ($P < 0.01$) (Supplementary Fig. 11-13; Supplementary Table 9). Wild birds generally transmitted viruses faster than domestic poultry in H7, H7N3, and H7N9 ($P < 0.01$), whereas in the Eurasian lineage of H7N7, domestic birds

contributed more to the rapid transmission than wild birds (P < 0.01) (Supplementary Figs. 11-13, Supplementary Table 9). Results based on mean branch dispersal velocities were consistent with weighted estimates (Supplementary Fig.15).

Wavefront distance and diffusion coefficient were estimated using the continuous diffusion model (Fig. 8). Wavefront distance varied by host type: in the latest estimates, Anseriformes exhibited a wavefront distance of 12,096.978 km (7,901.05 - 12,096.978), exceeding that of Galliformes (9,609.90 km, 8,097.98 - 11,927.54) for H7 virus.

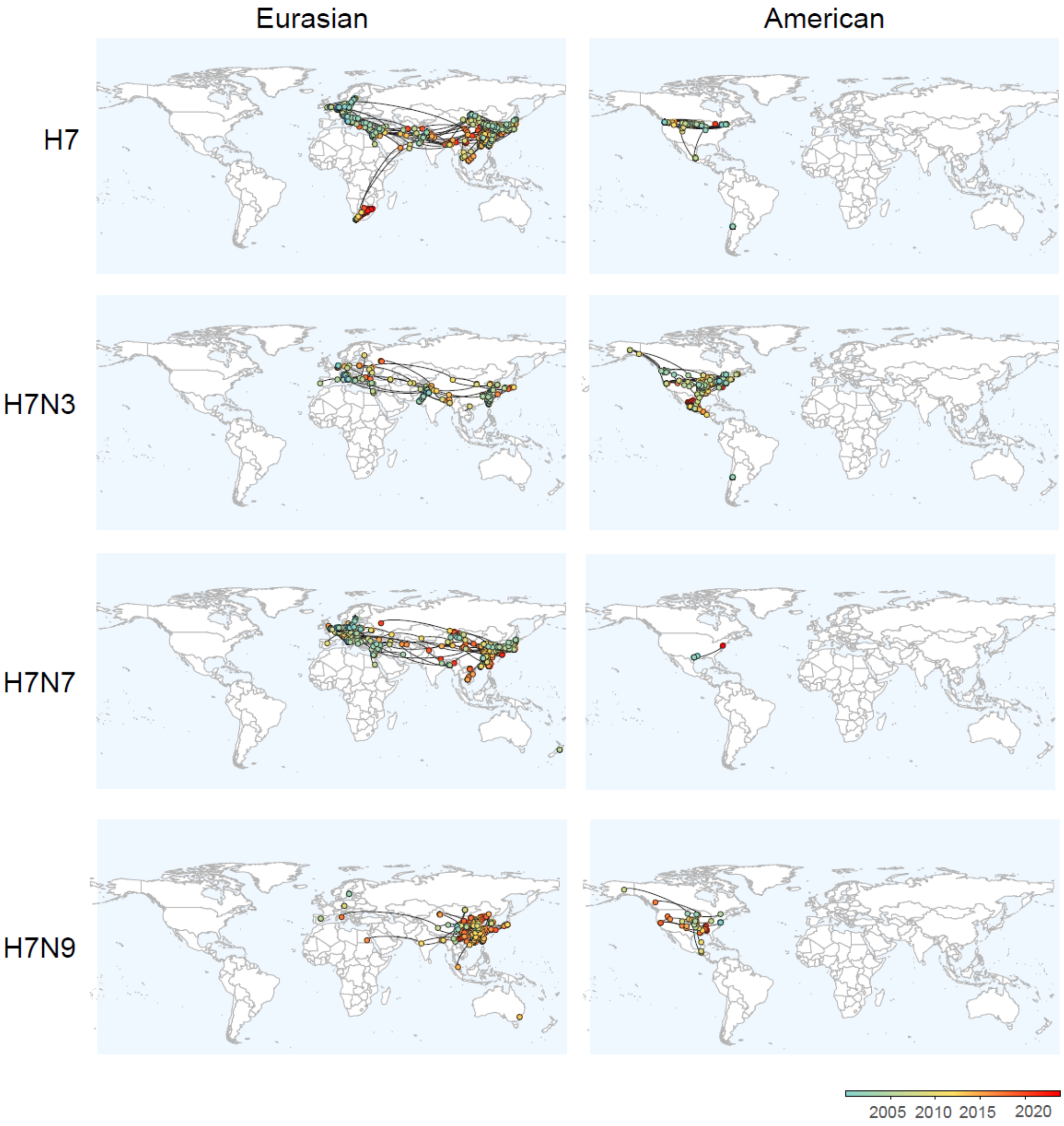

Fig. 4. Continuous phylogeographic reconstruction of H7 and its subtypes.

Spread of H7 virus and its subtypes from 2000 to 2024. Circles represent nodes in the maximum clade credibility phylogeny, colored by the inferred time of occurrence, with green indicating early time and red indicating late time.

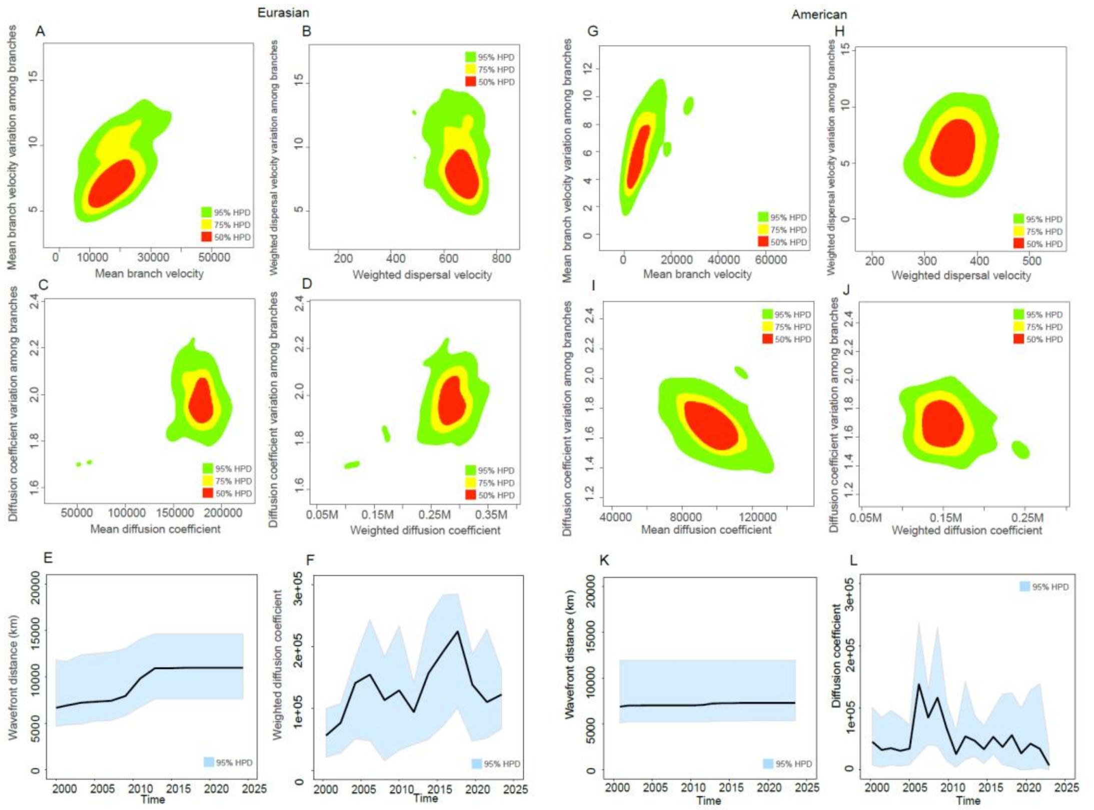

Fig. 5. Estimated dispersal statistics for H7.

The two columns on the left represent the Eurasian lineage, while the two columns on the right represent the American lineage. A and G, kernel density estimates of mean branch dispersal velocity parameters. B and H, kernel density estimates of weighted branch dispersal velocity parameters. C and I, kernel density estimates of original diffusion coefficient parameters. D and J, kernel density estimates of weighted diffusion coefficient parameters. E and K, furthest extent of epidemic wavefront (spatial distance from epidemic origin). F and L, Evolution of weighted diffusion coefficient.

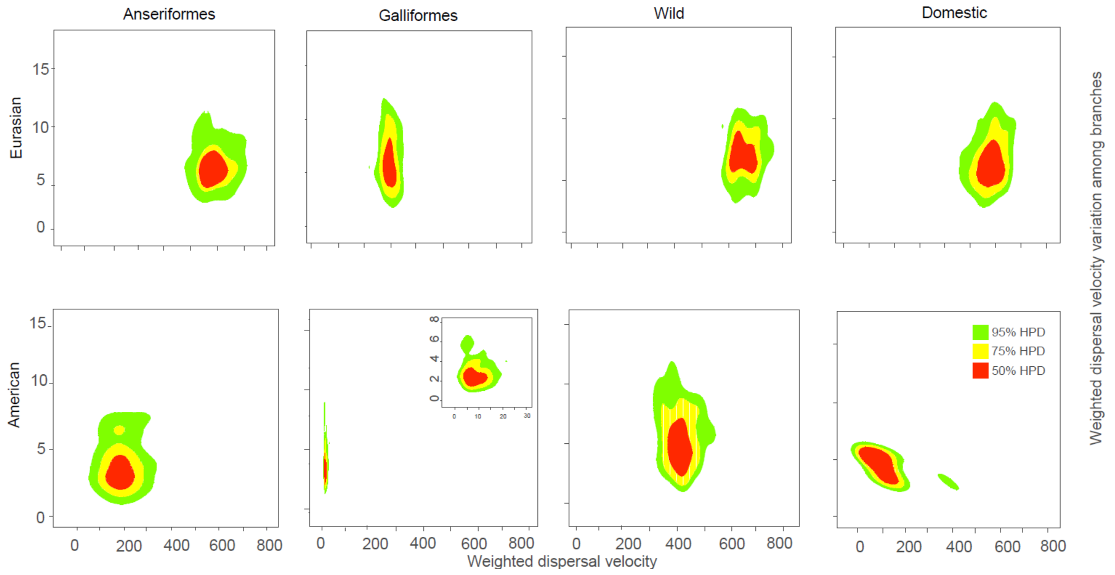

Fig. 7. Host-specific dispersal velocity estimates for H7.

The top row represents the Eurasian lineage, while the bottom row represents the American lineage. Each panel shows dispersal velocities stratified by host type. Shaded contours represent the 50%, 75%, and 95% highest posterior density (HPD) regions, estimated via kernel density methods, with darker shades indicating higher certainty.

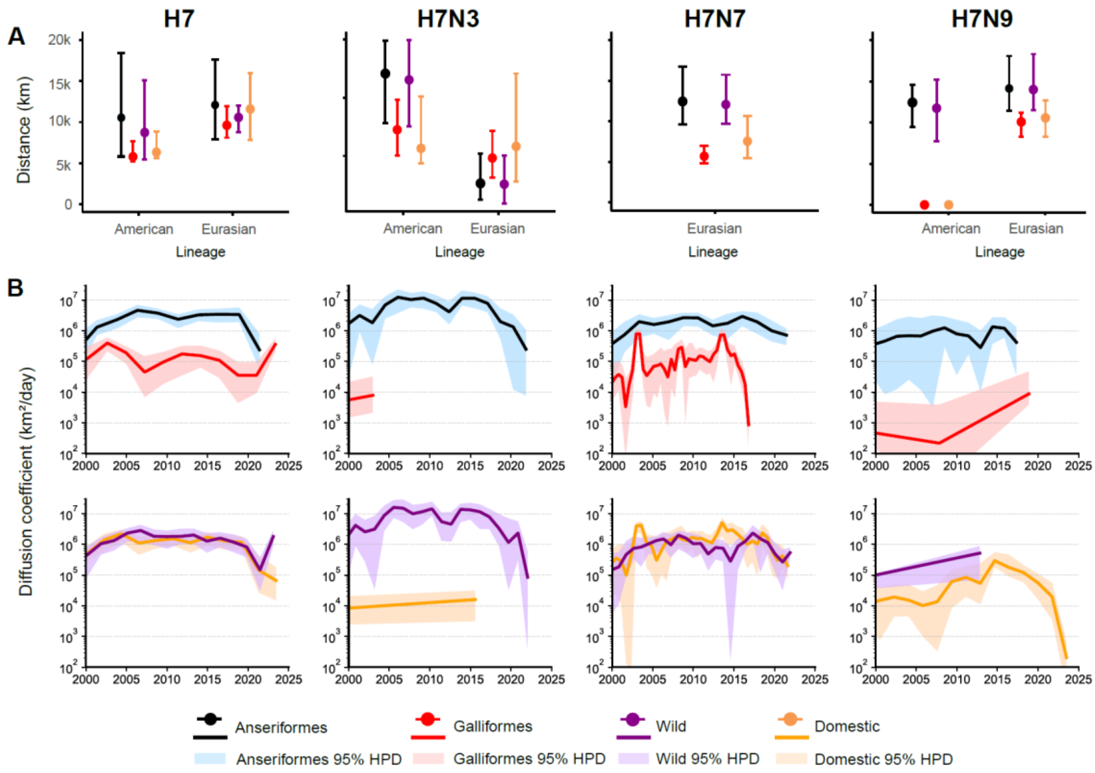

Fig. 8. Host-specific wavefront distance and diffusion coefficient estimates for H7 and its

subtypes.

Estimated wavefront distances (km) for H7 virus and subtypes. The wavefront distance represents the final extent of spatial spread; (B) Weighted diffusion coefficient (km ^{2}/day). Shaded areas represent 95% highest posterior density (HPD). Diffusion estimates for certain years are omitted due to irregular values likely resulting from limited sampling.

10. Page 13 - regarding observation of faster spread in one species than another - is this also geographically segregated ? Or are the dispersal velocities the same in different continents ? And what about dispersal of high vs low pathogenicity viruses ? Would you expect to see slow spread of high pathogenicity H7 amongst galliformes in a restricted geographic area ? (Suggesting that this should be detectable).

Response 10:

Thank you for your valuable comments. **First**, we conducted a supplementary comparative analysis across different geographical locations in relation to viral lineages. Our lineage-specific analyses revealed that the Eurasian lineage of H7 viruses showed faster spatial expansion than the American lineage, with higher median branch dispersal velocities, greater wavefront distances, and larger diffusion coefficients. Across subtypes, H7N3, H7N7, and H7N9 of the Eurasian lineage all exhibited substantial dispersal velocity, highlighting their rapid geographic spread. These results are now summarized in new Fig 5 and Supplementary Figures 8-10.

Second, we have further conducted supplementary comparative analysis for dispersal velocity based on HPAI and LPAI virus according to your suggestion. Our dispersal velocity analysis showed that HPAI H7 viruses generally spread more slowly than LPAI viruses among domestic hosts, most notably in the H7N3 subtype, whereas in Galliformes, HPAI viruses tended to disperse faster. This is detailed in new Supplementary Table 10. This pattern likely reflects differences in ecology, host management, and transmission dynamics between HPAI and LPAI viruses. LPAI viruses circulate mainly in wild and domestic waterfowl, which migrate long distances and facilitate wider geographic spread, leading to higher dispersal velocities. In contrast, HPAI outbreaks typically occur in confined poultry populations, where rapid detection, culling, and movement restrictions limit spatial dissemination. However, in Galliformes, HPAI viruses may appear to spread faster due

to frequent poultry trade, farm density, and human-mediated movement, amplifying short-range but rapid transmission events.

Change in the text (the Results section):

[Page 8, Lines 159-169]

Furthermore, the spatial expansion of the Eurasian lineage and American lineage of H7 viruses in continuous space showed a median weighted branch dispersal velocity of 661.23 km/year (95% HPD: 400.12 - 733.47) and 354.18 km/year (260.13 - 444.50), respectively (Fig. 5). Similarly, the median dispersal velocities for the Eurasian lineage of H7N3, H7N7, and H7N9 subtypes were estimated at 585.37 km/year (514.10 - 648.87), 645.28 km/year (512.61 - 747.96), and 562.48 km/year (380.76 - 689.73), respectively (Supplementary Fig.8-10). Wavefront distance was defined as the mean great-circle distance from the epidemic origin to the most recently circulating lineages. As of the latest estimate, the Eurasian lineage of H7 viruses exhibited a wavefront distance of 10,986.55 km (7,625.84 - 14,640.81), compared with 7,311.98 km (5,380.59- 11,971.32) for the American lineage (Fig. 5). The diffusion coefficient was consistently higher for the Eurasian lineage than for the American lineage.

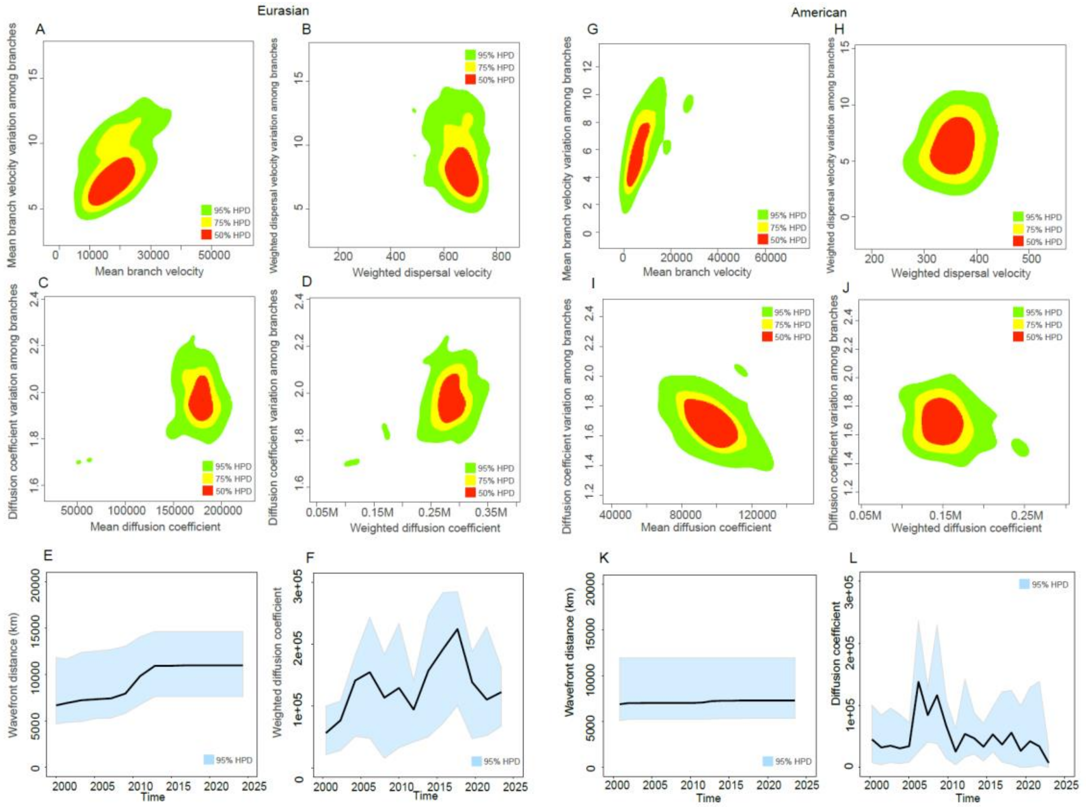

Fig. 5. Estimated dispersal statistics for H7.

The two columns on the left represent the Eurasian lineage, while the two columns on the right represent the American lineage. A and G, kernel density estimates of mean branch dispersal velocity parameters. B and H, kernel density estimates of weighted branch dispersal velocity parameters. C and I, kernel density estimates of original diffusion coefficient parameters. D and J, kernel density estimates of weighted diffusion coefficient parameters. E and K, furthest extent of epidemic wavefront (spatial distance from epidemic origin). F and L, Evolution of weighted diffusion coefficient.

[Page 11, Lines 218-227]

Host Transmission Patterns

Furthermore, dispersal velocity analysis revealed a clear pattern in the transmission of high and low pathogenic H7 viruses (Supplementary Table 10). Overall, H7 viruses exhibited higher dispersal velocities in Anseriformes than in Galliformes, and in wild birds compared with domestic birds. Among domestic hosts, HPAI viruses generally spread more slowly than LPAI viruses. This difference was particularly pronounced for the H7N3 subtype, where HPAI viruses transmitted at a median velocity of 37.30 km/year (21.77 - 55.83) in domestic birds, approximately one-seventh the rate of LPAI viruses (282.04 km/year, 218.76 - 376.17). In contrast, within Galliformes, HPAI viruses tended to disperse faster than LPAI viruses. However, due to limited data for Anseriformes and wild birds, direct comparisons between HPAI and LPAI viruses in these groups remain inconclusive.

Supplementary Table 10. The weighted branch dispersal velocity of high and low pathogenic H7 viruses and subtypes in different hosts (median value, 95% HPD).

Subtype	Anseriformes	Galliformes	Wild	Domestic
H7-HPAI	-	175.42 (129.97, 211.20)	274.63 (144.90, 1121.37)	196.67 (153.66, 256.48)
H7-LPAI	651.40 (581.88, 719.04)	156.14 (115.70, 199.84)	465.09 (122, 655.54)	540.60 (474.40, 610.32)
H7N3-HPAI	-	33.13 (18.49, 54.49)	-	37.30 (21.77, 55.83)
H7N3-LPAI	497.21 (430.18, 569.87)	77.06 (21.23, 126.09)	494.84 (413.20, 574.23)	282.04 (218.76, 376.17)

H7N7-HPAI	-	353.08 (279.38, 429.86)	-	364.20 (278.78, 430.15)
H7N7-LPAI	600.54 (508.59, 691.17)	144.85 (104.33, 212.12)	471.99 (395.99, 567.30)	774.76 (652.39, 860.32)
H7N9-HPAI	-	330.54 (252.64, 425.51)	-	290.48 (194.47, 396.10)
H7N9-LPAI	425.46 (313.98, 529.27)	247.20 (145.30, 349.14)	358.86 (263.01, 491.65)	335.67 (240.19, 461.93)

Note: HPAI, high-pathogenic avian influenza; LPAI, low-pathogenic avian influenza. "- " indicates insufficient data for calculation.

Change in the text (the Discussion section):

[Page 17, Lines 366-381]

The differential dispersal velocities between LPAI and LPAI provided critical implications for understanding H7 transmission. The markedly slower dispersal velocity of HPAI viruses in Galliformes, especially H7N3-HPAI, suggests that despite their severe impact, their geographic transmission may be limited within poultry populations due to high host mortality. Conversely, the rapid transmission of LPAI viruses in Anseriformes underscored their role as a silent reservoir for long-range dissemination, consistent with traditional conclusion[9]. The notable exception of H7N7-HPAI and H7N9-HPAI, which transmitted rapidly, highlighted that subtype-specific virological properties and probable local epidemiological factors can sometimes override the general trend of pathogenicity-defined transmission, warranting further investigation. One possible reason is that the emergence of an HPAI virus triggers an immediate and intensive surveillance response[45], heightening surveillance likely leads to much quicker detection and reporting of more cases. While the virus may not actually be moving faster, the interval between infection and detection is dramatically shortened, creating a surveillance artifact that inflates the estimated velocity in our phylogeographic models. Besides, HPAI H7N7 and H7N9 outbreaks may have occurred in regions with extremely high host density and could transmit could spread rapidly between susceptible farms before control measures took full effect.

[9] Olsen, B. et al. Global patterns of influenza a virus in wild birds. Science ***312****, 384 –388 (2006).*

[45] Lee, D. H., Criado, M. F. & Swayne, D. E. Pathobiological Origins and Evolutionary History of Highly Pathogenic Avian Influenza Viruses. Cold Spring Harbor perspectives in medicine ***11****,*

a038679 (2021).

11. Page 16 - H7N9 Human sequences; I think the way you have analysed and described these is very misleading. There is was not widespread human to human transmission of H7N9, the majority of the human cases were from poultry to human spill over events. It is only biased sampling of human cases, and under sampling of the transmitting birds which makes the trees look like there is persistence in humans. Suggest you remove this paragraph and concentrate on the avian species.

Response 11:

Thank you for this thoughtful suggestion. We fully agree with the reviewer's viewpoint. You are right to point out that there is no evidence for sustained human-to-human transmission of H7N9. We have removed this paragraph and concentrate on the avian species. Furthermore, we have reviewed the entire manuscript to ensure that no similar misinterpretations remain.

12. Page 20 - "In addition the long term presence of H7N9 in Asia may be related to its ability to adapt to poultry hosts.." - do the recent H7N9 sequences have the characteristic mutations for alpha 2,6 rather than alpha 2,3 ? (Reference 18 is about 1918 virus, not H7N9 mutations, or do you mean reference 19 - but that is about North America).

Response 12:

Thank you for your careful comment. Previous studies have suggested that the acquisition of mutations (e.g., Q226L, T160A, and G186V in HA) can shift receptor-binding preference from avian-type α 2,3-linked to human-type α 2,6-linked sialic acid receptors, indicating that the long-term persistence of the H7N9 subtype in Asia may be linked to its adaptive evolutionary changes. However, indeed, there was an error in the reference citation in our manuscript. We have removed the incorrect citation to Ref. 18 and have now cited the appropriate literature that specifically documents the evolutionary changes in receptor-binding preferences of H7N9 viruses.

Change in the text (the Discussion section):

[Page 14, Lines 283-286]

In addition, the long-term presence of H7N9 subtype in Asia may be attributed to its adaptive evolution. A key mechanism is the acquisition of mutations (e.g., Q226L, T160A, and G186V in HA) that shift receptor binding preference towards human-type α 2,6-sialic acid receptors from the avian-type α 2,3-sialic acid receptors[19, 20].

[19] Gao, R. et al. Human infection with a novel avian-origin influenza A (H7N9) virus. N Engl J Med 368, 1888–1897 (2013).

[20] Xiong, X. et al. Receptor binding by an H7N9 influenza virus from humans. Nature 499, 496-499 (2013).

13. Page 22 - why would 'long term adaption within the host may enhance the potential for cross species transmission' ? If the virus is well adapted to a host species, why does would that mean it could easily adapt to a different species (surely it would be less compatible rather than more). Also, as above - what are those mutations which allow partial binding to alpha 2,6 ?

Response 13:

Thank you for your thoughtful and constructive comment. The relationship between long-term host adaptation and cross-species transmission potential is nuanced. Two complementary mechanisms may explain why extended adaptation within a host species can enhance the potential for cross species transmission: (i) Sustained enzootic circulation leads to large viral population sizes, increasing the likelihood of generating rare mutations that confer fitness advantages in a new host[39]. (ii) Certain traits selected within the original host (e.g., high polymerase efficiency or replication robustness) may coincidentally "pre-adapt" the virus to another host. Such pre-adaptation effectively lowers the genetic barrier for host switching by reducing the number or specificity of mutations required. For example, avian-derived H5N1 or H7N9 viruses infecting humans may only need a single key substitution (PB2-E627K) to enable efficient interaction with the mammalian ANP32A protein and support replication in human cells[40].

Second, the key mutations in the hemagglutinin (HA) protein that have been experimentally shown to confer partial binding to human-type α 2,6-linked sialic acid receptors include G186V (broadens the receptor-binding site, particularly critical in H7N9)[20], Q226L (switches receptor preference from

avian α 2,3- to human α 2,6-linked sialic acids), and T160A (removes a glycosylation site, increasing accessibility of the receptor-binding site)[19]. We have now explicitly mentioned these mutations in the revised manuscript.

Change in the text (the Discussion section):

[Page 16, Lines 336-340]

And long term adaption within the host may enhance the potential for cross species transmission, possibly by increasing viral diversity, which favors rare advantageous mutations, and by selecting traits that pre-adapt the virus to new hosts, lowering the genetic barrier for cross-species transmission[39-40].

[Page 14, Lines 284-286]

A key mechanism is the acquisition of mutations (e.g., Q226L, T160A, and G186V in HA) that shift receptor binding preference towards human-type α 2,6-sialic acid receptors from the avian-type α 2,3-sialic acid receptors[19, 20].

[19] Gao, R. et al. Human infection with a novel avian-origin influenza A (H7N9) virus. N Engl J Med 368, 1888–1897 (2013).

[20] Xiong, X. et al. Receptor binding by an H7N9 influenza virus from humans. Nature 499, 496-499 (2013).

[39] Zhang, Q. et al. H7N9 influenza viruses are transmissible in ferrets by respiratory droplet. Science 341, 410-414 (2013).

[40] Long, J. S. et al. Species difference in ANP32A underlies influenza A virus polymerase host restriction. Nature 529, 101-104 (2016).

14. Page 24 - presumably it was human mediated activity such as trade that accelerated the geographic spread of the virus, not humans as hosts themselves? Suggest that you re-write this part.

Response 14:

We thank the reviewer for the comment. We agree that our original sentence could have been misinterpreted to suggest that human-to-human transmission was the driver of the high diffusion coefficient. It is important to distinguish between humans as hosts and human-mediated activities.

We have revised the sentence to clarify that the accelerated geographic spread of the virus is likely due to human activities, such as trade and travel, rather than humans serving as hosts.

Change in the text (the Discussion section):

[Page 19, Lines 391-394]

In the current study, the weighted diffusion coefficient was much greater than that of Galliformes, which may reflect human-mediated activities, such as trade or travel, accelerating the geographic spread of the virus rather than humans as hosts themselves.

15. Page 25 - correlated predictors in GLM - can you pre-select the least correlated set of variables and just run with those rather than all of them ?

Response 15:

We sincerely appreciate this insightful comment. We fully agree with the important considerations regarding the relevance of predictors. In response to this recommendation, we implemented a rigorous two-step protocol:

First, we calculated Spearman's rank correlation coefficients for potential predictors (Supplementary Figure 21). The results indicated moderate correlation between GDP per capita and other variables (GDP per capita and airline passenger volume: $r = 0.54$; GDP per capita and sample size: $r = 0.47$; GDP per capita and temperature: $r = -0.65$). Therefore, GDP per capita was considered to be removed for GLM analysis. All retained variables exhibit $r < 0.5$, well below the conservative threshold commonly adopted in previous study ($r < 0.7$)[a].

Second, we performed a variance inflation factor (VIF) analysis, which is the standard method for diagnosing multicollinearity. All VIFs were < 2.5 (1.07~2.18). This shows that the variables in the final model do not have serious multicollinearity problems, ensuring the stability of parameter estimation.

Overall, our refined approach is methodologically robust. Our variable selection process, which combines a stringent correlation threshold (absolute $r < 0.5$) and VIF analysis, aligns with and even exceeds the standards in related literature. For instance, Guinat et al. retained variables with $r < 0.7$ [a]. Furthermore, the number of predictors in our final model is comparable to that used in recent

high-impact research. The scale of our model, in terms of the number of predictors, is in line with the analytical framework established by a previous study of H5 avian influenza virus dynamics in Nature Communications[b]. This demonstrates that our model complexity is appropriate and in line with current best practices.

Following this rigorous selection process, we re-analyzed the GLM using the refined set of predictors. The updated results are presented in Figure 9. The conclusions remain consistent with our original analysis, reinforcing that distance, live poultry trade, and temperature are significant factors affecting global H7 virus transmission.

[a] Guinat, C. et al. Poultry farm density and proximity drive highly pathogenic avian influenza spread. Communications biology **8**, 1306 (2025).

[b] Li, Y.T., Ko, H.Y., Hughes, J. et al. From emergence to endemicity of highly pathogenic H5 avian influenza viruses in Taiwan. Nature Communications **15**, 9348 (2024).

Change in the text (the Result section):

[Page 11, Lines 236-240]

Spearman rank correlation analysis moderate correlation between GDP per capita and other variables (GDP per capita and airline passenger volume: r = 0.54; GDP per capita and sample size: r = 0.47; GDP per capita and temperature: r = -0.65) (Supplementary Fig. 21). The variance inflation factor did not identify multicollinearity. Following best practices for multicollinearity mitigation, GDP per capita was removed from the final model.

Change in the figure (the Supplementary information section):

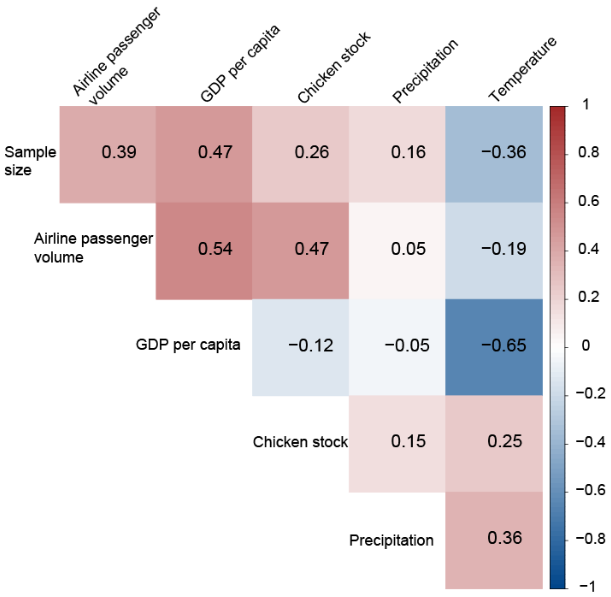

Supplementary Fig. 21. Correlation matrix of predictor variables for generalized linear model analysis. To adhere to a more stringent selection criterion, GDP per capita was excluded from the final model due to its moderate correlation with other variables (e.g., airline passenger volume, r = 0.54; temperature, r = -0.65). After the exclusion of GDP per capita, the absolute values of all remaining correlation coefficients are less than 0.5, indicating low inter-variable correlation and suitability for inclusion in the GLM.

Change in the figure (the Results section):

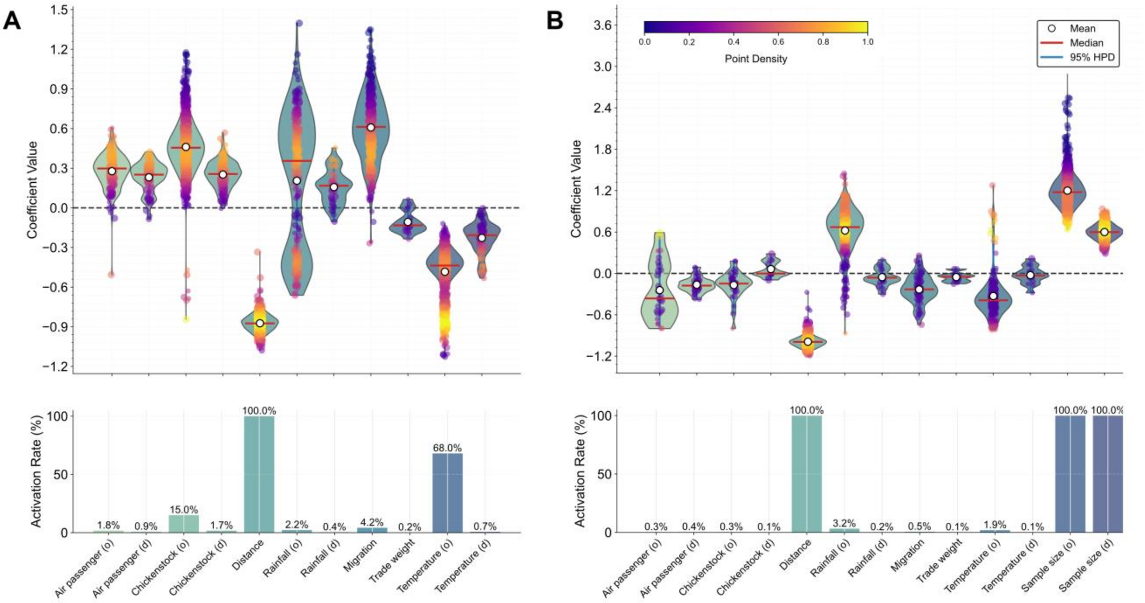

Fig. 9. Predictors of global H7 virus diffusion among five geographic regions.

Change in the text (the Methods section):

[Page 29, Lines 611-614]

Prior to GLM modeling, Spearman rank correlation analysis was performed among candidate predictors. Variables exhibiting |r| > 0.7 were flagged for potential collinearity. Subsequently, variance inflation factors (VIF) were calculated to identify significant multicollinearity.

[Page 29, Lines 627-637]

In interpreting the GLM results, the following criteria were applied to assess the importance and significance of predictors:

Activation rate (the frequency with which a predictor is included in the model) was used to gauge importance: an activation rate ⩾50% indicates a highly important predictor, 10-50% indicates moderate importance, and <5% suggests potential unimportance.

HPD intervals (Highest Posterior Density intervals) from the posterior distribution were examined for each coefficient: if the HPD interval does not contain zero, the effect is considered significant; if it contains zero, the effect is uncertain.

Coefficient magnitude was classified as: |coefficient| > 0.5 indicates a strong effect, 0.2 < |coefficient| < 0.5 indicates a moderate effect, and |coefficient| < 0.2 indicates a weak effect.

These standards ensure a structured evaluation of the predictors' roles in viral spread dynamics.

16. Supplementary Figure 6 and similar - please add the dataset labels for A, B, C, D in the panels and figure legend.

Response 16:

We sincerely appreciate the reviewer's careful attention to detail regarding figure labeling. We have now clearly labeled all panels (A, B, C, D) in Supplementary Figure 6 and updated the corresponding legend to improve clarity. Similarly, we have updated the legend in Supplementary Figure 5.

Change in the text (the Supplementary Figure section):

Supplementary Fig.5. The solid (blue) line represents the posterior median of the population size over time, while the upper and lower lines indicate the 95% highest posterior density (HPD) interval.

H7 (A), H7N3 (B), H7N7 (C), and H7N9 (D).

Supplementary Fig.6. MCC trees derived using Bayesian phylogenetic analysis, where the support rate of nodes is represented by posterior probability. Nodes with a posterior probability greater than 0.95 are considered highly reliable, while nodes below 0.75 have weaker support. H7 (A), H7N3 (B), H7N7 (C), and H7N9 (D).

17. The authors will make the datasets available on Zenodo but havent done so yet.

Response 17:

We thank the reviewer for raising this important point and apologize for any confusion caused. All datasets supporting the conclusions of this study have now been re-deposited in our GitHub repository (https://github.com/skylynf/H7_Dynamics). Should there be any difficulty in accessing the data, we would greatly appreciate the reviewer’s further feedback.

Reviewer #3:

In the paper "Disentangling the Drivers and Host-Mediated Global Spread of Influenza A (H7) Virus" the authors used Bayesian phylodynamic analysis to explore geographic dispersal of H7 and highlight the subtypes H7N3, H7N7, and H7N9 over time, along with exploring how host animal interacted with dispersal dynamics. We appreciated the cohesive and focused narrative throughout the results - which used appropriate methods to address relevant questions. Overall, this was an interesting paper with important implications. We have a few primarily concerns that we would like to see addressed.

Response:

We sincerely appreciate your positive comment on the scientific importance of our work on H7 avian influenza virus transmission dynamics. We are grateful for the opportunity to address the specific concerns raised, which we believe have significantly strengthened the manuscript. Below we provide a point-by-point response to each comment, with all corresponding changes colourfully highlighted in the revised manuscript. Thank you again for your comment on our manuscript, which has helped us to enhance the clarity and rigor of our work.

Major concerns

1. Wave front distance shown in top panels of Fig 7 is currently presented in a manner that is somewhat difficult to reconcile for the reader because the majority of travel occurs prior to the shown timeline. We feel that further discussion and interpretation would be beneficial for the reader to understand. Supplemental figures 8-11E and F provide meaningful context to Figure 7 and we would suggest incorporating them in the main text. This could be tied into earlier geographical divergence results (ex. line 119). Alternatively, Fig 7 could be reconfigured to present the final wave front distance, and not introduce the element of time, reflecting the current discussion in lines 238-260.

Response 1:

We sincerely thank the reviewer for these excellent suggestions, which have significantly improved the clarity of our results. We have implemented the reviewer's proposed solutions as follows:

First, we completely agreed that the original time-based presentation of wavefront distance was

challenging to interpret. As the reviewer aptly suggested, we have reconfigured Fig 7 to focus solely on the final wavefront distance, and have removed the temporal element (now new Fig 8). This new presentation aligns perfectly with the narrative in our manuscript, providing a clear summary of the maximum geographical extent of viral spread for each subtype and host, which is much more intuitive for the reader.

Second, we agree that the results from original Supplemental Figures 8-11E and F provide important context that enhances the interpretation of original Figure 7. Accordingly, these figures have been incorporated into the main text, and the relevant section has been revised. In addition, following another reviewer's suggestion, we have re-analyzed the dispersal velocity, wavefront distance, and spread coefficient for H7 viruses based on the two lineages and re-drawn a more detailed figure (now presented as the new Figure 5). We designated this as Figure 5 because the existing Figure 4 visually illustrates how and where the virus spread, while the new Figure 5 provides the quantitative metrics (how fast and how far) that directly characterize these patterns. This sequential presentation greatly improves the reader's understanding of the complete transmission dynamics of H7 viruses.

We are grateful to the reviewer for these constructive recommendations. The manuscript is now substantially improved, with a more logical narrative and more accessible figures that better support our conclusions.

Change in the text (the Result section):

[Page 10, Lines 204-211]

Wavefront distance and diffusion coefficient were estimated using the continuous diffusion model (Fig. 8). Wavefront distance varied by host type: in the latest estimates, Anseriformes exhibited a wavefront distance of 12,096.978 km (7,901.05 - 12,096.978), exceeding that of Galliformes (9,609.90 km, 8,097.98 - 11,927.54) for H7 virus (Fig. 8A). The diffusion coefficient was consistently higher for viruses originating from Anseriformes than from Galliformes (Fig. 8B). The diffusion coefficient of H7 virus among wild and domestic birds were similar,. The variation trend of H7N3 and H7N9 diffusion coefficient is consistent, while the diffusion coefficient of H7N7 was higher in domestic birds than in wild birds for a period of time.

Change in the figure (the Result section):

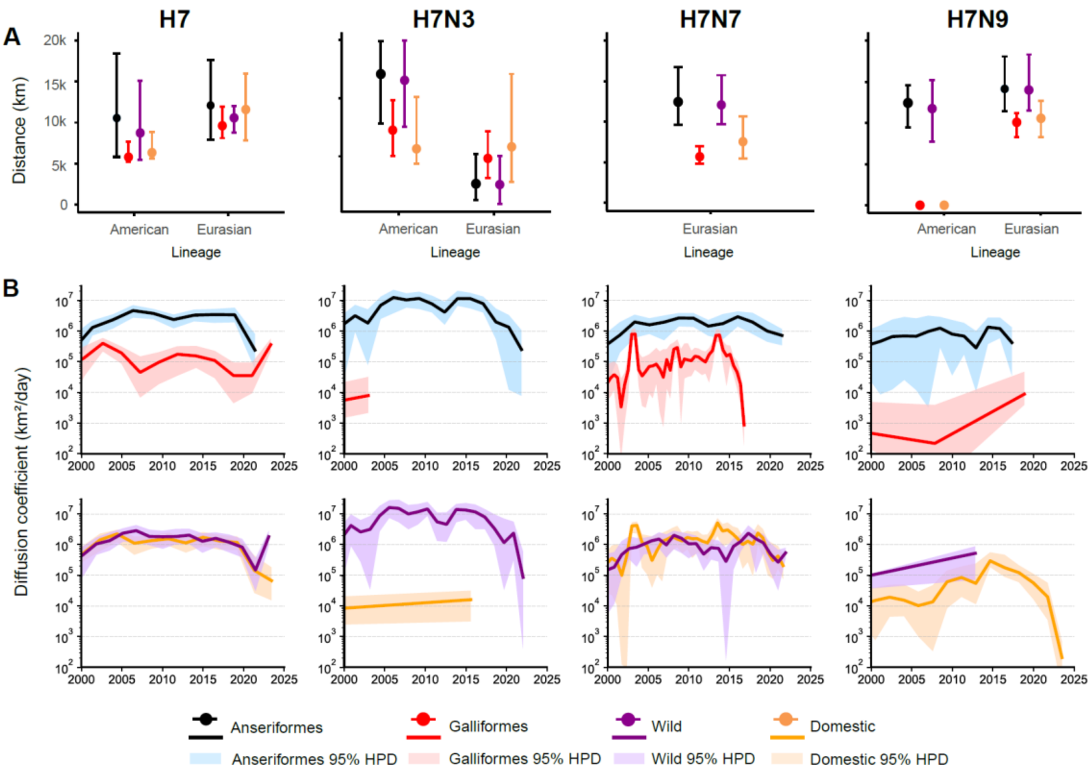

Fig. 8. Host-specific wavefront distance and diffusion coefficient estimates for H7 and its subtypes.

(A) Estimated wavefront distances (km) for H7 virus and subtypes. The wavefront distance represents the final extent of spatial spread; (B) Weighted diffusion coefficient (km²/day). Shaded areas represent 95% highest posterior density (HPD). Diffusion estimates for certain years are omitted due to irregular values likely resulting from limited sampling. Due to insufficient data, the American lineage of H7N7 was not included in the analysis.

Change in the text (the Result section):

[Page 8, Lines 159-169]

Furthermore, the spatial expansion of the Eurasian lineage and American lineage of H7 viruses in continuous space showed a median weighted branch dispersal velocity of 661.23 km/year (95% HPD: 400.12 - 733.47) and 354.18 km/year (260.13 - 444.50), respectively (Fig. 5). Similarly, the median dispersal velocities for the Eurasian lineage of H7N3, H7N7, and H7N9 subtypes were estimated at 585.37 km/year (514.10 - 648.87), 645.28 km/year (512.61 - 747.96), and 562.48 km/year (380.76 - 689.73), respectively (Supplementary Fig.8-10). Wavefront distance was defined as the mean great-circle distance from the epidemic origin to the most recently circulating lineages.

As of the latest estimate, the Eurasian lineage of H7 viruses exhibited a wavefront distance of 10,986.55 km (7,625.84 - 14,640.81), compared with 7,311.98 km (5,380.59- 11,971.32) for the American lineage (Fig. 5). The diffusion coefficient was consistently higher for the Eurasian lineage than for the American lineage.

Change in the figure (the Result section):

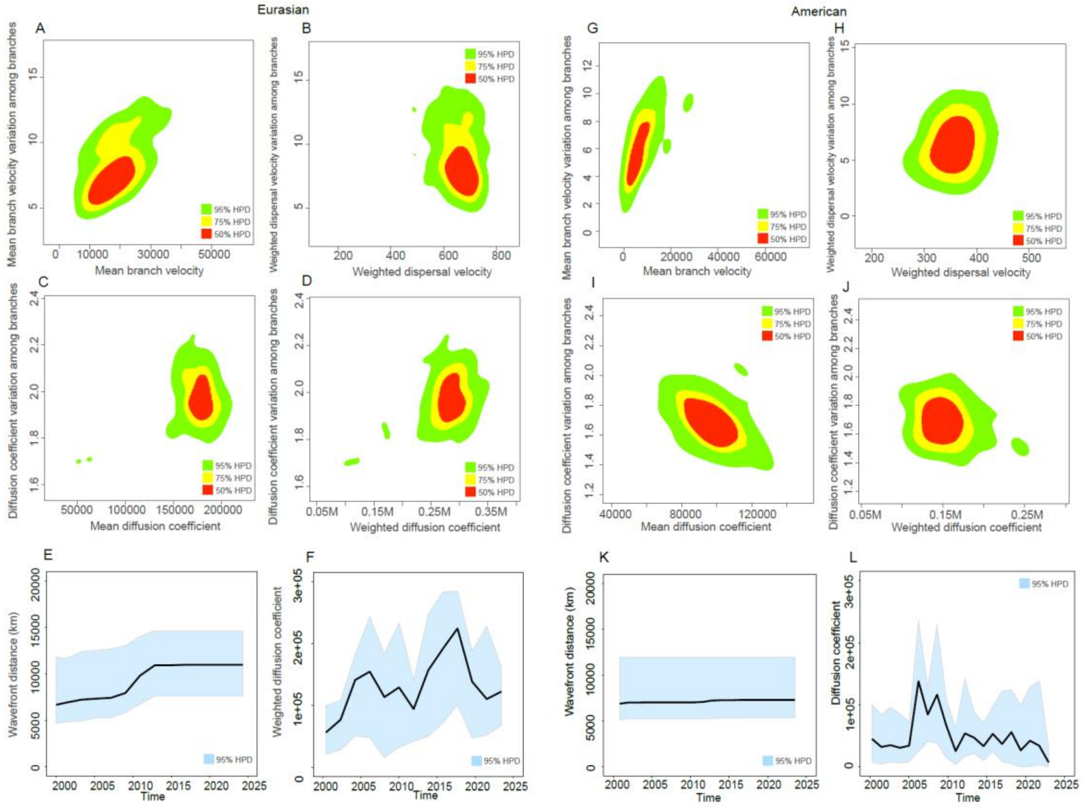

Fig. 5. Estimated dispersal statistics for H7.

The two columns on the left represent the Eurasian lineage, while the two columns on the right represent the American lineage. A and G, kernel density estimates of mean branch dispersal velocity parameters. B and H, kernel density estimates of weighted branch dispersal velocity parameters. C and I, kernel density estimates of original diffusion coefficient parameters. D and J, kernel density estimates of weighted diffusion coefficient parameters. E and K, furthest extent of epidemic wavefront (spatial distance from epidemic origin). F and L, Evolution of weighted diffusion coefficient.

2. The data and xmls have not been made available for review, that we have found.

Response 2: We thank the reviewer for raising this important point and apologize for any confusion caused. All datasets supporting the conclusions of this study have now been re-deposited in our GitHub repository (https://github.com/skylynf/H7_Dynamics). Additionally, the relevant XML files and supplementary data have also been made available in the repository to ensure complete transparency and reproducibility. Should there be any difficulty in accessing the data, we would greatly appreciate the reviewer's further feedback. We are committed to providing all necessary resources to facilitate thorough review and future research.

Minor concerns

1. For most figures it would be helpful to have the panels visually labeled in the figure (as was done fig 5), rather than only in the legend (as was done fig 2, 3, 4, 6, 7).

Response 1:

We sincerely thank the reviewer for this valuable suggestion to improve the clarity and immediate readability of our figures. Following the reviewer's advice, we have now revised all multi-panel figures to include clear labels within each panel. Due to revisions in the text, the figure numbering has been updated. Please refer to the current Figures 2, 3, 4, 7, and 8 in the manuscript for the revised versions.

2. Line 48 Galliformes are not introduced as was done for Anseriformes.

Response 2:

Thank you for your valuable suggestion. We have now added a definition of Galliformes and a brief description of its role in the Introduction section to parallel the introduction of Anseriformes.

Change in the text (the Introduction section):

[Page 3, Lines 53-56]

The Galliformes (pheasants, turkeys, peafowl, and quail) often exhibit synanthropic behavior and evidence has shown that many species in this order are susceptible to and can shed AIVs[13]. Galliformes have the potential to act as bridge hosts, as agricultural areas may attract wild individuals searching for food resources or conspecifics.

[13] Shriner, S. A. & Root, J. J. A Review of Avian Influenza A Virus Associations in Synanthropic

*Birds. Viruses **12**, 1209 (2020).*

3. Figure 1a has some ambiguity in the figure legends. It is unclear if the line plot or the bar plot refers to the left or right axis using only information in the figure/legend. Additionally with the density of the information it might be helpful to 1) separate the bar and line plot (allowing the authors to color code the lines as well as indicate by shape) and 2) to log scale the y axis. Although we will note we appreciate that vertically aligning the x-axis timeline is helpful for readability.

Response 3:

We sincerely thank the reviewer for these constructive suggestions to improve the clarity of Figure 1a. We have revised the figure accordingly.

First, we thank the reviewer for this helpful suggestion. We agree that separating the bar and line plots can improve clarity, allowing for better use of color and shape to distinguish the data. Accordingly, we have revised the figure to present the line (Figure 1A) and bar plots (Figure 1B) separately, with lines now color-coded and shapes indicated for easier interpretation.

Second, with regard to the logarithmic scaling of the y-axis, we observed that logarithmic scaling performed poorly on this dataset, particularly given the distinct phase characteristics of the H7 influenza outbreak. Using logarithmic coordinates would compress visual differences during these critical outbreak phases, making it difficult to effectively identify the distinct phases of epidemic development. We do not log the y-axis beacause the linear scale was able to truly reflect the absolute size difference of the three outbreak periods (2013-2014, 2016-2017 and 2023), and logging it will weaken the information about the outbreak. So we have retained this feature in the revised figure. Should there be any further comment on it, we would greatly appreciate the reviewer's further feedback.

Change in the figure (the Results section):

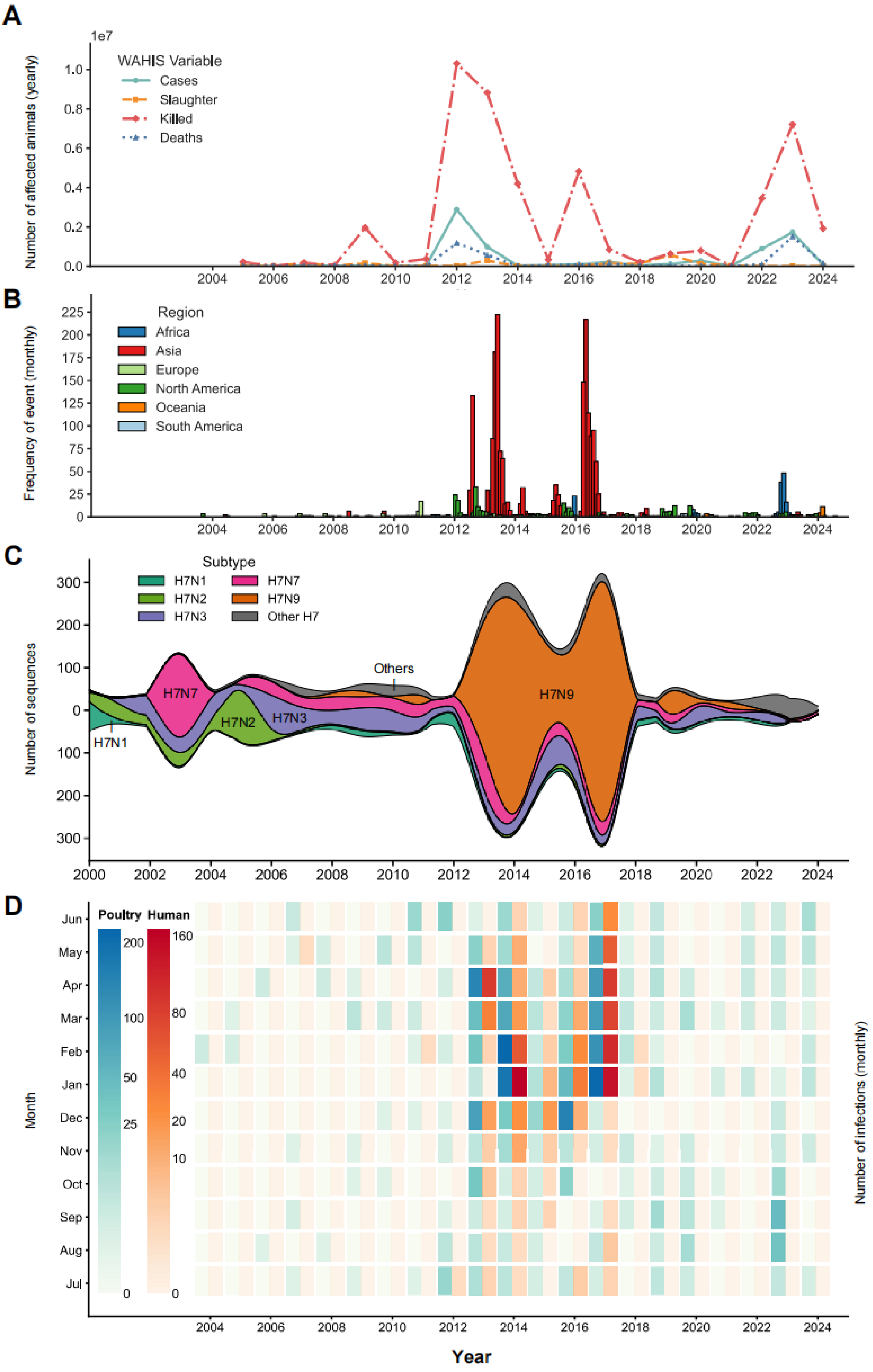

Fig. 1. H7 outbreak event and sequences.

(A) Annual number of affected asnimals from the World Animal Health Information System

(WAHIS). Cases refer to the number of confirmed outbreaks; Slaughter indicates the total number of animals slaughtered for outbreak management purposes (e.g. to prevent further transmission); Killed refers to animals actively culled to control the epidemic; Deaths represent the number of animals that died due to the disease. (B) Monthly time series of H7 outbreaks reports across all animal types by geographic region, as reported to the Food and Agriculture Organization of the United Nations (FAO). (C) Temporal trends of H7 HA sequences submitted to the GISAID and NCBI Influenza Virus Resource from January 2000 to November 2024. Time series describing H7 outbreaks in all animal types; the lower panel focuses on poultry-specific outbreaks. (D) Monthly number of H7 outbreaks in poultry (left y-axis) and confirmed human H7 infection cases (right y-axis), as reported to the FAO.

4. Supp Figures 5. and 6. do not specify which panel is associated with each subtype.

Response 4:

We sincerely appreciate the reviewer's careful attention to detail regarding figure labeling. We have now clearly labeled all panels (A, B, C, D) in Supplementary Figure 5 and updated the corresponding legend to improve clarity. Similarly, we have updated the legend in Supplementary Figure 6.

Change in the text (the Supplementary Figure section):

Supplementary Fig.5. The solid (blue) line represents the posterior median of the population size over time, while the upper and lower lines indicate the 95% highest posterior density (HPD) interval. H7 (A), H7N3 (B), H7N7 (C), and H7N9 (D).

Supplementary Fig.6. MCC trees derived using Bayesian phylogenetic analysis, where the support rate of nodes is represented by posterior probability. Nodes with a posterior probability greater than 0.95 are considered highly reliable, while nodes below 0.75 have weaker support. H7 (A), H7N3 (B), H7N7 (C), and H7N9 (D).

5. Line 528/Supp Figure 5, what are the time intervals used with estimating Ne?

Response 5:

We thank the reviewer for this detailed technical question. The time intervals for estimating Ne were set to 5 years according to the protocol by Hill & Baele (2021)[a], which provides a balanced resolution for identifying long-term trends in population dynamics:

By default, the Skygrid Analysis dialog box only requires the Age of youngest tip to be provided in order to provide a visualization corresponding to real time, as all the other fields are inferred automatically from the log file. The time points for Ne estimation are then automatically generated by the software at the specified interval (in our case, 5 years) across the time span of the phylogeny. We have added these details to the Methods section for clarity.

Change in the text (the Methods section):

[Page 24, Lines 514-517]

The resulting visualizations depict fluctuations in the Ne of virus across different time periods, with a time interval of 5 years by default settings, and can be analyzed and visualized by Tracer version 1.7.2.

[a] Hill, V. & Baele, G. Bayesian Estimation of Past Population Dynamics in BEAST 1.10 Using the Skygrid Coalescent Model. Mol Biol Evol 36, 2620–2628 (2019).

6. Line 111-113 is not a complete sentence.

Response 6:

Thank you for pointing out this oversight. We apologize for the incomplete sentence. We have now revised and completed the sentence to ensure it is grammatically correct.

Change in the text (the Results section):

[Page 6, Lines 110-112]

Globally, discrete trait phylogeographic analysis of the H7 HA gene segment showed predominantly unidirectional evolution.

7. Fig 2. It is unclear why the deeper internal branches are not colored. Was there a threshold for support, if so that should be discussed.

Response 7:

We thank the reviewer for this critical observation regarding Fig 2. In our original figure, the lack of color in the deeper internal branches was not due to a support threshold but was a default characteristic of the geographical trait mapping in the Chiplot tool we used.

To address this point comprehensively, we present the results of MCC tree using FigTree software. All branches, including the deeper internal ones, are now colored according to the inferred geographic origin of their descendant lineages (see Temporary Figure 1). This will provide a complete visualization of spatial spread of the viruses.

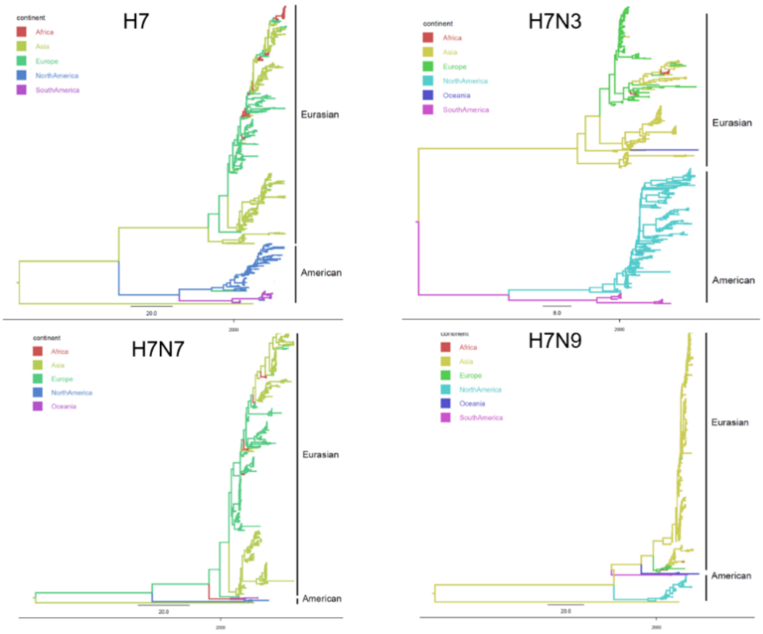

Temporary Figure 1. Time-scaled maximum clade credibility (MCC) tree of H7 HA genes sequences plotted by FigTree software, with branch colors representing geographic information.

To overcome FigTree's limitation in concurrent data visualization (e.g., host, pathogenicity, subtype, lineage), we still selected the online tool Chiplot to achieve a more comprehensive visualization. This time, we displayed the geographic information in the end branch, and the result was the same as the one drawn by Figtree. Based on the geographic result, we can divide the virus into Eurasian and American lineages (Fig. 2).

We believe the new figure is a significant improvement and greatly enhances the interpretability of our phylogenetic results.

Change in the text (the Results section):

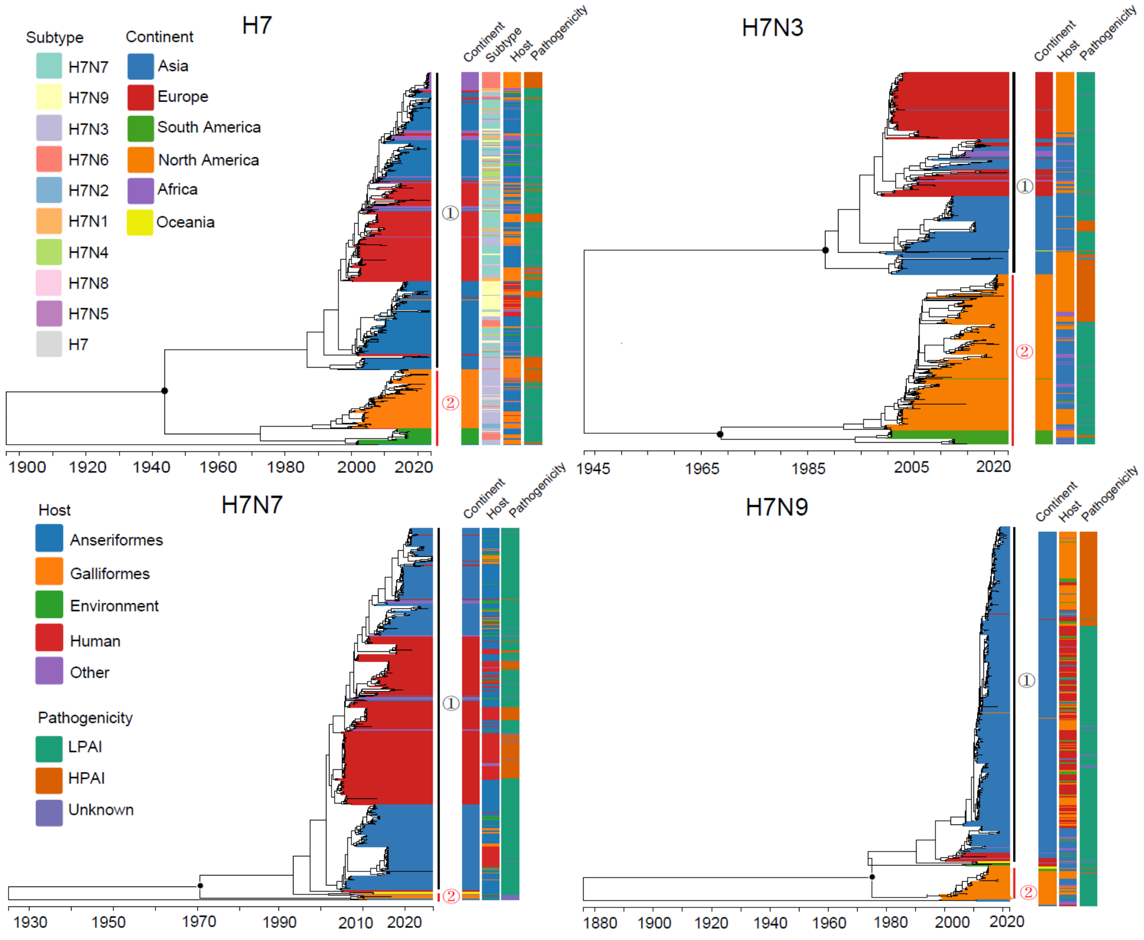

Fig. 2. Time-scaled maximum clade credibility (MCC) tree of H7 HA genes sequences.

The evolutionary relationships of the H7 HA gene segment were reconstructed using full-length sequences from viruses collected across different geographic regions. The geographic information was displayed in the end branch. The sequences in HA lineage □ were mainly from Asia and Europe, so we named □ as Eurasian lineage. The sequences in HA lineage □ were from America, so we named □ as American lineage.

8. Line 120-125. Including the uncertainty in the time estimates of divergence events would be informative.

Response 8:

Thank you for your valuable suggestion. We fully agree that reporting the uncertainty in divergence time estimates is essential. We have now added the 95% Highest Posterior Density (HPD) intervals for all key evolutionary events.

Change in the text (the Results section):

[Page 6, Lines 118-126]

Notably, the Maximum Clade Credibility (MCC) tree of the H7 HA gene showed that major geographical divergence began around 1943 (95% highest posterior density [HPD]: 1902 - 1969) (Fig. 2A). For H7N3, two distinct lineages have circulated in South America, North America; Asia, and Europe, with divergence times estimated in 1970 (1952 - 1985) and 1989 (1977 - 1994), respectively (Fig. 2B). In the case of H7N7, geographical separation was first observed in 1969 (1940 - 1988) and has predominantly persisted in Europe and Asia since 2000 (Fig. 2C). As for H7N9, the MCC tree indicated Asia as the most probable origin and dominant region of circulation, with limited evidence of cross-regional spread. A divergence event with North America lineages is estimated to have occurred in 1976 (1966 - 1987).

9. Figure 5. The direction arrow heads are indistinct and can be easily mistaken as dots, making them fully opaque or outlined could improve readability.

Response 9:

Thank you for your thoughtful comments. We agree completely that the arrowheads were ambiguous and could be misinterpreted. We have now modified all direction arrows in the figure 5 (now Fig.6) as follows: (1) The arrowheads are now set to 100% opacity (fully opaque); (2) A solid black outline has been applied to each arrowhead to sharply define its shape against the background. (3) We have also slightly increased the size of the arrowheads for better visibility.

Change in the figure (the Results section):

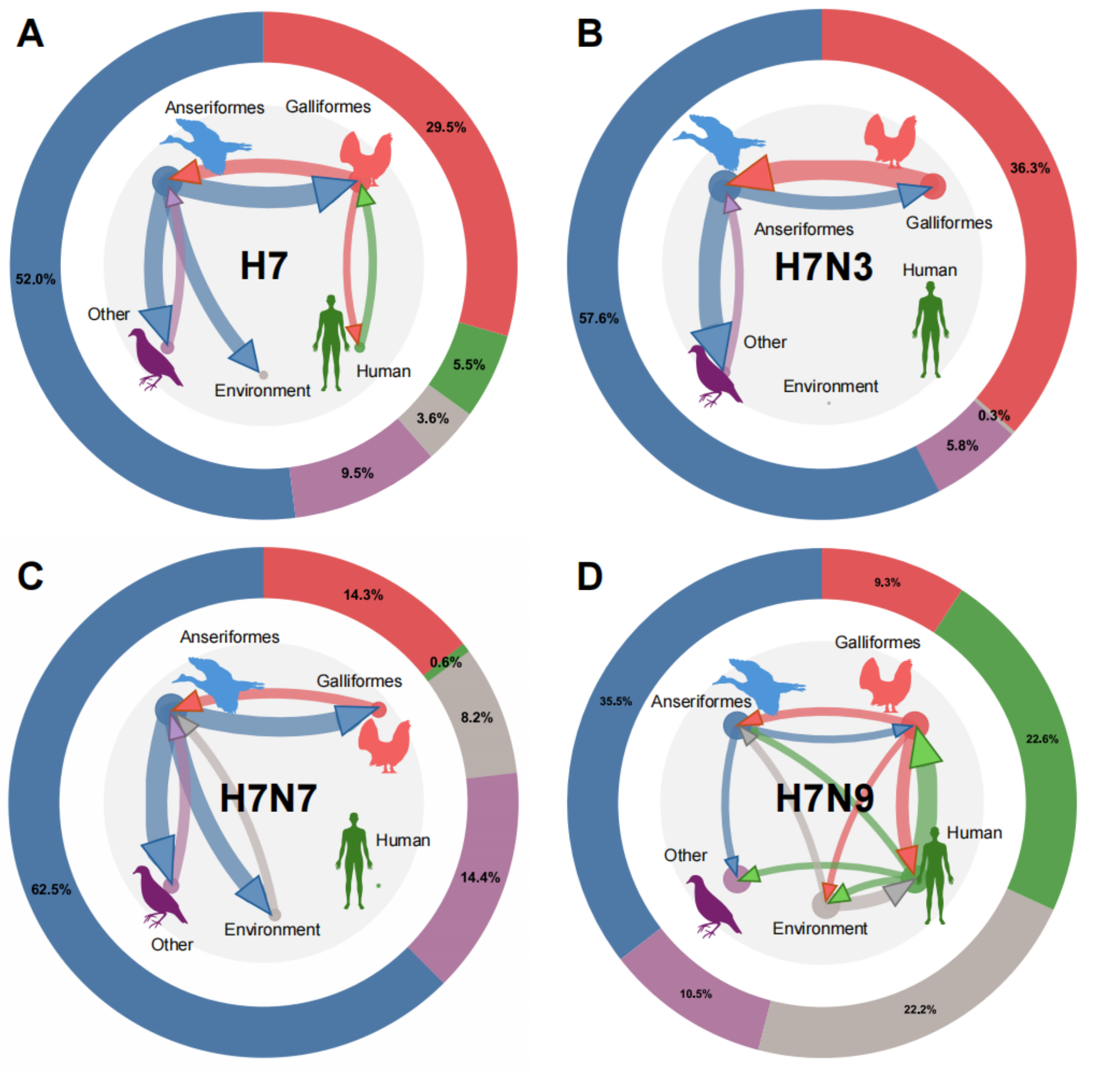

Fig. 6. Contrasting host transmission patterns of H7 subtypes inferred from discrete-trait phylogenetic analysis.

10. Line 200. It initially appears that these are a separate category with non-overlapping samples from the Galliformes and Anseriformes. Introducing that all samples from bird hosts were partitioned into wild vs domestic here would improve readability.

Response 10:

Thank you for this excellent suggestion. We agree that the initial description was ambiguous and could lead to the misinterpretation that 'wild' and 'domestic' birds were a separate category with non-overlapping samples from the Galliformes and Anseriformes. To improve clarity, we have revised the manuscript to explicitly state that all avian samples were categorized based on their wild or

domestic status. This distinction is crucial for our analysis, and we appreciate your comment, which has significantly improved the readability and clarity of this part.

Change in the text (the Results section):

[Page 9, Lines 185-186]

We also analyzed the transmission dynamics of hosts, classified as either wild or domestic, which included samples from Galliformes and Anseriformes.

11. Line 210. Was there a statistical test done to show statistical significance of the difference?

Response 11:

Thank you for your thoughtful comments. In response, we have now performed formal statistical testing to quantify the significance of observed differences between groups. Shapiro-Wilk tests confirmed both groups deviated significantly from normality (Anseriformes and Galliformes, $P < 0.01$; wild and domestic, $P < 0.01$ for H7 virus and subtypes). And then we conducted a Mann-Whitney U test to compare the dispersal velocities between Anseriformes and Galliformes host groups as well as wild and domestic birds group. The result showed the dispersal velocity of H7 virus and subtypes was significantly faster among Anseriformes than Galliformes ($P < 0.01$). Additionally, wild birds exhibited significantly higher dispersal velocity than domestic poultry in H7, H7N3, and H7N9 ($P < 0.01$); however, domestic birds contributed more to the rapid spread of Eurasian lineage of H7N7 ($P < 0.01$).

Change in the text (the Results section):

[Page 10, Lines 193-199]

The Eurasian lineage of H7 virus spread more rapidly among Anseriformes (586.51 km/year, 518.90 - 682.14) and wild birds (654.60 km/year, 595.14 - 751.59) than among other hosts, whereas Galliformes exhibited the slowest dispersal velocity (294.44 km/year, 253.42 - 343.79) (Fig. 7). This pattern was consistent across subtypes: Anseriformes displayed significantly higher dispersal velocity than Galliformes in H7N3 (Mann-Whitney U test, $P < 0.01$) , H7N7 ($P < 0.01$), and H7N9 ($P < 0.01$) (Supplementary Fig. 11-13; Supplementary Table 9). Wild birds generally transmitted viruses faster than domestic poultry in H7, H7N3, and H7N9 ($P < 0.01$), whereas in the Eurasian lineage of H7N7, domestic birds contributed more to the rapid transmission than wild birds (P <

0.01) (Supplementary Figs. 11-13, Supplementary Table 9).

Change in the text (the Supplementary material section):

Supplementary Table 9. The comparison of the weighted branch dispersal velocity (median value, 95% HPD) using Mann-Whitney U test.

Subtype	*Anseriformes*	*Galliformes*	*W*	*P*	*Wild*	*Domestic*	*W*	*P*
H7-Eurasian lineage	*586.51 (518.91, 682.14)*	*294.44 (253.42, 343.79)*	*10000*	*<0.001*	*654.60 (595.14, 751.59)*	*581.32 (478.06, 653.14)*	*1016*	*<0.001*
H7-American lineage	*189.66 (112.79, 929.07)*	*8.34 (2.29, 19.94)*	*10000*	*<0.001*	*408.41 (304.88, 511.47)*	*172.85 (87.74, 513.66)*	*167*	*<0.001*
H7N3-Eurasian lineage	*594.388 (516.63, 691.75)*	*2.94 (1.30, 10.37)*	*10000*	*<0.001*	*677.24 (560.01, 805.16)*	*43.02 (22.82, 305.56)*	*0*	*<0.001*
H7N3-American lineage	*1561.18 (1293.88, 1881.26)*	*3.15 (1.46, 5.91)*	*10000*	*<0.001*	*1602.18 (1274.80, 1859.94)*	*3.70 (2.23, 9.42)*	*0*	*<0.001*
H7N7-Eurasian lineage	*621.22 (503.67, 707.20)*	*346.68 (271.21, 429.61)*	*9999*	*<0.001*	*507.47 (416.94, 609.94)*	*766.87 (631.36, 943.98)*	*9982*	*<0.001*
H7N9-Eurasian lineage	*338.39 (205.39, 497.05)*	*12.10 (7.49, 17.44)*	*10000*	*<0.001*	*275.37 (105.04, 451.31)*	*56.62 (34.74, 122.20)*	*87*	*<0.001*
H7N9-Eurasian lineage	*1444.47 (201.08, 2198.11)*	*4.36 (2.68, 6.51)*	*10000*	*<0.001*	*296.41 (54.74, 760.83)*	*4.90 (3.05, 7.52)*	*0*	*<0.001*

12. Line 265. The word "barrier" and phrase "positive contribution to the virus spread" implies some

level of inferred causality, which has not been shown. "Correlated" and "associated" do not have this issue.

Response 12:

Thank you for your insightful suggestion. We fully agree that our original wording inappropriately implied a causal mechanism, which cannot be definitively concluded from our correlation analysis alone. We have carefully revised the manuscript to replace all causal language with terms “associated” that accurately describe the statistical associations observed in our models.

Change in the text (the Results section):

[Page 12, Lines 240-246]

Geographic distance was strongly negatively associated with viral spread (coefficient = -0.873, 95% HPD: -0.995 to -0.753), with decisive support for its inclusion in the model (activation rate =1) (Fig. 9A). Chicken stock origin, defined as the number of annual chicken inventory at the source country, was consistently positively associated with virus spread (0.461, 0.111 to 0.845), while temperature origin (-0.484, -0.900 to -0.237) and temperature destination (-0.227, -0.499 to -0.047) was also considered as a negative and supportive predictor.

13. At lines 267 and 268-9 defining chicken stock and virus sample size, respectively could improve clarity of the text.

Response 13:

We thank the reviewer for this helpful suggestion. We have now added explicit definitions for both variables in this to enhance clarity.

Change in the text (the Results section):

[Page 12, Lines 243-248]

Chicken stock origin, defined as the number of annual chicken inventory at the source country, was consistently positively associated with virus spread (0.461, 0.111 to 0.845), while temperature origin (-0.484, -0.900 to -0.237) and temperature destination (-0.227, -0.499 to -0.047) was also considered as a negative and supportive predictor. Virus sample size, number of H7 sequences included per discrete location at both origin and destination locations, was positively associated with viral transmission.

14. Figure 9. A visual color legend in the figure, instead of only describing in the legend text, would improve interpretability. It would also be helpful to use referential color schemes for the subtypes and outbreaks (eg. If H7N9 is red then use light and dark red arrows for 2013/2014 and 2016/2017 and blue or orange for 2023). Overall figure 9 is a helpful, elegant summary figure.

Response 14:

Thank you for this positive feedback and for the constructive suggestions. We agree that a visual color legend and a more referential color scheme will significantly enhance the figure's interpretability. **First**, we have added a clear visual color legend directly onto the figure itself, so readers can immediately interpret the colors without referring back to the text caption. **Second**, we have used triangles with significant color differences to denote outbreaks summarized from the World Animal Health Information System (WAHIS) for three key time periods. Arrows representing viral spread pathways inferred from Markov jump estimates are color-coded based on sequences. These changes significantly enhance the figure's readability while maintaining its elegant design.

Change in the figure (the Results section):

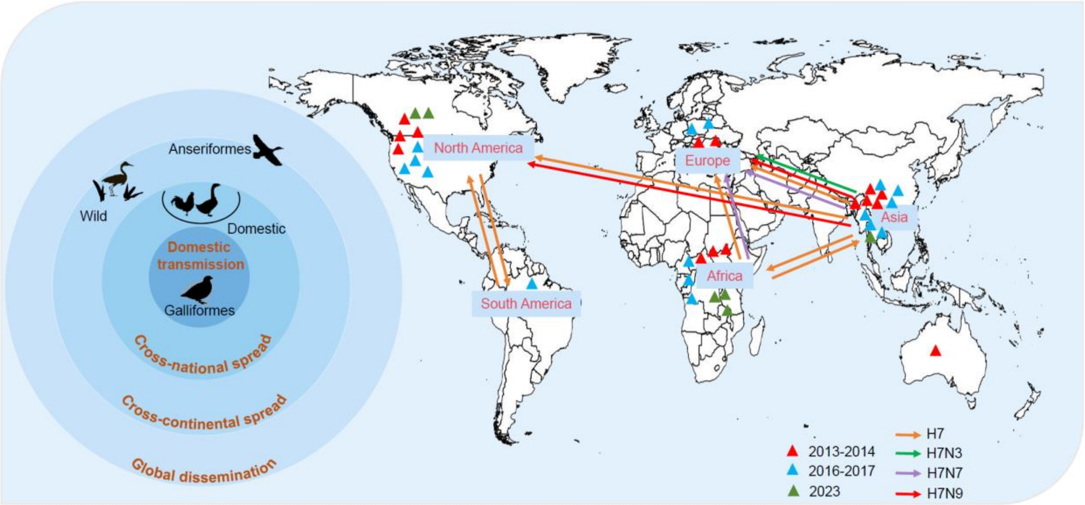

Fig. 10. Graphical summary of the global transmission dynamics of H7 avian influenza virus.

This figure illustrates key large-scale outbreaks of H7 viruses, including those in poultry, with major events marked in different colored triangles. The number of triangles on each continent corresponds to its ascending rank in outbreak frequency during the same period (e.g., □=lowest, □□=intermediate, □□□=highest). The concentric circles on the left show the effect of different

hosts on geographic spread. These colored arrows do not represent literal geographic paths but instead indicate directionality of intercontinental spread according to the result of Markov jumps.

15. Line 507-509 there is a lack of detail in this process, was there a threshold used, how many sequences were removed?

Response 15:

Thank you for this excellent suggestion. We agree that providing more detail on the process of filtering molecular clock outliers is crucial for reproducibility. Treetime is a program that calculates these values in advance by calibrating the molecular clock to the sampling time of each sequence, so it can be used to see if the sequence has a temporal structure. We run this software on a Linux system, and the command is as follows:

treetime --dates *.csv --aln *.fas --tree *.nwk --outdir xxfolder

TreeTime's default algorithm identifies outliers as those sequences whose residual deviation from the regression line exceeds a threshold based on the distribution of all residuals (see https://github.com/neherlab/treetime). Specifically, the algorithm first calculates the residual for each terminal node using the formula: Residual = The distance from the root of the node to the node - (clock_rate * mean_date_constraint) - intercept, representing the difference between the observed and expected evolutionary distances. Then the algorithm computes the interquartile distance (IQD) of all residuals. Sequences are flagged as outliers if their absolute residual exceeds 3 * IQD.

The software automatically produces a root-to-tip divergence plot where sequences that significantly deviate from the temporal regression line are highlighted in red by default and corresponding result table. In our study, there are a total of 58 sequences that need to be excluded, leaving 814 sequences for further analysis. In order to demonstrate more clearly how to exclude these sequences, the initial results of Treetime have been added to the Methods in the revised manuscript and Supplementary material.

Change in the text (the Methods section):

[Page 23, Lines 487-495]

The specific filter method can be found at https://github.com/neherlab/treetime. Specifically, the method first calculates the residual for each terminal node using the formula: Residual = The

*distance from the root of the node to the node - (clock_rate * mean_date_constraint) - intercept, representing the difference between the observed and expected evolutionary distances. Then the algorithm computes the interquartile distance (IQD) of all residuals. Sequences are flagged as outliers if their absolute residual exceeds 3 * IQD. This robust statistical approach, based on the IQD, ensures that the threshold remains unaffected by extreme values, thereby maintaining the reliability of outlier identification in molecular clock analysis.*

Change in the Supplementary material (the Supplementary figure section):

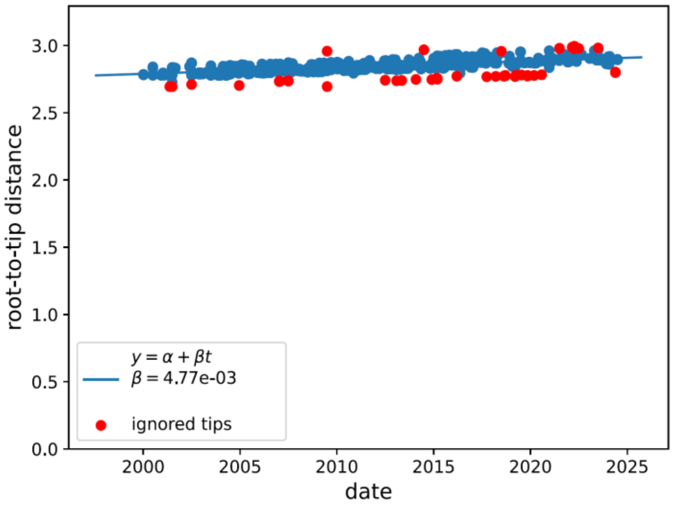

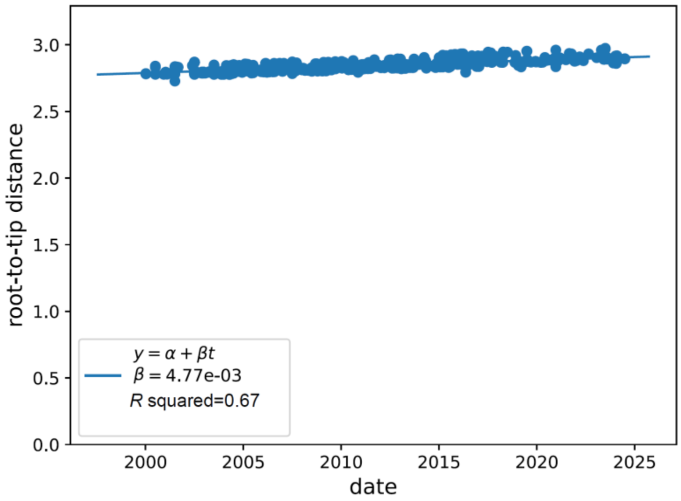

Supplementary Fig.4. Strong temporal signal tested in Treetime of HA genes of H7 (R squared=0.67). The initial result of Treetime (Top). The red dots represent that this sequence does not have a time structure, and there are a total of 58 sequences that need to be excluded (Bottom).

16. Line 551. A supplementary table that specified what indicated domestic vs wild would improve the reproducibility of the paper, but we would not require it.

Response 16:

We thank the reviewer for this excellent suggestion to improve the clarity of our host classification. We agree that explicitly defining the criteria for distinguishing between domestic and wild birds is important. We have now added a description in the Methods section to summarize these criteria.

Change in the text (the Methods section):

[Page 25, Lines 536-541]

Furthermore, based on the sequence names and host information, the bird hosts were categorized into domestic and wild groups, according to the following criteria: (1) host species explicitly designated as "poultry" or "farm" in sequence metadata (such as "chicken", "domestic duck") and host species known to be primarily commercially reared (e.g., Gallus gallus domesticus) are considered domestic; (2) host species typically considered wild reservoirs (e.g., 'mallard', 'teal', 'wild bird') were classified as wild.

Reviewer #3 (Remarks on code availability):

17.We have not been able to find any code. The authors say the data XMLs have been uploaded to zenodo and will be available upon publication, however we have not seen them, nor been able to reproduce the analyses. Data and reproducible analyses must be made available for further review.

Response 17:

We sincerely thank the reviewer for raising this critical point regarding code and data availability. We apologize for any confusion and appreciate the opportunity to clarify. We also advocate for reproducible research and have prepared all necessary materials. The original data, custom BEAST XML configuration files, R scripts for post-processing and visualization, and the curated dataset have already been uploaded to github repository: https://github.com/skylynf/H7_Dynamics. Should there be any difficulty in accessing the data, we would greatly appreciate the reviewer’s further feedback.

Reviewer #4 (Remarks to the Author):

I co-reviewed this manuscript with one of the reviewers who provided the listed reports. This is part of the Nature Communications initiative to facilitate training in peer review and to provide appropriate recognition for Early Career Researchers who co-review manuscripts.

Response:

We sincerely appreciate the time and expertise that the co-reviewers have dedicated to evaluating our manuscript. We fully support Nature Communications' initiative to foster peer review training and recognize the contributions of Early Career Researchers in the scientific evaluation process.

REVIEWER COMMENTS

Reviewer #1 (Remarks to the Author):

I would like to express my appreciation to the authors for their thoughtful and comprehensive responses to the my comments, which have effectively addressed all of my questions.
The revised manuscript has been substantially improved, demonstrating significant enhancements in methodological rigor, analytical robustness, and overall clarity, with conclusions reasonably supported by the presented evidence. The findings represent a valuable contribution, with formative insights that critically inform the development of robust public health readiness and operational response protocols for avian influenza pandemics.
I recommend its acceptance for publication.
Response: We sincerely thank you for your positive evaluation and recommendation for publication. We are grateful for your insightful comments throughout the review process, which have significantly strengthened the manuscript.

Reviewer #2 (Remarks to the Author):

Many thanks for considering and replying to my comments of the first round. I think your revised version now has much improved analysis and interpretation; and you have addressed the points I raised sufficiently well. I think the new analyses splitting by host type and hpai/lpai is much better and shows more clearly the details of the H7's transmission patterns.

1. New minor points:
However, there are now a couple of new minor points, to reword some sentences, which require attention -

Page 2 Lines 37-47 "Historically, outbreaks caused by high-pathogenic avian influenza (HPAI) viruses have mostly arisen from virulence shifts by low-pathogenic avian influenza (LPAI) viruses" - This is true, but I think it might be misinterpreted, for example the recent H5 clade 2.3.4.4b epizootic outbreaks, although 'historically' these did arise from a LPAI to HPAI event in 1996 (Gs/Gd Lineage), they are not really referred to as a single outbreak now since the H5 2.3.4.4b viruses are widespread amongst continents, countries, species and years (2014 onwards). So suggest re-wording to talk about origin of clades or lineages ? perhaps
"Historically, the major high-pathogenic avian influenza (HPAI) virus lineages are known to have originated from previously low-pathogenic avian influenza (LPAI) strains" [give reference to a review paper, e.g. your reference 6].
Response: We sincerely thank you for your thoughtful and constructive comments. The sentence has been revised to what you suggest in the revised manuscript. And we have added a reference which investigated and summarized the literature on emerging HPAI H5 and H7 viruses with the aim of building a spatio-temporal database of all these recorded LPAI-to-HPAI conversion events.
Change in the text:

[Page 2, Lines 37-39]

Historically, the major high-pathogenic avian influenza (HPAI) virus lineages are known to have originated from previously low-pathogenic avian influenza (LPAI) strains[6].

[6] Dhingra, M. S. et al. Geographical and Historical Patterns in the Emergences of Novel Highly Pathogenic Avian Influenza (HPAI) H5 and H7 Viruses in Poultry. Frontiers in veterinary science 5, 84 (2018).

"Notably, HPAI H5 virus has demonstrated unprecedented pandemic potential, by becoming endemic in bird reservoirs and causing widespread infections across 84 countries in Asia, Africa, Europe, and North America (ref 6)" - possibly true but misleading as written; pandemic potential usually refers to human pandemic potiential, so it is unclear how 'endemic in wild bird reservoirs' would cause this. I think you are meaning to refer to the recent H5 2.3.4.4b epizootic here, so I think it would be better to specific. Suggest something more like:
"Notably, a major lineage of HPAI H5, clade 2.3.4.4b viruses, are the cause of an epizootic in wild bird reservoirs with widespread infections across 84 countries in Asia, Africa, Europe, and North America (ref 6)" [suggest including some other references from your list also].
Response: Thank you for this excellent suggestion. The sentence has been revised to what you suggest in the revised manuscript. And we have added a reference which introduces the evolution, spread and impact of HPAI H5 virus.
Change in the text:
[Page 3, Lines 39-41]
Notably, a major lineage of HPAI H5, clade 2.3.4.4b viruses, is the cause of an epizootic in wild bird reservoirs with widespread infections across 84 countries in Asia, Africa, Europe, and North America[7,8].

[7] Lee, D. H., Criado, M. F. & Swayne, D. E. Pathobiological Origins and Evolutionary History of Highly Pathogenic Avian Influenza Viruses. Cold Spring Harbor perspectives in medicine 11, a038679 (2021).
[8] Bellido-Martín, B. et al. Evolution, spread and impact of highly pathogenic H5 avian influenza A viruses. Nat Rev Microbiol 24, 45-60 (2026).

This sentence is also problematic - (part 1)
"The mechanisms by which LPAI viruses of the H5 and H7 subtypes evolve into HPAI viruses involve mutations, insertions, and recombination events occurring at the proteolytic cleavage site of the hemagglutinin (HA) protein" - really ? I thought the main mechanism was acquisition of a multi basic cleavage site between HA1 and HA2, which is mostly insertion rather than mutation or recombination. Please give references for the H5 and H7 mechanisms.
Response: We appreciate the opportunity to improve the clarity and precision of our manuscript. You are correct that the primary mechanism by which LPAI H5 and H7 viruses acquire high pathogenicity is through the acquisition of a polybasic hemagglutinin (HA) cleavage site, and this most commonly occurs via insertion events—such as tandem duplications of nucleotide sequences

encoding basic amino acids—rather than point mutations or recombination. For example, in H5 viruses, the emergence of HPAI from LPAI precursors has repeatedly involved insertions creating motifs such as PQRERRRKKR ↓ G.

Change in the text:

[Page 3, Lines 41-43]

LPAI viruses of the H5 and H7 subtypes can evolve into HPAI viruses through the acquisition of a multi-basic cleavage site at the HA1–HA2 junction, a process that most commonly occurs via insertion events[9,10].

[9] Fouchier, R.A., et al. Avian influenza A virus (H7N7) associated with human conjunctivitis and a fatal case of acute respiratory distress syndrome. Proc Natl Acad Sci USA 101, 1356-1361 (2004).

[10] Kwon, T., et al. Gene editing of pigs to control influenza A virus infections. Emerging Microbes Infect 13, 2387449 (2024)..

(part 2) "playing a critical role in mediating the virus's attachment to host cell receptors, thereby initiating the viral infection cycle (ref 6)". This a bit misleading, this part of the sentence makes it sound like the cleavage site is the same as the receptor binding site, which it is not. (And note that in the HA 2.3.4.4b the receptor binding site is avian-like, not human-like, hence the issue with the earlier sentence on 'pandemic potential'...)

Response: Thank you for pointing out this important clarification. We have revised the sentence as suggested to clearly distinguish the cleavage site from the receptor-binding site, and to note the avian-like receptor specificity of currently circulating H5N1 2.3.4.4b viruses, as supported by a recent study (e.g., Yamaji et al., 2020).

Change in the text:

[Page 3, Lines 43-46]

Notably, the region of cleavage site at the HA1–HA2 junction is distinct from the receptor-binding site; with respect to receptor binding, currently circulating H5N1 clade 2.3.4.4b viruses retain avian-like receptor specificity, limiting their immediate pandemic potential despite high virulence in birds[11].

[11] Yamaji, R. et al. Pandemic potential of highly pathogenic avian influenza clade 2.3.4.4 A(H5) viruses. Rev Med Virol 30, e2099 (2020).

[Page 3, Lines 60-62]

"However, the roles of different host groups in the global dissemination and cross-species transmission of H7 subtypes remain poorly understood, and it is still unclear how fast the LPAI and HPAI H7 viruses spread among diverse host populations" - great to analyse the spread in different host groups, but suggest that you re-word this sentence to be positive.

Response: Thank you for this constructive feedback. We have rephrased the sentence to be more positive and forward-looking.

Change in the text:

[Page 4, Lines 61-64]

To better understand the roles of different host groups in the global dissemination and cross-species transmission of H7 subtypes, particularly regarding the differential transmission rates of LPAI and HPAI H7 viruses, a systematic analysis of transmission patterns across different host groups is needed.

[Page 17, Lines 366-381]
"The differential dispersal velocities between LPAI and LPAI" - typo, one of the LPAI should be HPAI
Response: We appreciate your careful reading of the manuscript. The typo has been corrected; the sentence now properly contrasts HPAI and LPAI dispersal velocities.
Change in the text:
[Page 18, Lines 388-389]
The differential dispersal velocities between HPAI and LPAI provided critical implications for understanding H7 transmission.

2: About the revised analysis and figure changes - thanks for considering my comments, and I'm pleased that you were able to improve the manuscript, all these changes look good and answer my original points. Specifically -

Response 7: many thanks for including the continent information and alternations to the figures
Response 8: good, thanks for updating the analysis
Response 9: (analysis of individual monophyletic clades) - excellent thanks for doing this, and I'm glad that it helped the overall analysis and interpretation of what is happening.
Response 10: (faster dispersal in one host-type / geographic region than another) - excellent that you can split these effects out, and thanks for the additional comments in the discussion
Response: We sincerely thank you for thoughtful and constructive feedback, which has greatly strengthened our manuscript. We are pleased that the revised analyses—including the incorporation of continental metadata (Response 7), updates to the Markov rewards (Response 8), the clade-specific analyses (Response 9), and the disentangling of host- and region-specific dispersal dynamics (Response 10)—have addressed your original concerns and enhanced the clarity and robustness of our conclusions. We truly appreciate the time and expertise you dedicated to improving this work.

Reviewer #2 (Remarks on code availability):
The data sets and scripts/code are well described in this repository, including readme and installation instructions, many thanks for making this available.
Response: Thank you for your positive feedback on the data and code availability. We are pleased that the repository and documentation were clear and useful.

Reviewer #3 (Remarks to the Author):

The authors have addressed some of our concerns, however there are a few remaining points of interest that should be addressed:

Major concerns

With respect to Reviewer #3 Response 1: We feel that the authors have not addressed the concern about showing the full temporal dynamics of the wavefront. The authors state in their response "we agree that the results from original Supplemental Figures 8-11E and F provide important context that enhances the interpretation of original Figure 7. Accordingly, these figures have been incorporated into the main text" however, the key information in those figures (the time period in which the wave front expands) has not actually been incorporated into the main text and been removed from the supplement.

Response: We apologize for the confusion in our previous response and for not fully addressing the concern regarding the presentation of the full temporal dynamics of the wavefront.

The complete temporal trajectories showing wavefront distance and diffusion coefficient from the earliest year of viral circulation to the most recent year for H7 and subtypes (H7N3, H7N7, and H7N9) have now been fully restored and are available in the revised manuscript (Fig. 5) and supplementary materials (Supplementary Figs. 9-11), respectively.

In the main text, Fig. 5 focuses on estimates dispersal statistic for H7 virus (including dispersal velocity, wavefront distance and diffusion coefficient) without considering the host. And considering the layout limitations, we only put the results of H7 in the main text, and the results of H7N3, H7N7, and H7N9 are still in the supplementary material.

And we have also added Supplementary Fig. 12 which displays a zoomed-in view (2000–2025) to better highlight recent trends of wavefront distance and diffusion coefficient.

Change in the text:

[Page 9, Lines 175-181]

The wavefront distance of H7 virus showed a consistent upward trend over time (Fig. 5E, 5K), starting near zero and rising sharply after mid-20th century. In contrast, the weighted diffusion coefficient (Fig. 5F, 5L) exhibited a fluctuating upward pattern: it stayed negligible until ~1900, then increased with significant variability and spikes sharply by 2000. H7N3, H7N7, and H7N9 also follow a similar trend of change (Supplementary Figs. 9-11). Trends in wavefront distance and diffusion coefficients over the past two decades mirrored the overall historical trends (Supplementary Fig. 12).

Change in the figure:

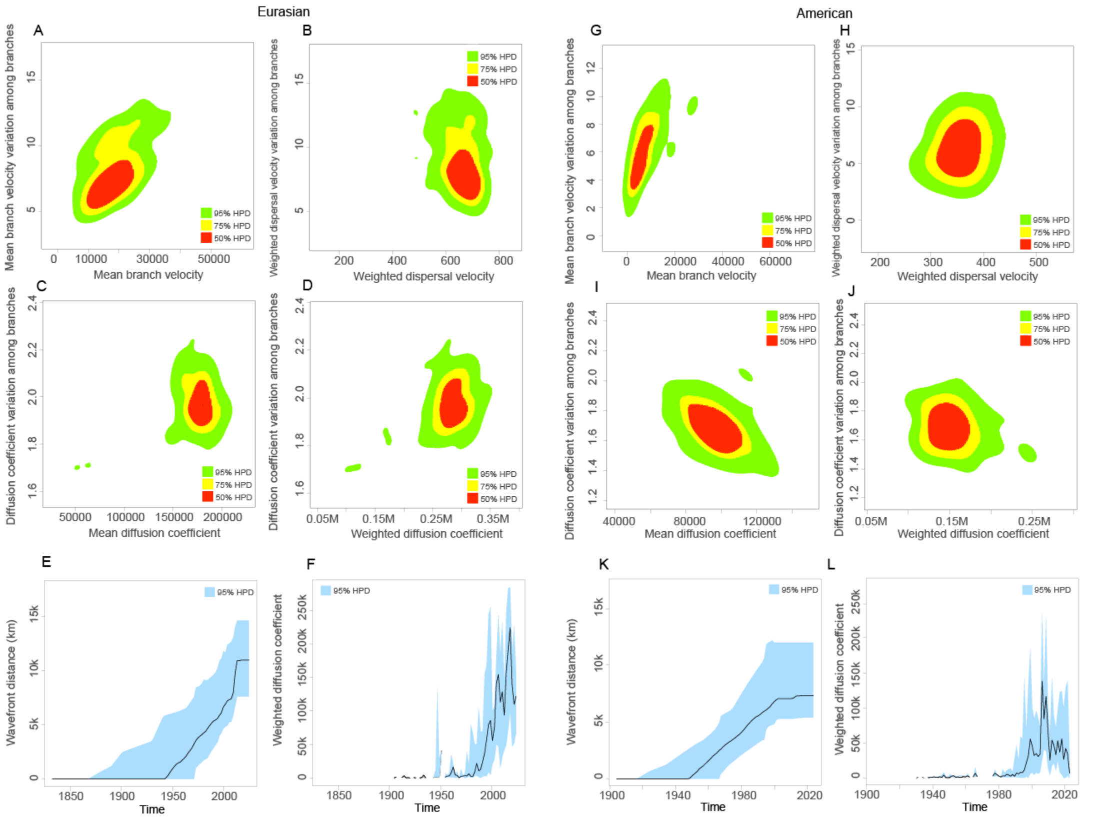

Fig. 5. Estimated dispersal statistics for H7.

The two columns on the left represent the Eurasian lineage, while the two columns on the right represent the American lineage. A and G, kernel density estimates of mean branch dispersal velocity parameters. B and H, kernel density estimates of weighted branch dispersal velocity parameters. C and I, kernel density estimates of original diffusion coefficient parameters. D and J, kernel density estimates of weighted diffusion coefficient parameters. E and K, furthest extent of epidemic wavefront (spatial distance from epidemic origin). F and L, Evolution of weighted diffusion coefficient.

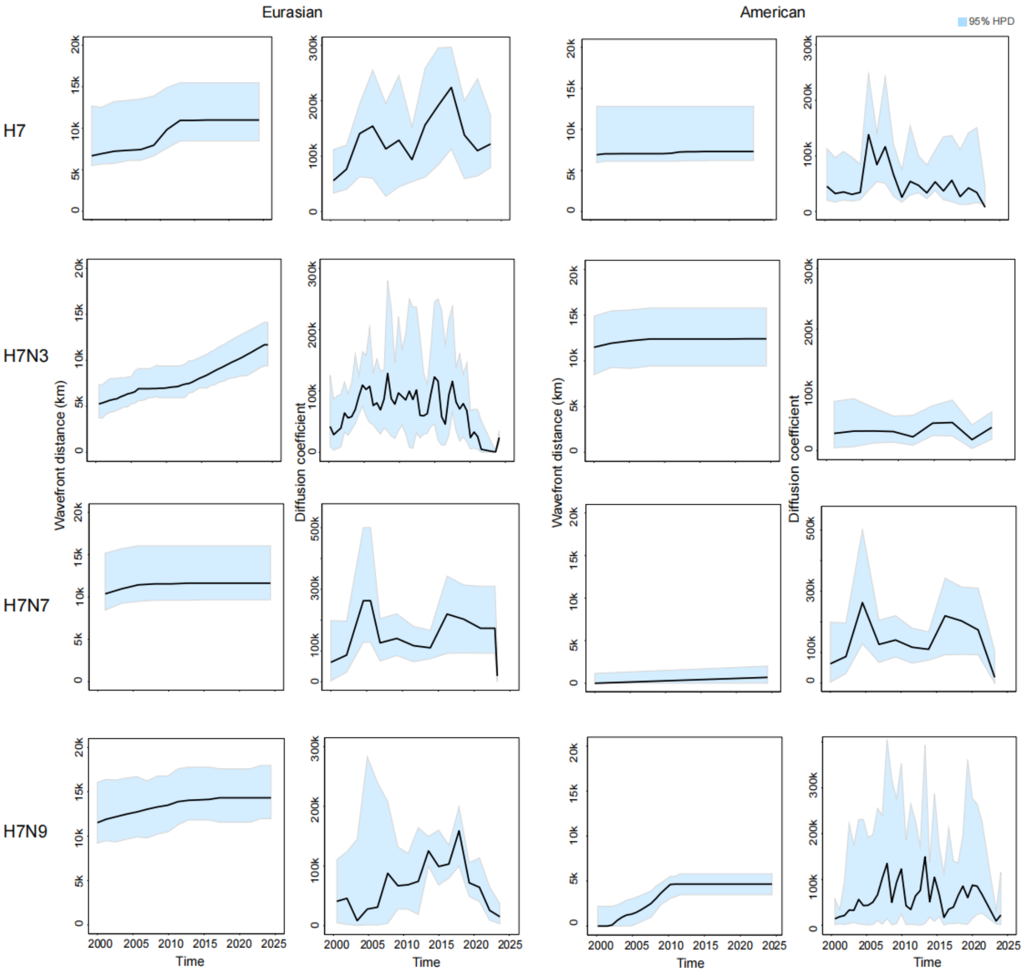

Supplementary Fig. 12. Estimated dispersal statistics of wavefront distance and diffusion coefficient for H7 and subtypes since 2000.

Regarding Response 7 there is missing analysis to support some of the discussed results about the deeper phylogeographic history in the manuscript. Line 28 and Line 112-113: there are currently no inferences shown in figures or tables that support Asia as the source H7; and the inference of geographic divergence discussed in Line 119-127 is not actually shown in fig 2. Temp Fig 1 in response to reviewers shows the phylogeographic inference.

Response: We sincerely thank you for this critical comment. We apologize for the earlier lack of clarity, and appreciate the reviewer's keen eye in helping us strengthen the presentation of our phylogeographic results.

Indeed, in our study, Fig. 2 was designed to depict the evolutionary characteristics of H7 viruses and subtypes, integrating additional metadata such as host species and subtype information; while Temp Figure 1 highlights geographic state more explicitly, which better illustrates the inferred ancestral locations.

To address the concern, we have now included Supplementary Fig. 6 (formerly Temp Figure 1). Supplementary Fig.6 labels the root node and early ancestral branches with "Asia" and provides posterior probabilities (e.g., root state posterior probability = 1), supporting our inference of an Asian origin.

We also added explicit references to this supplementary figure in corresponding sentences to support our statements about the Asian origin and subsequent spatial divergence.

Change in the text:

[Page 6, Lines 114-121]

Current circulating H7 lineages appear to have spread from Asia to Europe and Africa, while North American sequences likely represent an independent gene pool (Supplementary Fig. 6). The phylogeographic reconstruction suggested that H7 viruses most likely originated in Asia (root state posterior probability = 0.84) (Supplementary Fig. 6A). The root of the H7N3 and H7N9 clades was inferred to be in Asia with posterior probability = 1.0 (Supplementary Fig. 6B, 6D), providing strong support for an Asian origin. In contrast, H7N7 most likely originated in Europe, with a root state posterior probability of 0.9927 (Supplementary Fig. 6C).

[Page 7, Lines 135-136]

The information of subtype, host, continent and pathogenicity was presented in Fig. 2.

Change in the figure:

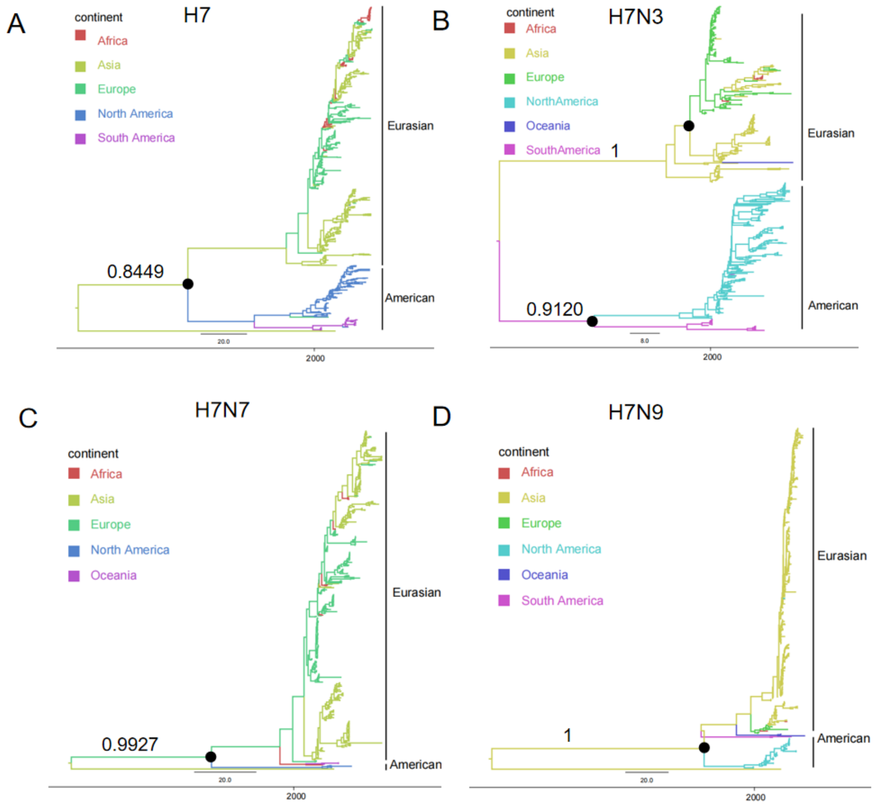

Supplementary Fig. 6. Time-scaled maximum clade credibility (MCC) trees of H7 HA genes sequences plotted by FigTree software, with branch colors representing geographic information.

Minor concerns

Additionally, with respect to Reviewer #3 Response 1, the authors summarized the wavefront focusing on final distance in Fig 8a; a similar approach could be taken for Fig 8b, given that the temporal dynamics of the diffusion coefficient are also not addressed in the text.

Response: We thank you for this constructive suggestion. Following your recommendation, we have added a new panel (Fig. 8B1) showing the final diffusion coefficient values without introduce the element of time, analogous to the final distance summary in Fig. 8A1.

Additionally, to more comprehensively present the temporal dynamics of wavefront distances and diffusion coefficient, the time-included panels for wavefront distance are also presented in Fig. 8A2, 8A3, and the time-included panels for the diffusion coefficient are shown in Fig. 8B2, 8B3. The corresponding description has been added to the Results section.

We sincerely appreciate your thoughtful and constructive feedback. If the revised presentation in Fig. 8 still fall short of expectations, particularly if removal of Fig. 8A2, 8A3 and Fig. 8B2, 8B3 is recommended, we would be most grateful for the opportunity to make further revisions.

Change in the text:

[Page 11, Lines 220-233]

Wavefront distance varied by host type: in the latest estimates, the Eurasian lineage of H7 exhibited a wavefront distance of 12,096.978 km (7,901.05 - 12,096.978) in Anseriformes, exceeding that of Galliformes (9,609.90 km, 8,097.98 - 11,927.54), and a similar pattern was observed for the American lineage (Fig. 8A1). Notably, the American lineage of H7 virus exhibited longer wavefront distance in domestic birds than wild birds, while whereas the opposite was observed for the Eurasian lineage (Fig. 8A1). From the perspective of temporal trends, Anseriformes and wild birds played a more prominent role in the dissemination of the H7 virus, as is also the case for subtypes (Fig. 8A2, 8A3). Overall, the result of final diffusion coefficient also showed Anseriformes and wild birds play a more important role in H7 transmission (Fig. 8B1). The diffusion coefficient was consistently higher for viruses originating from Anseriformes than from Galliformes for H7 and subtypes (Fig. 8B2). The diffusion coefficient of H7 virus among wild and domestic birds was similar (Fig. 8B3). The variation trend of H7N3 and H7N9 diffusion coefficient is consistent, while the diffusion coefficient of H7N7 was higher in domestic birds than in wild birds during a specific period (Fig. 8B2, 8B3).

Change in the figure:

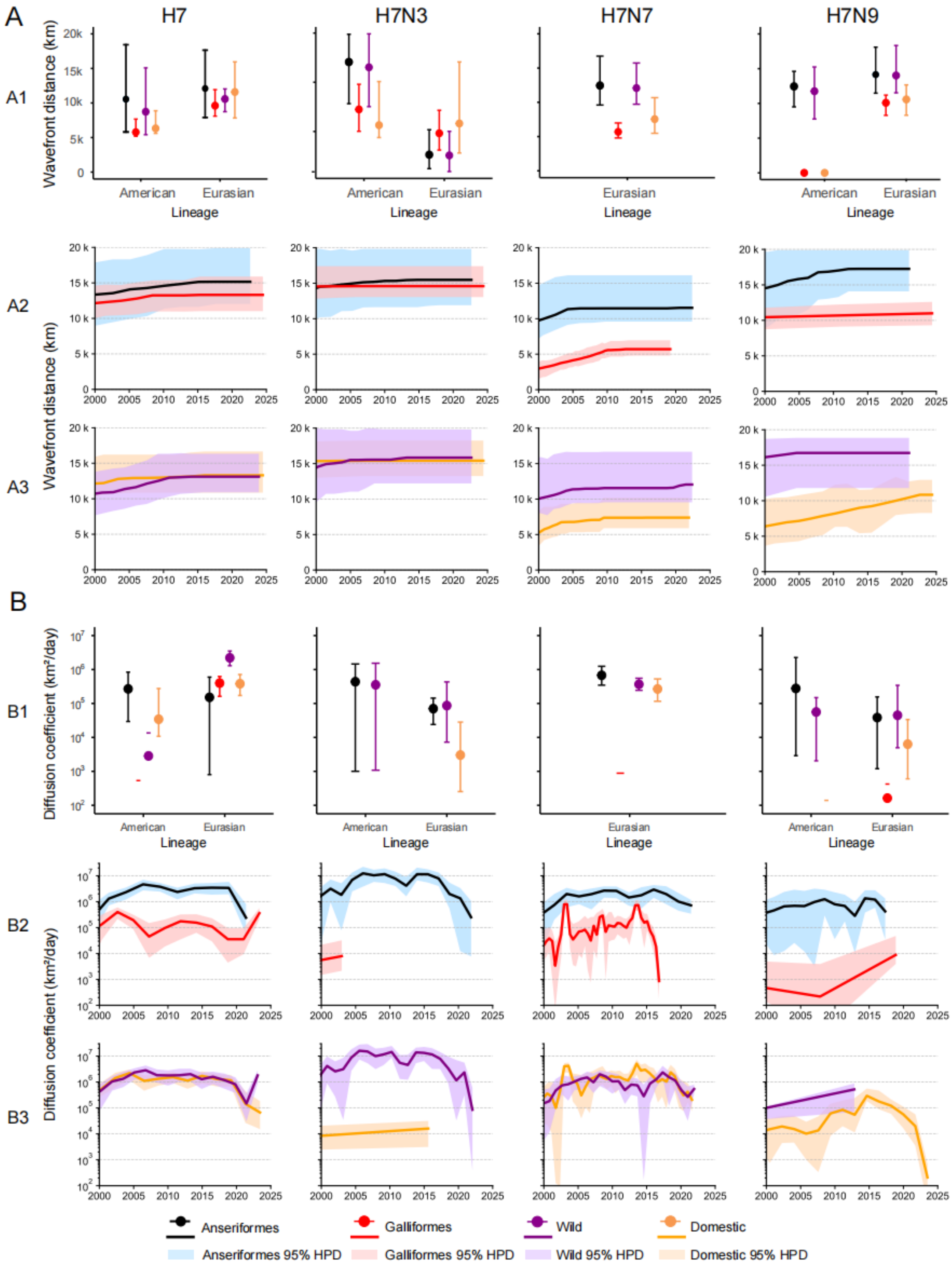

Fig. 8. Host-specific wavefront distance and diffusion coefficient estimates for H7 and its subtypes. A. Estimated wavefront distances (km) for H7 virus and subtypes. A1 shows the final spatial extent of spread for different viral lineages, while A2 and A3 illustrate the temporal dynamics of wavefront distances without considering lineages; B. Weighted diffusion coefficient (km²/day). (1) presents the final diffusion coefficient, and B2 and B3 illustrate the temporal dynamics of diffusion coefficient without considering lineages. Shaded areas represent 95% highest posterior density (HPD). Diffusion estimates for certain years are omitted due to irregular values likely resulting from limited sampling.

With respect to the changes made in Response 6, in the updated manuscript Line 112, the phrase "unidirectional evolution" is unclear, "unidirectional transmissions events" would be understood.

Response: We thank you for this helpful suggestion. We agree that "unidirectional transmissions events" is more precise than "unidirectional evolution" in this context. We have revised accordingly in the updated manuscript (Line 114).

Reviewer #4 (Remarks to the Author):

I co-reviewed this manuscript with one of the reviewers who provided the listed reports. This is part of the Nature Communications initiative to facilitate training in peer review and to provide appropriate recognition for Early Career Researchers who co-review manuscripts.

Response: We sincerely appreciate the time and expertise that the co-reviewers have dedicated to evaluating our manuscript. We fully support Nature Communications' initiative to facilitate training in peer review and to provide appropriate recognition for Early Career Researchers who co-review manuscripts.
